# Effectiveness of Smartphone-Based Mindfulness Training on Maternal Perinatal Depression: Randomized Controlled Trial

**DOI:** 10.2196/23410

**Published:** 2021-01-27

**Authors:** Yaoyao Sun, Yanyan Li, Juan Wang, Qingyi Chen, Alessandra N Bazzano, Fenglin Cao

**Affiliations:** 1 School of Nursing and Rehabilitation Shandong University Jinan China; 2 Tulane University School of Public Health and Tropical Medicine New Orleans, LA United States

**Keywords:** mindfulness, pregnancy, perinatal care, depression, mHealth, mobile health, psychosocial intervention

## Abstract

**Background:**

Despite potential for benefit, mindfulness remains an emergent area in perinatal mental health care, and evidence of smartphone-based mindfulness training for perinatal depression is especially limited.

**Objective:**

The objective of this study was to evaluate the effectiveness of a smartphone-based mindfulness training intervention during pregnancy on perinatal depression and other mental health problems with a randomized controlled design.

**Methods:**

Pregnant adult women who were potentially at risk of perinatal depression were recruited from an obstetrics clinic and randomized to a self-guided 8-week smartphone-based mindfulness training during pregnancy group or attention control group. Mental health indicators were surveyed over five time points through the postpartum period by online self-assessment. The assessor who collected the follow-up data was blind to the assignment. The primary outcome was depression as measured by symptoms, and secondary outcomes were anxiety, stress, affect, sleep, fatigue, memory, and fear.

**Results:**

A total of 168 participants were randomly allocated to the mindfulness training (n=84) or attention control (n=84) group. The overall dropout rate was 34.5%, and 52.4% of the participants completed the intervention. Mindfulness training participants reported significant improvement of depression (group × time interaction χ^2^_4_=16.2, *P*=.003) and secondary outcomes (χ^2^_4_=13.1, *P*=.01 for anxiety; χ^2^_4_=8.4, *P*=.04 for positive affect) compared to attention control group participants. Medium between-group effect sizes were found on depression and positive affect at postintervention, and on anxiety in late pregnancy (Cohen d=0.47, –0.49, and 0.46, respectively). Mindfulness training participants reported a decreased risk of positive depressive symptom (Edinburgh Postnatal Depression Scale [EPDS] score>9) compared to attention control participants postintervention (odds ratio [OR] 0.391, 95% CI 0.164-0.930) and significantly higher depression symptom remission with different EPDS reduction scores from preintervention to postintervention (OR 3.471-27.986). Parity did not show a significant moderating effect; however, for nulliparous women, mindfulness training participants had significantly improved depression symptoms compared to nulliparous attention control group participants (group × time interaction χ^2^_4_=18.1, *P*=.001).

**Conclusions:**

Smartphone-based mindfulness training is an effective intervention in improving maternal perinatal depression for those who are potentially at risk of perinatal depression in early pregnancy. Nulliparous women are a promising subgroup who may benefit more from mindfulness training.

**Trial Registration:**

Chinese Clinical Trial Registry ChiCTR1900028521; http://www.chictr.org.cn/showproj.aspx?proj=33474

## Introduction

The perinatal period is a major transitional stage in the life course, accompanied by social, emotional, physical, and hormonal changes, and is consequently an important period for mental health problems [1]. Symptoms of perinatal psychological distress are experienced by a large number of women with approximately 5%-30% affected by depressive symptoms [2]. Perinatal depression, a common nonpsychotic depressive disorder, occurs through pregnancy to the postpartum phase, and has serious consequences for maternal [3], infant, and childhood outcomes [4], as well as economic costs [5]. Low-and middle-income countries (LAMICs) reportedly have a higher prevalence of prenatal depression than high-income countries (19%-25% vs 7%-15%) [4]. However, the prevention and treatment of perinatal depression in LAMICs remains underrecognized, in part due to limited resources for mental health services, greater priority placed on preventing obstetric complications and fetal anomaly [6], and fear of stigmatization [7]. In this context, provision of low-cost and effective mental health services in LAMICs represents a potentially significant strategy to improve maternal and child health over the life course.

Difficulty in accessing health services has been cited as a reason for the low uptake of mental health interventions in previous studies [8,9]. The pervasive availability of smartphones provides an appropriate platform to address this problem and improve accessibility to mental health services [10]. Smartphone apps were reported to be helpful for treating depression in a recent systematic review and meta-analysis [11]. Another meta-analysis of randomized controlled trials (RCTs) including 22 apps also illustrated the promise of smartphones as a self-management tool for depression, with effect sizes ranging from 0.22 to 0.56 [12]. Smartphone-based interventions may be particularly useful and applicable for perinatal populations because recent studies have found that a large proportion of women seek pregnancy-related information through the internet [13,14].

Among the popular mental health smartphone apps, mindfulness was considered the most common evidence-based strategy [10] and one of the most frequently used modalities in apps for depression treatment [15]. Mindfulness is defined as “paying attention in a particular way: on purpose, in the present moment, and non-judgmentally” [16], and contains seven fundamental attitudinal factors: nonjudging, nonstriving, beginner’s mind, patience, trust, acceptance, and letting go [17]. Mindfulness-based interventions (MBIs) have now been widely used for reducing depressive symptoms, relieving psychological distress, and improving wellness within a broad range of populations [18]. For pregnant women, prenatal MBIs such as the Mindfulness-Based Childbirth and Parenting [19] and MindBabyBody [20] programs have been utilized to reduce maternal depression, anxiety, and negative affect [21]; enhance maternal nurturing behaviors; and improve childhood outcomes [22]. MBIs support individuals to alter intrinsic thought patterns, explore mind-body connections, and develop behavior modifications [23], which could be particularly appropriate for pregnant women facing physical changes and social role adjustment. Some reviews have reported the increasing use of MBIs in the perinatal period and their potential benefits for improving perinatal depression [23,24]. However, the evidence in this emerging area is still limited because the majority of research performed thus far has included small sample sizes and nonrandomized designs [21,25]. Moreover, few studies have been performed in LAMICs with most of these studies performed in high-income countries [25].

Over the last few years, internet-based MBIs and those accessed through apps have been increasingly considered for perinatal care. Online MBIs among nonpregnant women have shown small but significant effects on depression improvement with Hedges *g* of 0.29 [18]. However, the related literature for the pregnant population is sparse. Reported studies have been preliminary [26-29], aimed only at the postpartum period [30], treated mindfulness as a single component of a complex integrated intervention [31], or used only a pre-post test design [32]. Nevertheless, some pilot studies have supported online MBI as a promising technique to help expectant mothers reduce depressive symptoms [27,28].

More rigorous investigation of the effectiveness of online MBIs in the perinatal period has been lacking, and the need for a well-designed RCT to test the effectiveness in a larger sample and with longer-term follow up was identified. Thus, we carried out an 8-week self-guided smartphone-based mindfulness training intervention using an RCT with 32-week follow up and an attention control group from the early second trimester to the postpartum period among pregnant women deemed to be at risk of perinatal depression. The primary aim of this study was to evaluate the overall intervention effect on perinatal depression symptoms between the mindfulness training in pregnancy group and the attention control group. The effectiveness of mindfulness training in pregnancy on secondary outcomes (anxiety symptoms, perceived stress, positive and negative emotions, fatigue, sleep-related problems, memory, and fear of childbirth) were also explored. Further, we compared the effects of mindfulness training on depression remission at postintervention between the two groups. Finally, we tried to explore whether the intervention effects would differ between nulliparous and multiparous women.

## Methods

### Trial Design

This study was a single-center, two-parallel-armed, assessor-blinded, 1:1-allocated RCT with a 32-week follow up. Pregnant women who scored at or above the threshold for positive depressive symptoms on the Edinburgh Postnatal Depression Scale (EPDS) or Patient Health Questionnaire-9 (PHQ-9) were randomly assigned to the mindfulness intervention group (receiving 8-week smartphone-based mindfulness training) or attention control group (receiving 8-week regular WeChat health consultations). This study was approved by the ethical review board of the School of Nursing, Shandong University (2018-R-015). The trial was registered in the Chinese Clinical Trial Registry (ChiCTR1900028521) in December 2019 and no significant changes were made between the start of the trial and the registration confirmation.

### Participants

The study was conducted between March 2018 and January 2020, and participants were recruited in the obstetrics clinic of a tertiary hospital in Jinan, Shandong, a city located in the east of China. The hospital provides perinatal services for around 5000 pregnant women each year.

Inclusion criteria for women to participate in the study included: (1) aged 18 years and over, (2) in the 12th to 20th week of gestation, (3) singleton pregnancy, (4) no plan to terminate pregnancy, (5) planned to receive antenatal examination and deliver in the study hospital, (6) completed junior high school education or above, (7) positive in depressive symptoms screening with an EPDS score>9 or a PHQ-9 score>4, (8) able to use the app on a smartphone for the study, and (9) able to understand and respond to the questionnaire.

Exclusion criteria were: (1) at risk of suicide or self-harm, (2) currently receiving psychiatric treatment or using psychiatric medications, (3) history of substance abuse or addiction in the past 6 months, (4) prior experience with mindfulness meditation, and (5) declined to participate.

### Procedure

According to the national health policy in China, health status for all pregnant women is recorded in the 12th gestational week and they start prenatal visits regularly thereafter. Pregnant women were recruited from the records of the tertiary hospital where the study was based, during the time they are ordinarily required to report pregnancy-related information and receive preventive depression screening at their first regular visit in the obstetrics clinic. Informed consent for preventive psychological assessment was first obtained, and once consent was granted, printed questionnaires, including sociodemographics, pregnancy-related characteristics, and mental health indicators, were distributed for participants to complete and return. All participant records were screened according to the inclusion and exclusion criteria, and eligible participants were then contacted by a research assistant through telephone or WeChat (a popular instant social communicating software in China) within 2 weeks. Online informed consent for participation in the RCT was obtained through the online survey platform Wjx.cn. The RCT program was introduced to potential participants while discussing results of their preventive psychological assessment. This initial baseline evaluation of symptoms was classified as T1 for consented trial participants. The procedure and objectives of the trial were explained, and then online informed consent was obtained if they agreed to participate.

Recruited participants were randomly assigned to the mindfulness training during pregnancy or attention control group; the mindfulness group received 8-week mindfulness training and the control group received 8-week regular WeChat health consultation to control attention. Participants who were allocated to the mindfulness training group received the URL of the app through WeChat. They could use the app as long as they signed up. Data collection and assessment of outcomes took place over four time points in the follow-up period. T2 assessment took place at 4 weeks after allocation (intermediate period of intervention), T3 assessment took place at 8 weeks after allocation (endpoint of the intervention), T4 took place at 18 weeks after allocation (before childbirth), and T5 took place at 6 weeks after delivery. Follow-up assessments were collected by computer/smartphone-assisted self-administered surveys. All participants were awarded 2 yuan (US $0.30) when completing an assessment.

### Intervention

#### Mindfulness Training During Pregnancy Group

The mindfulness training program was revised from Mindfulness Behavioral Cognitive Therapy (MBCT) developed by John Teasdale, Mark Williams, and Zindel Segal [33]. A psychologist with 5 years of mindfulness experience led the adaptation of the mindfulness training in pregnancy course. One obstetrician, one obstetric nurse, and two research assistants with mindfulness experience participated. Three principles guided the adaptation process: focus on perinatal depression and negative emotions, make physical adaptations for pregnant women, and simplify and shorten the practice properly, with each formal training limited to 25 minutes. The 8-week mindfulness training program contained eight sessions: week 1, *Understand mindfulness;* week 2, *Be in the present;* week 3, *Be mindful of negative emotions;* week 4, *Accept difficulties;* week 5, *Thoughts are just thoughts;* week 6, *Enjoy daily happiness;* week 7, *Mindful pregnancy and childbirth;* and week 8, *Continued mindfulness practice*. Each session was composed of thematic curriculum as well as formal and informal training lasting for 1 week. The thematic curriculum was provided through text, audio, and visual materials at the beginning of each session. Formal mindfulness training techniques were then introduced, and users were invited to continue practice, following the recordings and writing in the mindfulness journal, for 6 days per week. Formal mindfulness training included body scan, mindful breathing, mindful stretching, and mindful meditation lasting 15-25 minutes per day. Informal training was also recommended to be practiced every day, including pausing in the midst of daily life, mindful eating, mindful walking, and 3-minute breathing practices. Details on the components of the mindfulness training in pregnancy course are provided in Table 1.

**Table 1:** Content of mindfulness training during pregnancy intervention.

Week	Subject	Thematic curriculum	Formal training	Informal training
1	Understand mindfulness	Risks of negative emotions in pregnancy What is mindfulness and how can it be useful for addressing symptoms Raisin exercise	Body scan	Pausing in the midst of daily life
2	Be in the present	Being and Doing models Be in the present Unpleasant events journal	Mindful breathing	Mindful eating and mindful walking
3	Be mindful of negative emotions	Recognize the habitual stress response List of negative thinking 3-minute breathing space	Mindful breathing Mindful stretching	3-minute breathing space
4	Accept difficulties	Difficult communication journal Learn to accept difficulties	Mindful meditation (be with difficulties)	3-minute breathing space
5	Thoughts are just thoughts	Recognize thoughts without judgment Let thoughts be	Mindful meditation (be with thoughts)	3-minute breathing space
6	Enjoy daily happiness	Be mindful of happiness Pleasant events journal	Body scan	Pausing in the midst of daily life
7	Mindful pregnancy and childbirth	Be mindful of emotions caused by pregnancy Feel fetal movement mindfully	Body scan Mindful stretching (childbirth)	3-minute breathing space
8	Continued mindfulness practice	Discussion on awareness of emotions and stress responses Consider continued mindfulness practice	Self-directed	Self-directed

**Figure 1:**
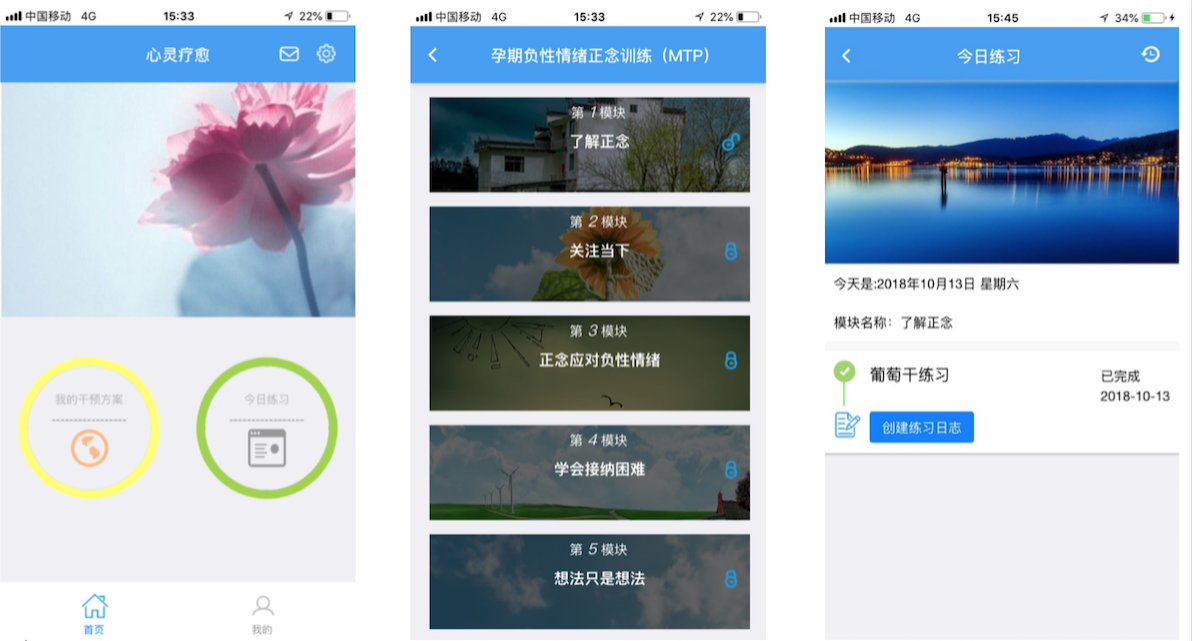
Screenshots of Spirits Healing mindfulness app.

The mindfulness training program was delivered through a custom-built mobile app called *Spirits Healing* in Chinese. It was available in both the Android and iOS operating systems in mainland China. The *Spirits Healing* app provided reading materials, recordings for guided practice, and videos. Participants were able to navigate contents and make notes in the app. The mindfulness training program automatically updated every day and participants practiced according to their own schedules. A message to remind participants to utilize the mindfulness training program was sent every week by WeChat. Participants were awarded 2 yuan (US $0.30) for completion of each week of training. The app was debugged three times during the trial due to adaptation of phone systems, but no changes related to intervention content were made. For safety and destigmatization, participants were reminded that this app is not equivalent to psychotherapy and were referred to professional support when necessary. Visual representations of the app content are shown in Figure 1. Additional details relating to the intervention construction process can be found in Multimedia Appendix 1.

#### Attention Control Group

Pregnant women allocated to the attention control group received 8-week regular WeChat health consultations as an attention control. Health consultations were provided by a clinically trained nursing assistant with experience in prenatal care using the WeChat app. The schedule for routine prenatal care was sent to participants at the time they were assigned to their group. The nursing assistant contacted participants in the attention control group once every week for 8 weeks to ask about recent health status using the following script: “Hello, Ms. X. How are you feeling this week?” The content of the consultations included discussion of recent medical examinations, outpatient appointments, and assistance with arrangements for inpatient care.

### Measures

Depression symptoms as assessed by the EPDS was the primary outcome in this study. The EPDS is a self-report scale that assesses depressive symptoms experienced within the last week during both prenatal and postnatal periods [34]. The EPDS contains 10 items with responses on a 4-point Likert scale ranging from 0 to 3, where higher scores represent greater intensity of a depressive symptom. The EPDS was recommended as a valid depression screening tool across different cultures and different trimesters in a validation study review [35], and the cut-off score of 9/10 was used to identify positive depressive symptoms for screening purposes in this study. Additionally, another commonly used depression screening tool, the PHQ-9 developed on the basis of Diagnostic and Statistical Manual of Mental Disorders-IV criteria, was also used in the screening period with a cutoff of 4/5 indicative of positive depression criteria [36].

Secondary outcomes consisted of multidimensional health issues for perinatal women, including anxiety symptoms, perceived stress, positive affect, negative affect, sleep-related problems, fatigue, prospective memory, retrospective memory, and fear of childbirth. Anxiety in the previous 2 weeks was evaluated by the 7-item Generalized Anxiety Disorder scale, a clinically useful assessment for detection of symptoms of anxiety in the perinatal period [37]. We assessed perceived stress of pregnant women using the 4-item validated Perceived Stress Scale to measure the degree in the past month that their situation was appraised as uncontrollable, unpredictable, and overwhelming [38]. The Positive and Negative Affect Schedule [39] was used to measure individuals’ agreement and endorsement on statements related to positive and negative affect. Sleep-related problems in the past month were evaluated by the self-administered Pittsburgh Sleep Quality Index questionnaire [40] and the degree of fatigue in the past week was evaluated by the 9-item Fatigue Severity Scale [41]. Subjective prospective and retrospective memory failures in daily life were self-rated by participants using the Prospective and Retrospective Memory Questionnaire [42]. In addition, the Wijma Delivery Expectancy Questionnaire [43] with items related to women’s cognitive appraisal regarding the delivery process was used to measure the level of fear of childbirth. For all measurements other than positive affect, higher scores represent worse mental health outcomes, and for positive affect, higher scores represent a higher positive affect. Time points for the outcome indicators assessed are shown in Multimedia Appendix 2.

Sociodemographic characteristics such as age, gestational age, BMI, education level, work status, marital status, family economic status, and pregnancy-related characteristics were self-reported by the participants at baseline (T1). Study researchers were allowed to collect additional clinical data on participants from medical records following birth.

### Sample Size

Previous meta-analyses indicated that mindfulness practice during pregnancy reduced depression scores with an effect size of 0.59 [44] and self-help mindfulness resulted in small to medium effect sizes on anxiety/depression [45]. On the basis of a medium intervention effect (Cohen *d*=0.50), an estimated sample size of 128 was required to compare between-group differences on depression at postintervention with 80% statistical power, a 1:1 allocation rate, and a two-tailed significance level of .05. In the above-mentioned meta-analysis of self-help mindfulness [45], on average, 27% of the participants were lost to follow up in the postintervention assessment. Considering a 30% attrition rate, a final sample of 168 with 84 individuals in each group was required. The sample size was calculated using G*Power [46].

### Randomization and Blinding

The trial used a simple randomization approach. The random number sequence was generated by a researcher who did not participate in this study using the Random Number Generators function in SPSS 23.0. The sequence was kept in sealed, opaque, numbered envelopes. When each participant was enrolled, a research assistant opened the envelope in sequence and assigned the participant to the group. Participants received allocation according to the order of enrollment. Throughout the RCT procedure, the assessor who collected follow-up data did not know the assignment. Considering the use of the smartphone app only in the mindfulness intervention, the participants were able to infer the assignment.

### Intervention Fidelity

Logs of practice on formal mindfulness training were recorded and used to evaluate the fidelity of mindfulness training. At least 3 days of practice per week was considered a completed training week, and at least 4/8 completed training weeks were considered as completion of the mindfulness training program. The completion rate in this study was calculated as the percentage of participants who completed the mindfulness training divided by the number of participants who received the intervention.

### Statistical Analysis

Statistical analyses were performed with SPSS 23.0. The primary analysis used an intention-to-treat (ITT) approach. Normality of outcomes at baseline was visually examined, and negative affect, sleep, and positive affect were log-transformed due to non-normal data distributions. No more than 5 individuals for each measure at T1 had a single entry missing, which were considered as missing at random and were imputed with the median of the relevant item. Independent sample *t* tests and *χ*^2^ tests were used to compare the baseline characteristics between the intervention and control groups, and between the study sample and dropout sample. To assess the population-averaged mindfulness training vs attention control intervention effect on outcomes, generalized estimating equations (GEEs) were formulated. This approach has been recommended because it is able to handle missing data appropriately and remains stable in different correlation matrices [47]. For GEEs, the participants and assessment time points were designated as subject variables and within-subject variables, respectively, with an exchangeable working correlation matrix and full maximum-likelihood estimation applied. The continuous outcome variables at five time points were the dependent variables. Main effects of group, time, and group × time interaction effects were examined. Testing of a simple main effect for group was also explored by examining differences between groups at each time point. To further assess the intervention effect on depression symptoms remission postintervention (T3), binary positive depressive symptoms at T3 and EPDS reductions from T1 to T3 were compared between mindfulness and attention control groups using logistic regression models, adjusting for the EPDS score at baseline and between-group imbalanced factor (intended pregnancy) after randomization. In addition, subgroup analysis was also conducted to explore the impact of parity (primipara/multipara) on the intervention effect.

Effect sizes are presented as Cohen *d* based on the ITT rule. Cohen *d* was calculated between groups and follow ups referring to baseline data. Effect sizes were considered to be small (*d*=0.2), medium (*d*=0.5), and large (*d*=0.8). A two-sided *P* value less than .05 in the primary analysis and less than .025 in the subgroup analysis after Bonferroni multiple-comparison correction were considered statistically significant.

As suggested by Thabane [48], several sensitivity analyses were performed. First, the mindfulness training vs attention control intervention effect was analyzed according to per-protocol group and as-treated group comparisons in addition to the ITT group. Second, adjusted GEE models with a baseline imbalanced factor (intended pregnancy) were also established. Third, intervention effects were also evaluated in participants who completed different numbers of follow-up assessments. Details of the statistical analysis are provided in Multimedia Appendix 3.

## Results

### Recruitment and Participant Flow

**Figure 2:**
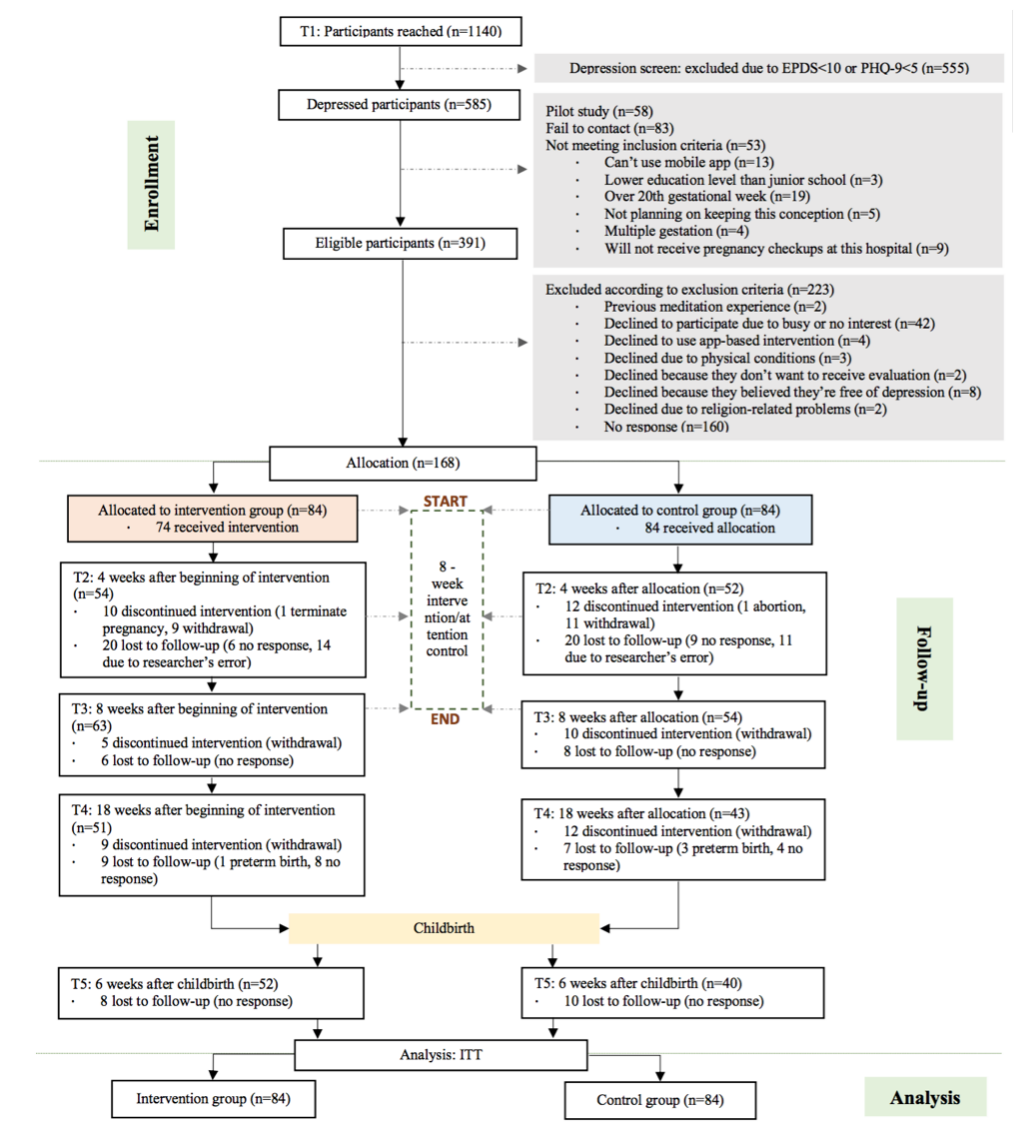
Participant flow chart. EPDS: Edinburgh Postnatal Depression Scale; PHQ-9: Patient Health Questionnaire-9; ITT: intention to treat.

Recruitment began in March 2018 and ended in June 2019, and follow-up assessments ended in January 2020. Figure 2 illustrates the full study flow diagram. A total of 1140 pregnant women were contacted and a final sample of 168 was allocated. During the whole follow-up period for the 168 participants, 24/84 (29%) participants dropped out in the mindfulness training group and 34/84 (41%) dropped out in the attention control group with the overall dropout rate reaching 34.5%. More than half of the participants completed at least 3 follow ups (94/168, 55.6%) and 56/168 (33.3%) of participants completed all follow ups. A logistic regression model with binary dropout status as the dependent variable showed that participants with an advanced gestational age at baseline tended to drop out more frequently during follow up (odds ratio [OR] 1.033, 95% CI 1.004-1.063; see Multimedia Appendix 4).

### Baseline Data

Table 2 shows the details of participant characteristics. Overall, all of the pregnant women were married, with an average age of around 30 years, and were at 99 gestational days (around 14 gestational weeks) at baseline. A total of 41.1% (69/168) of the participants screened positive in the EPDS and 92.3% (155/168) screened positive for depression symptoms in the PHQ-9. The mean gestational week was 16.8 (SD 1.068) at randomization. There was no significant difference between the mindfulness training and attention control groups at baseline except for intended pregnancy. More pregnant women planned this pregnancy in the mindfulness training group than in the attention control group (*χ*^2^_1_=8.4, *P*=.004).

**Table 2:** Participant characteristics at baseline.

Characteristics		Total (N=168)		ACG^a^ (n=84)		MTPG^b^ (n=84)			*P* value
		N	Value	N	Value	N	Value		
Age (years), mean (SD)		168	29.91 (4.015)	84	29.55 (4.21)	84	30.27 (3.80)	.24	
**Maternal age category, n (%)**		168						.68	
	Not advanced (18-34 years)		140 (83.3)		71 (85)		69 (82)		
	Advanced (>35 years)		28 (16.7)		13 (16)		15 (18)		
Gestational age (days), mean (SD)		168	98.81 (14.726)	84	100.85 (15.180)	84	96.77 (14.054)	.07	
Weight gain, mean (SD)		156	1.77 (2.989)	77	1.98 (2.916)	79	1.57 (3.063)	.39	
BMI before pregnancy, mean (SD)		162	21.81 (3.091)	79	21.44 (3.361)	83	22.16 (2.786)	.14	
BMI now, mean (SD)		160	22.41 (2.935)	81	22.07 (2.994)	79	22.76 (2.850)	.14	
**Race, n (%)**		168		84		84		.49	
	Han		167 (99.4)		83 (99)		84 (100)		
	Hui		1 (0.60)		1 (1)		0 (0)		
Education years, mean (SD)		165	15.42 (2.361)	81	15.17 (2.323)	84	15.67 (2.386)	.18	
**Work status, n (%)**		163		80		83		.22	
	Unemployed		38 (23.3)		22 (28)		16 (19)		
	Employed		125 (76.7)		58 (72)		67 (81)		
**Family monthly income per person (US $)^c^, n (%)**		152		74		78		.29	
	<309		1 (0.7)		1 (1)		0		
	309-618		32 (21.1)		19 (26)		13 (17)		
	618-926		46 (30.3)		19 (26)		27 (35)		
	≥926		73 (48.0)		35 (47)		38 (49)		
Married, n (%)		164	164 (100)	81	81 (100)	83	83 (100)		
**Parity, n (%)**		168		84		84		.63	
	Primipara		109 (64.9)		53 (63)		56 (67)		
	Multipara		59 (35.1)		31 (37)		28 (33)		
**History of abortion, n (%)**		168		84		84		.53	
	Yes		74 (44.0)		39 (46)		35 (42)		
	No		94 (56.0)		45 (54)		49 (58)		
**History of induced labor, n (%)**		168		84		84		.50	
	Yes		9 (5.4)		3 (4)		6 (7)		
	No		159 (94.6)		81 (96)		78 (93)		
**History of embryo damage, n (%)**		168		84		84		.17	
	Yes		33 (19.6)		13 (16)		20 (24)		
	No		135 (80.4)		71 (84)		64 (76)		
**Intended pregnancy, n (%)**		162		80		82		.004	
	Yes		125 (77.2)		54 (68)		71 (87)		
	No		37 (22.8)		26 (32)		11 (13)		
Severity of early pregnancy reaction^d^, mean (SD)		150	2.70 (1.394)	71	2.62 (1.398)	79	2.77 (1.395)	.51	
**History of previous disease, n (%)**		161		81		80		>.99	
	Yes		10 (6.2)		5 (6)		5 (6)		
	No		151 (93.8)		76 (94)		75 (94)		
EPDS^e^, mean (SD)		168	8.27 (4.245)	84	8.55 (4.593)	84	7.99 (3.873)	.40	
PHQ-9^f^, mean (SD)		168	7.73 (3.295)	84	7.50 (3.036)	84	7.96 (3.538)	.36	
GAD-7^g^, mean (SD)		146	5.40 (3.239)	73	4.99 (3.204)	73	5.81 (3.243)	.13	
PSS^h^, mean (SD)		147	5.26 (2.315)	73	5.33 (2.267)	74	5.19 (2.374)	.72	
PA^i^, mean (SD)		143	27.29 (5.227)	71	26.85 (5.255)	72	27.72 (5.198)	.32	
NA^j^, mean (SD)		143	20.63 (5.734)	71	19.96 (5.111)	72	21.29 (6.254)	.17	
PSQI^k^, mean (SD)		139	6.85 (2.980)	72	6.58 (2.577)	67	7.13 (3.357)	.28	
FSS^l^, mean (SD)		145	43.16 (9.179)	72	43.78 (8.899)	73	42.55 (9.469)	.42	
PM^m^, mean (SD)		145	20.65 (6.017)	73	20.93 (6.106)	72	20.36 (5.954)	.57	
RM^n^, mean (SD)		145	19.54 (6.059)	73	19.90 (6.169)	72	19.17 (5.965)	.47	
WDEQ^o^, mean (SD)		138	45.54 (16.738)	68	46.26 (17.622)	70	44.84 (15.928)	.62	

^a^ACG: attention control group.

^b^MTPG: mindfulness training during pregnancy group.

^c^Based on conversion of US $0.15=1 Chinese yuan at the time of writing.

^d^Participants rated the severity of early pregnancy reaction in the first 3 gestational months from 0 (the least serious) to 6 (the most serious).

^e^EPDS: Edinburgh Postnatal Depression Scale.

^f^PHQ-9: Patient Health Questionaire-9.

^g^GAD-7: Generalized Anxiety Disorder-7.

^h^PSS: Perceived Stress Scale.

^i^PA: Positive and Negative Affect Schedule-Positive Affect.

^j^NA: Positive and Negative Affect Schedule-Negative Affect.

^k^PSQI: Pittsburgh Sleep Quality Index.

^l^FSS: Fatigue Severity Scale.

^m^PM: Prospective and Retrospective Memory Questionnaire-Prospective Memory.

^n^RM: Prospective and Retrospective Memory Questionnaire-Retrospective Memory.

^o^WDEQ: Wijma Delivery Expectancy Questionnaire.

### Fidelity

Of the 84 participants allocated to the mindfulness training group, 10 of them did not activate the app, which was considered refusing the allocation. All of the remaining participants received the mindfulness training program. On the basis of the ITT sample, the mean number of completed training weeks was 3 weeks (SD 2.701). As a whole, 44/84 participants completed at least 4 weeks of training and the total completion rate was 52.4%. In all, 7/84 (8%) participants completed the entire 8-week training program.

### Overall Intervention Effect Between Groups

#### Primary Outcome: Depression

**Figure 3:**
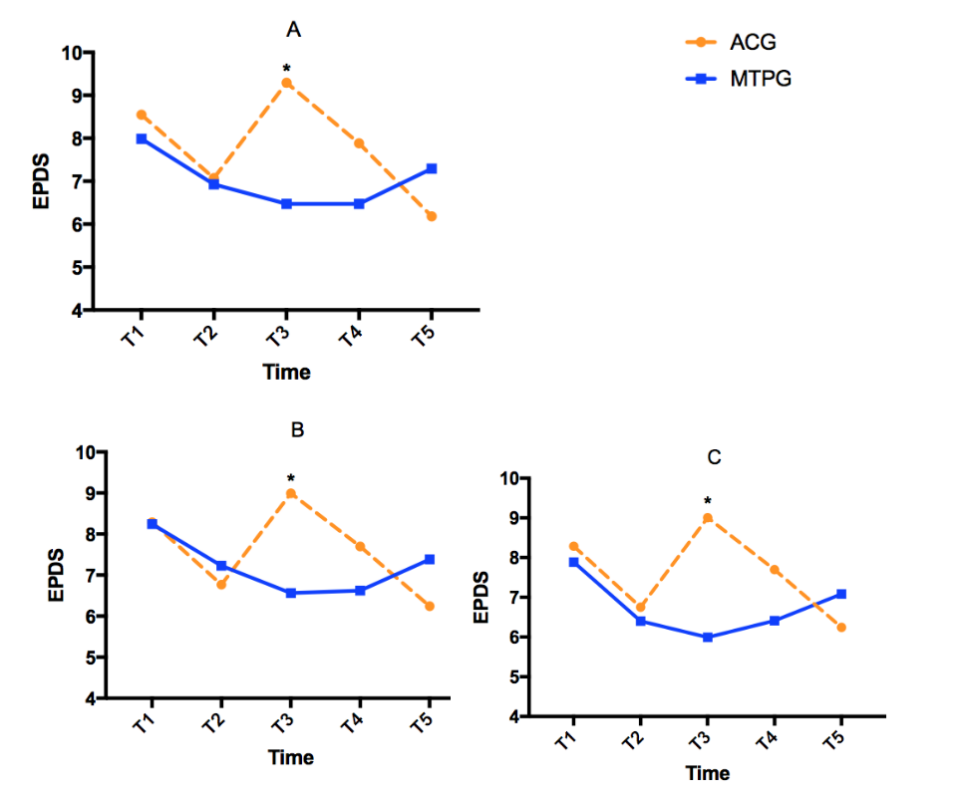
Overall intervention effect on Edinburgh Postnatal Depression Scale (EPDS) scores using the generalized estimated equation model in (A) intention-to-treat (ITT), (B) per-protocol (PP), and (C) per-protocol–intervention complete (PP-IC) samples. *significant between-group mean difference. ACG: attention control group; MTPG: mindfulness training in pregnancy group.

First, the overall test of the intervention effect on perinatal depression was performed through GEE analysis of the ITT sample. There was a significant time effect and group × time interaction effect on the change of the EPDS score (Table 3). As shown in Table 3 and Figure 3, the EPDS score at T2 decreased in both the mindfulness training and attention control groups. Thereafter, the EPDS score in the mindfulness training group continued to decline at T3, and remained at a low level at T4, but increased slightly postpartum. However, in the attention control group, the EPDS score increased markedly at T3 and then declined at T4 and T5. The mean difference between the two groups reached 2.82 points at T3. At T4, even though the between-group mean difference was not statistically significant, it was higher in the attention control group than in the mindfulness training group (Table 3). The between-group effect sizes for the EPDS were approximately medium at T3, as shown in Table 4. Analysis based on per-protocol and per-protocol–intervention complete samples showed the same results as the analysis from the ITT sample.

**Table 3:** Overall intervention effect on Edinburgh Postnatal Depression Scale scores using generalized estimated equation models.

Sample		Mean group difference (95% CI)	*P* value	Group effect		Time effect		Group × time effect	
				Wald chi-square (*df*=1)	*P* value	Wald chi-square (*df*=4)	*P* value	Wald chi-square (*df*=4)	*P* value
**ITT^a^**				1.4	.23	15.7	.003	16.2	.003
	T1^b^	0.56 (–0.72 to 1.84)	.39						
	T2^c^	0.14 (–1.65 to 1.94)	.88						
	T3^d^	2.82 (0.93 to 4.71)	.003						
	T4^e^	1.41 (–0.52 to 3.34)	.15						
	T5^f^	–1.11 (–3.00 to 0.79)	.25						
**PP^g^**				0.4	.54	13.8	.008	16.0	.003
	T1	0.04 (–1.22 to 1.30)	.95						
	T2	–0.46 (–2.24 to 1.32)	.61						
	T3	2.43 (0.58 to 4.28)	.01						
	T4	1.08 (–0.84 to 2.99)	.27						
	T5	–1.14 (–3.01 to 0.74)	.24						
**PP-IC^h^**				1.5	.23	12.6	.01	16.1	.003
	T1	0.40 (–1.04 to 1.84)	.59						
	T2	0.35 (–1.38 to 2.08)	.69						
	T3	3.02 (1.17 to 4.86)	.001						
	T4	1.28 (–0.92 to 3.49)	.23						
	T5	–0.84 (–3.02 to 1.34)	.45						

^a^ITT: intention-to-treat; n=84 mindfulness training group, n=84 attention control group.

^b^T1: baseline assessment.

^c^T2: 4 weeks after group allocation.

^d^T3: 8 weeks after group allocation.

^e^T4: 18 weeks after group allocation.

^f^T5: 6 weeks after delivery.

^g^PP: per-protocol; n=74 mindfulness training group, n=94 attention control group.

^h^PP-IC: per-protocol intervention completed; n=44 mindfulness training group, n=84 attention control group.

**Table 4:** Mean scores and effect sizes between and within groups for outcome measures.

Measure		ACG^a^			MTPG^b^			Between-group difference	
		Mean (SD)	Cohen *d*	*P* value	Mean (SD)	Cohen *d*	*P* value	Cohen *d*	95% CI
**EPDS^c^**									
	T1^d^	8.55 (4.593)	reference	N/A^e^	7.99 (3.873)	reference	N/A	0.13	–0.17 to 0.43
	T2^f^	7.63 (5.217)	0.19	.29	6.78 (4.816)	0.28	.11	0.17	–0.21 to 0.55
	T3^g^	9.09 (6.241)	–0.10	.58	6.49 (4.497)	0.36	.03	0.47	0.10 to 0.84
	T4^h^	8.02 (6.002)	0.10	.62	6.14 (4.552)	0.44	.01	0.35	–0.06 to 0.76
	T5^i^	6.25 (5.098)	0.47	.01	6.77 (4.693)	0.28	.12	–0.11	–0.52 to 0.31
**GAD-7^j^**									
	T1	4.99 (3.204)	reference	N/A	5.81 (3.243)	reference	N/A	–0.25	–0.58 to 0.07
	T2	4.23 (2.975)	0.25	.18	4.50 (3.720)	0.38	.03	–0.08	–0.46 to 0.30
	T3	5.56 (4.974)	–0.14	.46	4.46 (2.945)	0.44	.01	0.26	–0.10 to 0.63
	T4	5.93 (4.079)	–0.26	.17	4.32 (2.773)	0.49	.01	0.46	0.04 to 0.87
	T5	4.60 (3.967)	0.11	.58	4.32 (2.995)	0.48	.01	0.08	–0.33 to 0.49
**PSS^k^**									
	T1	5.33 (2.267)	reference	N/A	5.19 (2.374)	reference	N/A	0.06	–0.26 to 0.38
	T2	5.45 (2.403)	–0.05	.77	4.81 (2.782)	0.15	.41	0.25	–0.14 to 0.63
	T3	6.09 (3.630)	–0.25	.18	5.22 (2.726)	–0.01	.94	0.27	–0.10 to 0.63
	T4	6.09 (3.456)	–0.26	.20	5.24 (2.389)	–0.02	.92	0.29	–0.12 to 0.69
	T5	5.95 (2.087)	–0.28	.16	5.90 (2.234)	–0.31	.09	0.02	–0.39 to 0.43
**PA^l^**									
	T1	26.85 (5.255)	reference	N/A	27.72 (5.198)	reference	N/A	–0.17	–0.50 to 0.16
	T3	24.20 (7.105)	0.42	.03	27.37 (5.857)	0.06	.71	–0.49	–0.87 to –0.12
	T4	25.98 (7.220)	0.14	.50	28.26 (6.589)	–0.09	.62	–0.33	–0.75 to 0.08
	T5	28.05 (7.887)	–0.18	.39	26.94 (6.150)	0.14	.45	0.16	–0.25 to 0.57
**NA^m^**									
	T1	19.96 (5.111)	reference	N/A	21.29 (6.254)	reference	N/A	–0.23	–0.56 to 0.10
	T3	18.59 (6.703)	0.23	.20	18.65 (6.479)	0.41	.02	–0.01	–0.38 to 0.36
	T4	21.02 (7.333)	–0.17	.41	19.40 (5.852)	0.31	.09	0.24	–0.17 to 0.66
	T5	19.60 (7.493)	0.06	.77	18.48 (5.043)	0.49	.01	0.18	–0.23 to 0.59
**PSQI^n^**									
	T1	6.58 (2.577)	reference	N/A	7.13 (3.357)	reference	N/A	–0.18	–0.52 to 0.15
	T3	6.19 (2.514)	0.15	.41	6.33 (3.653)	0.23	.20	–0.04	–0.42 to 0.33
	T4	7.07 (2.640)	–0.19	.33	7.00 (3.156)	0.04	.83	0.02	–0.39 to 0.43
	T5	7.59 (3.768)	–0.31	.14	8.12 (3.585)	–0.29	.13	–0.14	–0.56 to 0.27
**FSS^o^**									
	T1	43.78 (8.899)	reference	N/A	42.55 (9.469)	reference	N/A	0.13	–0.19 to 0.46
	T3	40.57 (11.812)	0.31	.09	40.75 (9.657)	0.19	.28	–0.02	–0.39 to 0.35
	T4	43.21 (9.918)	0.06	.75	39.84 (9.678)	0.28	.12	0.34	–0.07 to 0.75
	T5	38.50 (11.073)	0.53	.01	38.23 (11.758)	0.40	.03	0.02	–0.39 to 0.44
**PM^p^**									
	T1	20.93 (6.106)	reference	N/A	20.36 (5.954)	reference	N/A	0.09	–0.23 to 0.42
	T3	20.34 (7.455)	0.09	.64	22.56 (6.995)	–0.34	.05	–0.31	–0.68 to 0.07
	T5	21.25 (8.755)	–0.04	.84	23.04 (7.914)	–0.38	.04	–0.21	–0.63 to 0.20
**RM^q^**									
	T1	19.90 (6.169)	reference	N/A	19.17 (5.965)	reference	N/A	0.12	–0.21 to 0.45
	T3	19.58 (7.399)	0.05	.80	21.19 (7.238)	–0.30	.08	–0.22	–0.59 to 0.15
	T5	21.00 (8.697)	–0.15	.48	22.40 (8.117)	–0.45	.02	–0.17	–0.58 to 0.25
**WDEQ^r^**									
	T1	46.26 (17.622)	reference	N/A	44.84 (15.928)	reference	N/A	0.08	–0.25 to 0.42
	T2	46.25 (19.312)	0.00	>.99	37.46 (21.055)	0.40	.03	0.44	0.05 to 0.82
	T3	49.43 (18.283)	–0.18	.34	44.10 (20.121)	0.04	.81	0.28	–0.10 to 0.65
	T4	48.42 (19.267)	–0.12	.55	46.04 (20.564)	–0.07	.73	0.12	–0.29 to 0.53

^a^ACG: attention control group.

^b^MTPG: mindfulness training during pregnancy group.

^c^EPDS: Edinburgh Postnatal Depression Scale.

^d^T1: baseline assessment.

^e^N/A: not applicable.

^f^T2: 4 weeks after group allocation.

^g^T3: 8 weeks after group allocation.

^h^T4: 18 weeks after group allocation.

^i^T5: 6 weeks after delivery.

^j^GAD-7: Generalized Anxiety Disorder-7.

^k^PSS: Perceived Stress Scale.

^l^PA: Positive and Negative Affect Schedule-Positive Affect.

^m^NA: Positive and Negative Affect Schedule-Negative Affect.

^n^PSQI: Pittsburgh Sleep Quality Index.

^o^FSS: Fatigue Severity Scale.

^p^PM: Prospective and Retrospective Memory Questionnaire-Prospective Memory.

^q^RM: Prospective and Retrospective Memory Questionnaire-Retrospective Memory.

^r^WDEQ: Wijma Delivery Expectancy Questionnaire.

#### Secondary Outcomes

Significant time and group × time interaction effects were found for anxiety in the ITT group (*χ*^2^_4_=18.8, *P*=.001; *χ*^2^_4_=13.1, *P*=.01) and per-protocol group (*χ*^2^_4_=19.6, *P*=.001; *χ*^2^_4_=13.3, *P*=.01) analyses, whereas only a significant time effect was found in the per-protocol–intervention complete group analysis (*χ*^2^_4_=21.7, P<.001). The anxiety score decreased at T2 and remained low in the mindfulness training group thereafter, but increased at T3 and T4 in the attention control group. A nearly medium between-group effect size was detected at T4 (Cohen *d*=0.46, 95% CI 0.04-0.87).

Significant group × time interaction effects were found for positive affect in the ITT (*χ*^2^_4_=8.4, *P*=.04), per-protocol (*χ*^2^_4_=8.2, *P*=.04), and per-protocol–intervention complete (*χ*^2^_4_=12.2 *P*=.007) analyses. The mindfulness training group maintained higher positive affect scores than the attention control group in the prenatal period, and reached a statistically significant mean difference at T3 for the ITT sample (–3.45, 95% CI –5.81 to –1.08, *P*=.004). The between-group effect size was medium (Cohen *d*=–0.49, 95% CI –0.87 to –0.12) at T3.

No significant between-group intervention effect was found on stress, log-transformed negative affect, log-transformed sleep, fatigue, log-transformed prospective memory, retrospective memory, and fear of childbirth. However, the between-group effect size on fear of childbirth was nearly medium (Cohen *d*=0.44, 95% CI 0.05-0.82) at T2. Details related to intervention effects on secondary outcomes are provided in Multimedia Appendix 5.

### Postintervention Depression Symptoms Remission

For the binary EPDS variable at T3, fewer participants reported positive depressive symptoms (EPDS>9) in the mindfulness training group than in the attention control group (15/63, 24% vs 24/54, 44%; *χ*^2^_1_=5.6, *P*=.02). Results of the logistic regression model indicated that mindfulness training led to a 0.609-times reduction on the risk of positive antenatal depressive symptoms compared to the attention control (Table 5) at postintervention. For EPDS reduction from T1 to T3, stepwise EPDS decreased scores ranging from 1 to 9 points were compared between groups; OR values remained statistically significant with EPDS score reduction>2. Mindfulness training showed a significant effect on EPDS score reduction over the attention control with the OR ranging from 3.471 to 27.986 (Table 5), indicating that mindfulness training had more potential to relieve depression symptoms than attention control.

**Table 5:** Intervention effect on depression symptoms remission at 8 weeks after allocation (T3) based on intention-to-treat analysis.

Depression symptom measure		ACG^a^ (n=54), n (%)	MTPG^b^ (n=63), n (%)	OR^c^ (95% confidence limit) (reference=ACG)^d^
**Positive depressive symptom (EPDS^e^ >9) at T3**				0.391 (0.164, 0.930)
	No	30 (56)	48 (76)	
	Yes	24 (44)	15 (24)	
**EPDS decrease≥9 from T1^f^ to T3**				N/A^g^
	Yes	0	7 (11)	
	No	54 (100)	56 (89)	
**EPDS decrease≥8 from T1 to T3**				16.391 (1.507, 178.297)
	Yes	1 (2)	10 (16)	
	No	53 (98)	53 (84)	
**EPDS decrease≥7 from T1 to T3**				17.982 (1.798, 179.868)
	Yes	1 (2)	12 (19)	
	No	53 (98)	51 (81)	
**EPDS decrease≥6 from T1 to T3**				27.986 (2.907, 269.468)
	Yes	1 (2)	15 (24)	
	No	53 (98)	48 (76)	
**EPDS decrease≥5 from T1 to T3**				7.687 (1.980, 29.846)
	Yes	5 (9)	18 (29)	
	No	49 (91)	45 (71)	
**EPDS decrease≥4 from T1 to T3**				4.295 (14.79, 12.473)
	Yes	9 (17)	24 (38)	
	No	45 (83)	39 (62)	
**EPDS decrease≥3 from T1 to T3**				3.471 (1.275, 9.448)
	Yes	14 (26)	26 (41)	
	No	40 (74)	37 (59)	
**EPDS decrease≥2 from T1 to T3**				1.938 (0.838, 4.481)
	Yes	20 (37)	31 (49)	
	No	34 (63)	32 (51)	
**EPDS decrease≥1 from T1 to T3**				1.708 (0.752, 3.876)
	Yes	24 (44)	35 (56)	
	No	30 (56)	28 (44)	

^a^ACG: attention control group.

^b^MTPG: mindfulness training during pregnancy group.

^c^OR: odds ratio.

^d^calculated from logistic regression models with depression remission at T3 as the dependent variable, adjusting for EPDS score at baseline and intended pregnancy.

^e^EPDS: Edinburgh Postnatal Depression Scale.

^f^T1: baseline assessment.

^g^N/A: not applicable.

### Subgroup Analysis

**Figure 4:**
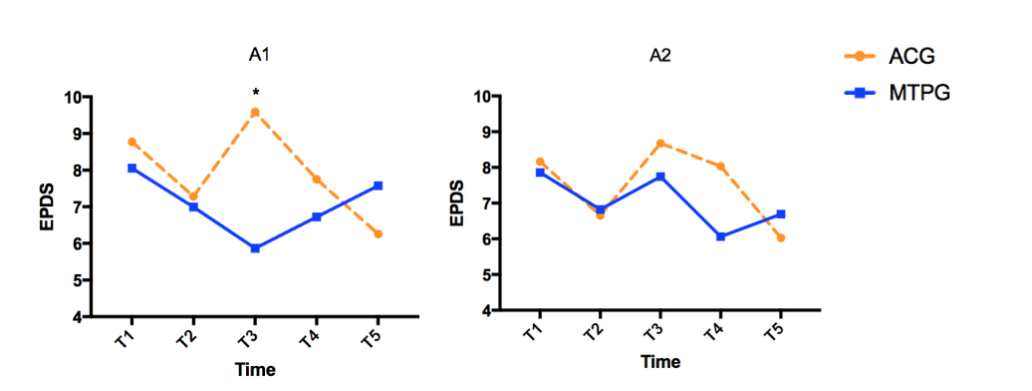
Overall intervention effect on Edinburgh Postnatal Depression Scale (EPDS) scores by parity. A1: Model in primipara; A2: Model in multipara. *significant between-group mean difference.

The mindfulness training vs attention control intervention effects on EPDS scores differed between a primipara and multipara state. In nulliparous women, the group × time interaction effect was statistically significant and the between-group mean difference reached 3.72 points on the EPDS at T3 (Table 6). However, no significant intervention effect was found in multiparous women (Table 6). Specifically, the EPDS score decreased at T3 in the mindfulness training group but increased in the attention control group in primipara, whereas it increased in both groups at T3 in multipara (Table 6 and Figure 4). However, the three-way interaction test (group × time × parity) did not reach statistical significance in the GEE model (*χ*^2^_4_=5.6, *P*=.24; see Multimedia Appendix 6). Results on subgroup analysis of anxiety and positive affect were consistent with those obtained for EPDS (see Multimedia Appendix 7).

**Table 6:** Overall intervention effect on Edinburgh Postnatal Depression Scale (EPDS) score by parity.

Parity status		Mean difference ACG^a^ – MTPG^b^ (95% CI)		*P* value		Group effect				Time effect					Group × time effect							
						Wald chi-square (*df*=1)		*P* value			Wald chi-square (*df*=4)		*P* value			Wald chi-square (*df*=4)		*P* value				
**Primipara** **(n=56 MTPG; n=53 ACG)**						1.3		.26			10.2		.04			18.1		.001				
	T1^c^		0.72 (–0.81 to 2.25)		.36																	
	T2^d^		0.29 (–1.85 to 2.44)		.79																	
	T3^e^		3.72 (1.38-6.06)		.002																	
	T4^f^		1.03 (–1.36 to 3.42)		.40																	
	T5^g^		–1.32 (–3.60 to 0.96)		.26																	
**Multipara** **(n=28 MTPG; n=31 ACG)**						0.2		.67			8.9		.06			2.4		.66				
	T1		0.30 (–1.98 to 2.59)		.79																	
	T2		–0.17 (–3.31 to 2.97)		.92																	
	T3		0.94 (–2.15 to 4.02)		.55																	
	T4		1.97 (–1.33 to 5.26)		.24																	
	T5		–0.67 (–4.05 to 2.72)		.70																	

^a^ACG: attention control group.

^b^MTPG: mindfulness training during pregnancy group.

^c^T1: baseline assessment.

^d^T2: 4 weeks after group allocation.

^e^T3: 8 weeks after group allocation.

^f^T4: 18 weeks after group allocation.

^g^T5: 6 weeks after delivery.

### Sensitivity Analysis

Sensitivity analysis in adjusted GEE models supported the primary results (Multimedia Appendix 8). In sensitivity analysis related to the number of follow ups (Multimedia Appendix 9), results from those who completed at least 2 or 3 follow ups showed a significant group × time interaction effect on the EPDS as well as in the primary analysis. However, mindfulness training vs attention control intervention effect trends were only found among participants who completed all follow ups. For anxiety, between-group mean differences at T3 reached statistical significance in participants who completed different numbers of follow ups in addition to the significant mindfulness training vs attention control intervention effect. Results from participants who completed at least 2 follow ups and all follow ups also supported the primary results on positive affect.

## Discussion

Our findings support the overall intervention effect of smartphone-based mindfulness training for the reduction of prenatal depression and anxiety symptoms, and enhancement of positive affect. Smartphone-based mindfulness training led to a lower rate of positive depression screening at postintervention and a greater decline in depression symptoms from baseline to postintervention relative to the attention control group. In addition, exploratory analysis suggested that parity is a potential moderator for smartphone-based mindfulness training among pregnant women despite not reaching statistical significance. The results of this study provide the first robust evidence on the effectiveness of self-help, smartphone-based mindfulness training for perinatal depression symptomology using an RCT design, relatively large sample size, and 32-week follow up through the postpartum period.

The effect of the intervention on depression as measured by the EPDS was substantial. Pregnant women who received mindfulness training had at least a 2.471-times higher odds of a decreased EPDS score from baseline to postintervention compared with women in the attention control group. At postintervention assessment, women in the mindfulness group experienced a 60.9% reduction in risk for positive depressive symptoms, with a medium between-group effect size on the EPDS. Studies assessing associations between mindfulness training and prenatal depression have not indicated clear agreement in between-group postintervention effects [3,44,49]. This study supports the positive effect of mindfulness, and the resulting medium effect size on depression is comparable with the effects of in-person mindfulness interventions [21,25]. The effect size was higher than that reported in nonpregnant women (a small effect on depression, Hedges *g*=0.29) [18], revealing that smartphone-based mindfulness training is especially suitable for pregnant women, consistent with previous studies assessing pregnant women’s preference for internet-based services [13,14]. Few studies of internet or smartphone-based mindfulness interventions in pregnancy have appeared in the literature, and those that are available mainly report preliminary effects on maternal depressive symptoms [27-29]. Thus, our study has provided the first robust evidence from an RCT in this area.

The sustained low levels of depressive symptoms for pregnant women who received mindfulness training are also encouraging. Even though no difference was found at the midpoint of the intervention, the significant between-group difference after the intervention was consistent with findings from previous in-person mindfulness studies during pregnancy [50-53]. This study found a longer-term effect of online mindfulness training in participants in contrast to other recent research that was more limited by the high rate of dropout [28]. Our results are also consistent with another study assessing depressive symptoms at each session of eight MBCT sessions, showing larger decreases of depression severity in the last phase of the intervention [54]. This suggests that the dose of an intervention is a promising factor potentially affecting the intervention effect. A further issue noted in this study is that the trend of a low level of depressive symptoms in the mindfulness group did not sustain throughout the postpartum stage. Previous studies of prenatal mindfulness interventions are also conflicting in this regard [30,53,55,56]. However, considering the growing audience for smartphone-based intervention formats, and the potential to reach larger audiences, the platform is quite advantageous. The treatment gap in the postpartum period may be further reduced through smartphone-based interventions, which can include reminders and functions to enhance frequency and compliance.

The exploration of additional benefits of mindfulness training in pregnancy provided promising results of a low anxiety level and high positive affect in follow ups. Mental health problems in pregnant women may be experienced as a cluster of symptoms during the perinatal period. In empirical research, multiple symptoms such as anxiety, stress, and fatigue were found to be correlated with perinatal depression [57-59]. Traditional perspectives on psychopathology presumed that some co-occurring symptoms originate from an underlying common cause [60,61]. Although determining the root causes of perinatal depression and secondary outcomes was outside the scope of this study, the findings of concurrent improvement on depression symptoms, anxiety symptoms, and positive affect utilizing mindfulness training nonetheless provide support for this approach. Consistent with other studies on mindfulness interventions [21,44,52], anxiety symptoms in this study were improved as well as depression symptoms. Another notable finding is the maintenance of positive affect in follow up, in contrast to less consistent findings on the effect of a mindfulness intervention on positive affect [21]. Duncan and Bardacke [19] reported a within-subject increase of positive affect in pregnant women through a pre-post test design trial. The between-group comparison and longer-term follow up in this study suggested that mindfulness training was helpful for pregnant women to maintain their initial positive affect, but did not constitute a pattern of increase. Mindfulness seems to particularly help pregnant women to maintain awareness of positive affect during the challenging perinatal period. Thus, mindfulness training in pregnancy may be a potentially cost-effective measure to resolve issues such as those included as secondary outcomes in this study. Cost-effectiveness in addressing multidimensional perinatal mental health is particularly appropriate for LAMICs. However, the full extent of costs involved in the intervention need to be specifically studied in future research.

Understanding the individual characteristics that may affect the efficacy of an intervention is key for evaluating suitability and usability. In this study, we tried to ascertain the moderating effect of parity. However, the nonsignificance of the three-way interaction test suggests that parity is only one potential factor influencing the intervention effect. Mindfulness interventions were previously reported to be less accessible with high loss to follow up among women who already had children [21]. Lack of time for participation and existing family commitments are important reasons for the likelihood that women may drop out of longitudinal research [62]. Reduction of depression symptoms among primipara who received mindfulness training was found only at postintervention in this study, pointing out a future direction for investigating the moderating effect over the shorter or longer term. Further studies are still needed for subgroups and vulnerable populations receiving this type of intervention.

This study expands on existing studies of mindfulness training in pregnancy in several key areas. First, it shows that prenatal mindfulness training can be extended through smartphone-based delivery, which may lead to reductions in demand on both therapists and service costs [63]. The cost savings would be especially helpful for LAMICs. Second, the demonstrated longer-term effect throughout pregnancy, and the multiple effects on depression, anxiety, and positive affect are beneficial to simplify intervention protocols. Third, the subgroup analysis on parity points to the importance for parous women to receive mental health services in the perinatal period. Additionally, the RCT design with attention control and sensitivity analysis reduced risk of bias and provided rigorous findings.

Several limitations should be noted. First, the potential impact of the dropout rate must be acknowledged. In this study, the overall rate of dropout was 34.5%, which is slightly higher than that reported for a recent in-person antenatal mindfulness program with a 28% overall dropout rate [20]. Difficulties with retention are a constant problem for self-help online research [28]. However, we also noted that the dropout rate at postintervention for this study was 22.0%, which is not larger than the estimated 30% attrition accounted for when calculating sample size, and did not limit the statistical power to test the interaction effect and immediate postintervention effect. The completion rate of the intervention is another limitation as it was unsatisfactory. Only 7 of 84 participants in the mindfulness group completed the entire 8-week training, and 44/84 participants completed the determined completion standard with a completion rate of 52.4%. Reported completion rates of interventions vary among online interventions, ranging from 21% to 74% [28,64] depending on definitions of completion rate and the intervention format. Even though the completion rate herein is within that range, misinterpretation of the intervention effect is a risk of bias from nonuse of the intervention. Third, 10 of 84 participants in the intervention group did not activate the mindfulness training app even after they agreed to participate in this program and were reminded by the research assistant. The high proportion of inactive participants in the intervention group weakens the strength of the RCT design. Fourth, the longer-term effect and the subgroup analysis related to parity were exploratory analyses in this study, and the sample sizes may be insufficient for comparing specific differences at T4, T5, and between subgroups. Future studies that focus on the longer-term effect or subgroups are suggested to calculate sample sizes based on the time point or group that had the lowest effect size. Participants in this study were able to infer their allocation and were unblinded. The multiple assessment of outcomes increases the risk for a type I error. In addition, the broad confidence intervals indicate the need for caution in interpreting the finding of depression remission at postintervention for generalizing the result. The sample included in this study was recruited in a single hospital, further limiting the generalizability of our findings. It is important for further researchers to conduct multicenter studies to examine these findings. Finally, even though China lags behind high-income countries in the widespread provision of perinatal mental health services, it is considered an upper-middle-income country and is in rapid development. The use of smartphones and networks in China may be much better than that in other LAMICs, especially low-income countries. More studies focusing on convenient and low-cost perinatal psychological interventions in these countries are still needed.

## Appendix

Multimedia Appendix 1 Mindfulness training during pregnancy (MTP) program construction process.

Multimedia Appendix 2 Timepoints of outcome indicators assessed.

Multimedia Appendix 3 Statistical analysis.

Multimedia Appendix 4 Analysis of dropouts.

Multimedia Appendix 5 Overall intervention effects on secondary outcomes.

Multimedia Appendix 6 GEE model of subgroup analysis.

Multimedia Appendix 7 Subgroup analysis on secondary outcomes.

Multimedia Appendix 8 Sensitivity analysis - adjusted model.

Multimedia Appendix 9 Sensitivity analysis for participants who completed different numbers of follow ups.

Multimedia Appendix 10 CONSORT-eHEALTH checklist (V 1.6.1).

## Abbreviations

EPDS: Edinburgh Postnatal Depression Scale
GEE: generalized estimating equations
ITT: intention to treat
LAMIC: low- and middle-income country
MBCT: Mindfulness Behavioral Cognitive Therapy
MBI: mindfulness-based intervention
OR: odds ratio
PHQ-9: Patient Health Questionnaire-9
RCT: randomized controlled trial
T1: baseline
T2: 4 weeks after allocation
T3: 8 weeks after allocation
T4: 18 weeks after allocation
T5: 6 weeks after delivery
