# Supplementary material for: Effectiveness of Smartphone-Based Mindfulness Training on Maternal Perinatal Depression: Randomized Controlled Trial
Source: J Med Internet Res. 2021 Jan 27;23(1):e23410. doi: 10.2196/23410 (PMC7875700; doi:10.2196/23410)
Supplement: Multimedia Appendix 1 [file jmir_v23i1e23410_app1.doc]

# **MTP construction process**

The Mindfulness Training during Pregnancy (MTP) used in this study was adapted from the classic Mindfulness Behavioral Cognitive Therapy (MBCT) course developed by John Teasdale, Mark Williams and Zindel Segal. MBCT combines thoughts of cognitive behavioral therapy and meditative practices based on mindfulness, and is originally designed to help people who suffered from recurrent depressive episodes. Nowadays, MBCT has been adapted to a range of different populations and contexts [1]. MBCT emphases the “being” mode of human mind possesses for receiving and processing data other than “doing” mode. It helps participants learn to recognize their sense of being and see themselves as separate from their thoughts and moods, so that to get rid of depression spiral. Classic MBCT is an 8-week program with eight weekly classes, 2.5 hours each, and daily home practice assignments for around 45 minutes each day.

“*The mindful way workbook: An 8-Week Program to Free Yourself Depression and Emotional Distress*” [2] written by the above three authors was used as the primary guidebook and the Chinese version translated by Jing Nie was referred in the current study. A psychologist with 5-years of mindfulness experience led the adaption of the MTP course. One obstetrician, one obstetrics nurse, and two research assistants with mindfulness experience participated. Three principles guided the adaption process: focus on perinatal depression and negative emotions, make physical adaptions for pregnant women, and simplify and shorten the practice properly with each formal training limited in 25 minutes. Some poses, and mindfulness techniques, were adapted or omitted for intervention in this population, in particular mindful stretching and body scan were adapted. Firstly, any poses that were considered to increase abdominal pressure, such as forward bending pose and airplane pose, were removed, as advised by the study’s obstetrician. Simple stretch poses appropriate to pregnant bodies were utilized instead. Secondly, mindful childbirth poses were added with the help of the obstetric nurse. Third, a particular component - “Week 7: Mindful pregnancy and childbirth” - was developed in the MTP where body scan specific for the pregnant body and mindfulness of fetal movement were included.

The MTP was delivered through *Spirits Healing* application, which was a custom-built mobile application by a third-party company. The application was developed in Chinese and was available in both Android and iOS operating systems in mainland China. There’re two parts in the application, “MTP course” and “My information”, providing reading materials, recordings for guided practice, and videos, and personal information and task records separately. Participants were able to navigate contents and write mindfulness journal in the app. The MTP training automatically updated every day and participants practiced according to their own schedules. The application was debugged three times during the trial due to adaptation of phone systems, but no changes related to intervention content were made. For safety and de-stigmatization, participants were reminded that this application is not equivalent psychotherapy and referred to professional support when necessary.

References:

1. Alsubaie, M., et al., *Mechanisms of action in mindfulness-based cognitive therapy (MBCT) and mindfulness-based stress reduction (MBSR) in people with physical and/or psychological conditions: A systematic review.* Clin Psychol Rev, 2017. **55**: p. 74-91.

2. Teasdale, J.D., J.M.G. Williams, and Z.V. Segal, *The mindful way workbook: An 8-week program to free yourself from depression and emotional distress*. 2014: Guilford Publications.
