# Supplementary material for: Effectiveness of Smartphone-Based Mindfulness Training on Maternal Perinatal Depression: Randomized Controlled Trial
Source: J Med Internet Res. 2021 Jan 27;23(1):e23410. doi: 10.2196/23410 (PMC7875700; doi:10.2196/23410)
Supplement: Multimedia Appendix 2 [file jmir_v23i1e23410_app2.doc]

# **Timepoints of outcome indicators assessed**

Table S1. Timepoints of outcome indicators assessed.

|  | **T1** | **T2** | **T3** | **T4** | **T5** |
| --- | --- | --- | --- | --- | --- |
| EPDS | √ | √ | √ | √ | √ |
| GAD-7 | √ | √ | √ | √ | √ |
| PSS | √ | √ | √ | √ | √ |
| PANAS | √ | - | √ | √ | √ |
| PSQI | √ | - | √ | √ | √ |
| FSS | √ | - | √ | √ | √ |
| PRMQ | √ | - | √ | - | √ |
| WDEQ | √ | √ | √ | √ | - |

*Note:* EPDS = Edinburgh Postnatal Depression Scale; GAD-7 = Generalized Anxiety Disorder scale; PSS = Perceived Stress Scale; PANAS = Positive and Negative Affect Schedule; PSQI = Pittsburgh Sleep Quality Index; FSS = Fatigue Severity Scale; PRMQ = Prospective and Retrospective Memory Questionnaire; WDEQ = Wijma Delivery Expectancy Questionnaire.
