# Supplementary material for: Effectiveness of Smartphone-Based Mindfulness Training on Maternal Perinatal Depression: Randomized Controlled Trial
Source: J Med Internet Res. 2021 Jan 27;23(1):e23410. doi: 10.2196/23410 (PMC7875700; doi:10.2196/23410)
Supplement: Multimedia Appendix 3 [file jmir_v23i1e23410_app3.doc]

**Statistical analysis**

*Primary analysis*

Statistical analyses were performed with SPSS 23.0. The primary analysis used intention-to-treat (ITT) approach. Normality of outcomes at baseline were visually examined and it resulted that EPDS, PSS, PA, FSS and WDEQ were normally distributed, GAD-7 and RM were nearly normally distributed, and NA, PSQI and PM were found to have non-normal data distribution. Thus, NA, PSQI and PM were transformed by log. No more than five individuals for each measure at T1 had single entry missing, which were considered as missing at random and were imputed with median of item. For T2 and above, the web-based survey included rules that eliminated missing items on questionnaires. So, no item missed at follow-up due to the requirement of web-based survey. Independent sample *t* test and *chi*-square test were used to compare the baseline characteristics between intervention group and control group.

Comparison between study sample and drop-out sample characteristics at baseline were conducted using independent sample *t* test and *chi*-square test. And a logistic regression model with binary drop-out status as dependent variable was performed to identify factors influencing drop-out. Potential influencing factors recommended by previous studies [1, 2] were included: randomization group, baseline EPDS score, age, gestational days at baseline, BMI before pregnancy, education years, work status, family monthly incomes, primiparity, history of abortion, history of induced labor, history of embryo damage, history of previous disease and intended pregnancy.

To assess the population-averaged MTPG vs. ACG intervention effect on outcomes, Generalized Estimating Equations (GEE) were performed. This approach has been recommended because it is able to handle missing data appropriately and can keep stable in different correlation matrix [3]. The multilevel data has two levels: assessments as Level 1 nested within participants as Level 2. In GEE, the participants and assessment timepoints were designated as subject variables and within-subject variable, respectively, with exchangeable working correlation matrix and full maximum likelihood estimation applied. The continuous outcome variables in five timepoints were the dependent variable. Main effects of group, time, and Group × Time interaction effect were examined. Testing of simple main effect for group was also explored by examining differences between groups at each time point.

To further assess the intervention effect on depression remission at post-intervention (T3), binary positive depressive symptom at T3 and EPDS reductions from T1 to T3 were compared between intervention and control groups. Randomization group variable (ACG = 0, MTPG = 1) was included into a binary logistic regression model with positive depressive symptom (“EPDS < 10” = 0, “EPDS ≥ 10” = 1) at T3 as dependent variable, adjusting for EPDS score at baseline and between-group imbalanced factor (intended pregnancy) after randomization. A series of between-group odd ratios (ORs) of depression remission, in EPDS reduction ranging from 1 to 9, to simulate the intervention effect on depression remission, were also performed in logistic regression models adjusting for EPDS score at baseline and intended pregnancy, due to lacking of consistent minimal clinically important difference of EPDS.

Effect sizes are presented as Cohen’s *d* based on ITT rule. Cohen’s *d* between groups and follow-ups referred to baseline data were calculated. Effect sizes were labelled as small (*d* = 0.2), medium (*d* = 0.5) and large (*d* = 0.8). A two-sided *P* value of less than 0.05 was considered statistically significant.

*Subgroup analysis*

As previous studies referred [4, 5], nulliparous women showed different trends in pre- and postnatal depression compared with multiparous women. So, subgroup analysis by parity (primipara/multipara) were conducted on all outcome indicators to test whether parity affected MTPG vs. ACG intervention effect. Besides main effects, two-way interactions (Group × Time, Group × Parity, Time × Parity) and three-way interaction (Group × Time × Parity) were also included in GEE models. In the subgroup analysis, *P* value of less than 0.025 was considered statistically significant after *Bonferroni* multiple-corrected.

*Sensitivity analysis*

Several sensitivity analyses were conducted to evaluate the robustness of the intervention effect, according to an approach for sensitivity analyses in clinical trials [6].

Firstly, the between-group differences were analyzed in multiple groups as the CONSORT guidelines recommended [7]: (1) ITT group, which included all randomized participants, regardless of whether they received allocation as planned or not; (2) per-protocol (PP) group, in which participants were allocated according to their real actions, for instance, one participant who had been randomized into MTPG did not activate MTPG task, then he/she would be allocated into ACG; (3) as-treated (AT) group, including subjects with the treatment regimen that they received (referred as the PP-IC group; IC: “intervention completed”). Only a sample of intervention participants who completed the training were included in intervention group, and those who did not activate MTPG intervention were included in control group as the allocated ACG sample. The criteria to define “complete the training” was mentioned in “Intervention fidelity” session, and participants in intervention group of PP-IC were considered to be adherent to the program.

Secondly, adjusted GEE models with baseline imbalanced factor (intended pregnancy) were also performed.

Thirdly, considering the estimation bias caused by lost to follow-up, intervention effect based on GEE were also conducted in participants who completed different number of follow-ups.

**References**

1. Karyotaki, E., et al., *Predictors of treatment dropout in self-guided web-based interventions for depression: an ‘individual patient data’meta-analysis.* 2015. **45**(13): p. 2717-2726.

2. Klein, J.P., et al., *Effects of a Psychological Internet Intervention in the Treatment of Mild to Moderate Depressive Symptoms: Results of the EVIDENT Study, a Randomized Controlled Trial.* Psychotherapy and Psychosomatics, 2016. **85**(4): p. 218-228.

3. Wang, M.J.A.i.S., *Generalized estimating equations in longitudinal data analysis: a review and recent developments.* 2014. **2014**.

4. Takehara, K., et al., *Prevalence trends of pre- and postnatal depression in Japanese women: A population-based longitudinal study.* Journal of Affective Disorders, 2018. **225**: p. 389-394.

5. Tsuchida, A., et al., *Changes in the association between postpartum depression and mother-infant bonding by parity: Longitudinal results from the Japan Environment and Children's Study.* Journal of Psychiatric Research, 2019. **110**: p. 110-116.

6. Thabane, L., et al., *A tutorial on sensitivity analyses in clinical trials: the what, why, when and how.* BMC Medical Research Methodology, 2013. **13**(1): p. 92.

7. Eysenbach, G. and C.-E.G.J.J.o.m.I. research, *CONSORT-EHEALTH: improving and standardizing evaluation reports of Web-based and mobile health interventions.* 2011. **13**(4): p. e126.
