# Supplementary material for: Effectiveness of Smartphone-Based Mindfulness Training on Maternal Perinatal Depression: Randomized Controlled Trial
Source: J Med Internet Res. 2021 Jan 27;23(1):e23410. doi: 10.2196/23410 (PMC7875700; doi:10.2196/23410)
Supplement: Multimedia Appendix 4 [file jmir_v23i1e23410_app4.doc]

**Analysis on drop-out**

Table S2. Comparison between drop-out sample and study sample.

|  | **In group (n = 110)** | | **Drop-out (n = 58)** | | **Statistics** | **P** |
| --- | --- | --- | --- | --- | --- | --- |
|  | **n** | **M ± SD or F (percentage)** | **n** | **M ± SD or F (percentage)** |
| Group | 110 |  | 58 |  | χ^2^=2.633 | .105 |
| ACG |  | 50 (45.5%) |  | 34 (58.6%) |  |  |
| MTPG |  | 60 (54.5%) |  | 24 (41.4%) |  |  |
| EPDS at baseline | 110 | 8.01 ± 4.287 | 58 | 8.76 ± 4.16 | T=-1.089 | .278 |
| Negative |  | 68 (61.8%) |  | 32 (55.2%) | χ^2^=0.696 | .404 |
| Positive |  | 42 (38.2%) |  | 26 (44.8%) |  |  |
| Age | 110 | 29.81 ± 4.074 | 58 | 30.10 ± 3.928 | T=-0.451 | .653 |
| 18~34 |  | 93 (84.5%) |  | 47 (81.0%) | χ^2^=0.337 | .562 |
| 35 ~ |  | 17 (15.5%) |  | 11 (19.0%) |  |  |
| Gestational days at baseline | 110 | 96.43 ± 13.836 | 58 | 103.33 ± 15.414 | T=-2.954 | **.004** |
| BMI before pregnancy | 107 | 21.93 ± 2.906 | 55 | 21.58 ± 3.438 | T=0.681 | .497 |
| Education years | 109 | 15.66 ± 2.389 | 56 | 14.96 ± 2.256 | T=1.806 | .073 |
| Work status | 108 |  | 55 |  | χ^2^=1.550 | .213 |
| Unemployed |  | 22 (20.4%) |  | 16 (29.1%) |  |  |
| Employed |  | 86 (79.6%) |  | 39 (70.9%) |  |  |
| Family monthly income (*yuan*) | 100 |  | 52 |  | Fisher=4.591 | .169 |
| <2000 |  | 0 |  | 1 (1.9%) |  |  |
| 2000~4000 |  | 22 (22.0%) |  | 10 (19.2%) |  |  |
| 4000~6000 |  | 26 (26.0%) |  | 20 (38.5%) |  |  |
| ≥6000 |  | 52 (52.0%) |  | 21 (40.4%) |  |  |
| Primiparity | 110 |  | 58 |  | χ^2^=0.046 | .830 |
| Yes |  | 38 (34.5%) |  | 21 (36.2%) |  |  |
| No |  | 72 (65.5%) |  | 37 (63.8%) |  |  |
| History of abortion | 110 |  | 58 |  | χ^2^=0.032 | .858 |
| Yes |  | 61 (55.5%) |  | 33 (56.9%) |  |  |
| No |  | 49 (44.5%) |  | 25 (43.1%) |  |  |
| History of induced labor | 110 |  | 58 |  | fisher | 1.000 |
| Yes |  | 104 (94.5%) |  | 55 (94.8%) |  |  |
| No |  | 6 (5.5%) |  | 3 (5.2%) |  |  |
| History of embryo damage | 110 |  | 58 |  | χ^2^=0.431 | .512 |
| Yes |  | 90 (81.8%) |  | 45 (77.6%) |  |  |
| No |  | 20 (18.2%) |  | 13 (22.4%) |  |  |
| Intended pregnancy | 106 |  | 57 |  | χ^2^=0.135 | .713 |
| Yes |  | 81 (76.4%) |  | 45 (78.9%) |  |  |
| No |  | 25 (23.6%) |  | 12 (21.1%) |  |  |
| History of previous disease | 106 |  | 55 |  | fisher | 1.000 |
| Yes |  | 99 (93.4%) |  | 52 (94.5%) |  |  |
| No |  | 7 (6.6%) |  | 3 (5.5%) |  |  |

**Table S3. Logistic regression with drop-out status as dependent variable.**

|  | **B** | **SE** | **Ward** | **P value** | **OR** | **LICL** | **HICL** |
| --- | --- | --- | --- | --- | --- | --- | --- |
| Group (R: ACG) | -.571 | .439 | 1.696 | .193 | .565 | .239 | 1.334 |
| EPDS at baseline | -.019 | .049 | .147 | .701 | .982 | .892 | 1.080 |
| Advanced age (R: No) | 1.113 | .668 | 2.774 | .096 | 3.043 | .821 | 11.273 |
| Gestational days at baseline | .032 | .015 | 4.889 | **.027** | **1.033** | **1.004** | **1.063** |
| BMI before pregnancy | -.067 | .074 | .834 | .361 | .935 | .809 | 1.080 |
| Education years | -.212 | .111 | 3.633 | .057 | .809 | .650 | 1.006 |
| Work status (R: No) | -.147 | .560 | .069 | .792 | .863 | .288 | 2.585 |
| Family monthly income */ yua*n |  |  | .653 | .884 |  |  |  |
| >6000 | reference |  |  |  |  |  |  |
| 4000~6000 | 22.600 | 40192.969 | .000 | 1.000 | 6.532 | .000 | . |
| 2000~4000 | .278 | .590 | .221 | .638 | 1.320 | .415 | 4.197 |
| 0~2000 | .400 | .508 | .622 | .430 | 1.492 | .552 | 4.035 |
| Primiparity (R: No) | .356 | .557 | .409 | .523 | 1.428 | .479 | 4.254 |
| History of abortion (R: No) | -.845 | .537 | 2.473 | .116 | .430 | .150 | 1.231 |
| History of induced labor (R: No) | -.581 | 1.007 | .333 | .564 | .559 | .078 | 4.028 |
| History of embryo damage (R: No) | .477 | .683 | .488 | .485 | 1.612 | .422 | 6.149 |
| History of previous disease (R: No) | -.296 | .819 | .131 | .718 | .744 | .149 | 3.704 |
| Intended pregnancy (R: No) | -.576 | .556 | 1.071 | .301 | .562 | .189 | 1.673 |
