# Supplementary material for: Effectiveness of Smartphone-Based Mindfulness Training on Maternal Perinatal Depression: Randomized Controlled Trial
Source: J Med Internet Res. 2021 Jan 27;23(1):e23410. doi: 10.2196/23410 (PMC7875700; doi:10.2196/23410)
Supplement: Multimedia Appendix 5 [file jmir_v23i1e23410_app5.doc]

# **Overall intervention effects on secondary outcomes**

1. **GAD-7**

**Table S4. Longer-term effect intervention effect on GAD-7 based on raw GEE model**.

|  |  | **MD _ACG-MTPG_** | ***p* value** | **Group effect** | | **Time effect** | | **Group × Time effect** | |
| --- | --- | --- | --- | --- | --- | --- | --- | --- | --- |
|  |  |  |  | **Wald *χ*^2^** | ***p* value** | **Wald *χ*^2^** | ***p* value** | **Wald *χ*^2^** | ***p* value** |
| **ITT (n=166)** | T1 | -0.91 (-1.93, 0.10) | 0.078 | 0.187 | 0.666 | 18.801 | **0.001** | 13.097 | **0.011** |
|  | T2 | -0.38 (-1.56, 0.81) | 0.533 |  |  |  |  |  |  |
|  | T3 | 1.25 (-0.22, 2.73) | 0.096 |  |  |  |  |  |  |
|  | T4 | 1.19 (-0.15, 2.53) | 0.083 |  |  |  |  |  |  |
|  | T5 | -0.23 (-1.63, 1.16) | 0.745 |  |  |  |  |  |  |
| **PP (n=166)** | T1 | **-1.22 (-2.24, -0.19)** | **0.020** | 0.081 | 0.776 | 19.601 | **0.001** | 13.270 | **0.010** |
|  | T2 | -0.76 (-1.96, 0.44) | 0.213 |  |  |  |  |  |  |
|  | T3 | 0.92 (-0.50, 2.33) | 0.204 |  |  |  |  |  |  |
|  | T4 | 0.92 (-0.41, 2.25) | 0.174 |  |  |  |  |  |  |
|  | T5 | -0.45 (-1.80, 0.90) | 0.512 |  |  |  |  |  |  |
| **PP-IC (n=136)** | T1 | -0.76 (-1.94, 0.43) | 0.211 | 0.130 | 0.719 | 21.717 | **<0.001** | 7.637 | 0.106 |
|  | T2 | -0.27 (-1.37, 0.83) | 0.633 |  |  |  |  |  |  |
|  | T3 | 0.89 (-0.62, 2.40) | 0.250 |  |  |  |  |  |  |
|  | T4 | 0.98 (-0.41, 2.37) | 0.168 |  |  |  |  |  |  |
|  | T5 | -0.02 (-1.51, 1.47) | 0.977 |  |  |  |  |  |  |


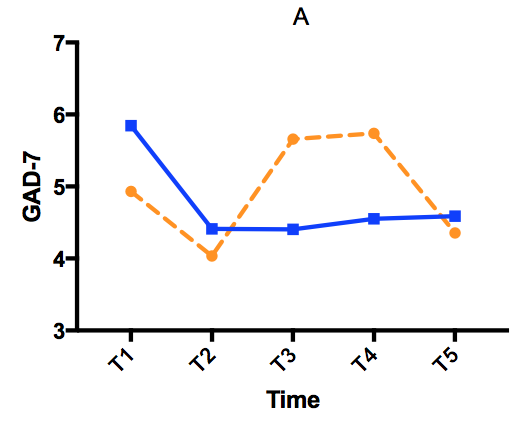

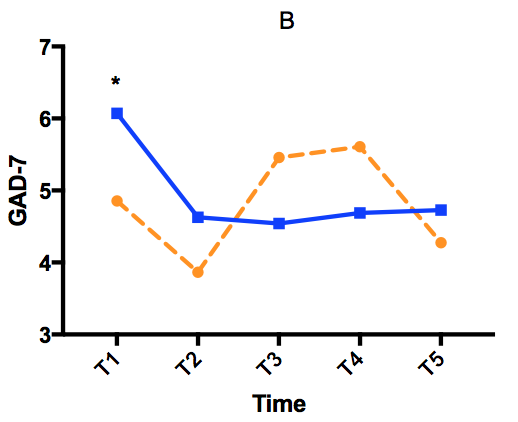

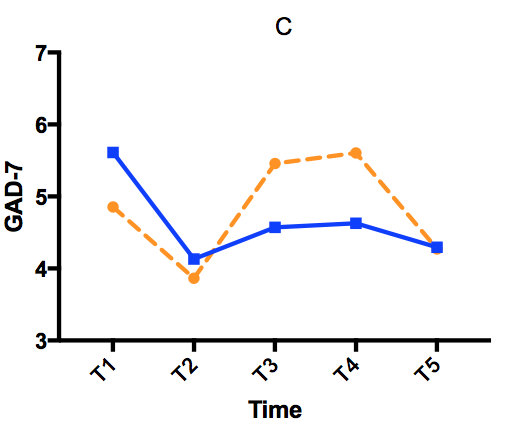

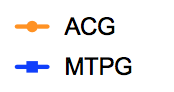


**Figure S1. Longer-term intervention effect on GAD-7 based on raw GEE model**

Note. A. based on ITT; B. based on PP; C. based on PP-IC; * means significant between-group mean difference.

1. **PSS**

**Table S5. Longer-term effect intervention effect on PSS** based on raw GEE model.

|  |  | **MD _ACG-MTPG_** | ***p* value** | **Group effect** | | **Time effect** | | **Group × Time effect** | |
| --- | --- | --- | --- | --- | --- | --- | --- | --- | --- |
|  |  |  |  | **Wald *χ*^2^** | ***p* value** | **Wald *χ*^2^** | ***p* value** | **Wald *χ*^2^** | ***p* value** |
| **ITT (n=166)** | T1 | 0.08 (-0.66, 0.81) | 0.835 | 1.667 | 0.197 | 11.034 | **0.026** | 4.162 | 0.384 |
|  | T2 | 0.37 (-0.58, 1.32) | 0.445 |  |  |  |  |  |  |
|  | T3 | 0.97 (-0.17, 2.11) | 0.095 |  |  |  |  |  |  |
|  | T4 | 0.72 (-0.46, 1.89) | 0.231 |  |  |  |  |  |  |
|  | T5 | -0.06 (-0.91, 0.78) | 0.884 |  |  |  |  |  |  |
| **PP (n=166)** | T1 | 0.16 (-0.59, 0.90) | 0.680 | 0.927 | 0.336 | 11.174 | **0.025** | 2.208 | 0.697 |
|  | T2 | 0.09 (-0.87, 1.05) | 0.859 |  |  |  |  |  |  |
|  | T3 | 0.74 (-0.37, 1.86) | 0.190 |  |  |  |  |  |  |
|  | T4 | 0.57 (-0.59, 1.74) | 0.336 |  |  |  |  |  |  |
|  | T5 | -0.01 (-0.86, 0.83) | 0.979 |  |  |  |  |  |  |
| **PP-IC (n=136)** | T1 | 0.40 (-0.37, 1.16) | 0.310 | 0.984 | 0.321 | 12.652 | **0.013** | 2.028 | 0.731 |
|  | T2 | 0.12 (-0.87, 1.11) | 0.812 |  |  |  |  |  |  |
|  | T3 | 0.68 (-0.51, 1.87) | 0.263 |  |  |  |  |  |  |
|  | T4 | 0.65 (-0.62, 1.92) | 0.317 |  |  |  |  |  |  |
|  | T5 | -0.04 (-1.04, 0.97) | 0.946 |  |  |  |  |  |  |


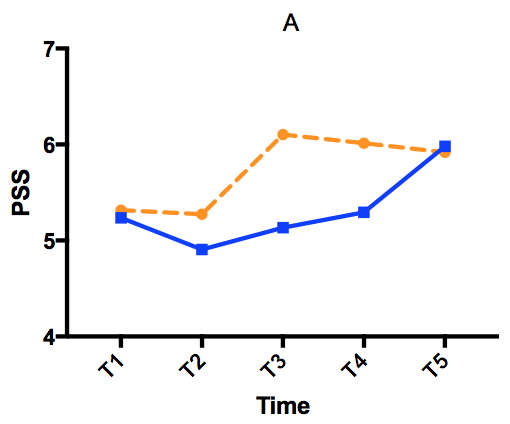

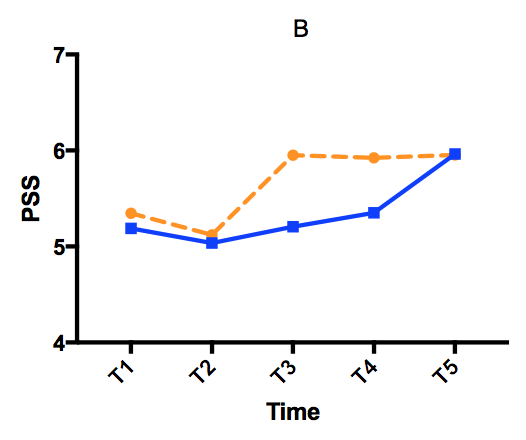

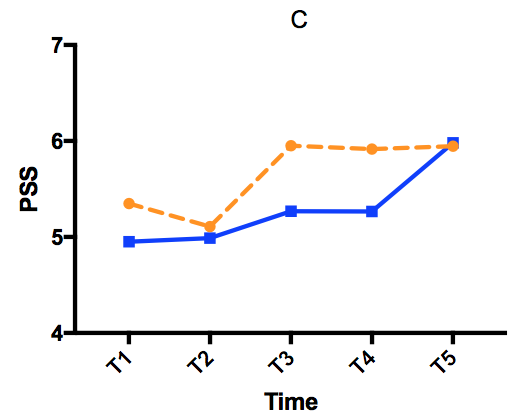

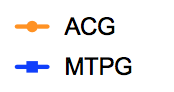


**Figure S2. Longer-term intervention effect on PSS based on raw GEE model**

Note. A. based on ITT; B. based on PP; C. based on PP-IC; * means significant between-group mean difference.

1. **PA**

**Table S6. Longer-term effect intervention effect on PA based on raw GEE model.**

|  |  | **MD _ACG-MTPG_** | ***p* value** | **Group effect** | | **Time effect** | | **Group × Time effect** | |
| --- | --- | --- | --- | --- | --- | --- | --- | --- | --- |
|  |  |  |  | **Wald *χ*^2^** | ***p* value** | **Wald *χ*^2^** | ***p* value** | **Wald *χ*^2^** | ***p* value** |
| **ITT (n=160)** | T1 | -0.97 (-2.67, 0.73) | 0.264 | 3.105 | 0.078 | 7.244 | 0.065 | 8.435 | **0.038** |
|  | T3 | **-3.45 (-5.81, -1.08)** | **0.004** |  |  |  |  |  |  |
|  | T4 | -2.54 (-5.31, 0.24) | 0.074 |  |  |  |  |  |  |
|  | T5 | 1.33 (-1.51, 4.18) | 0.358 |  |  |  |  |  |  |
| **PP (n=160)** | T1 | -0.32 (-2.03, 1.40) | 0.718 | 1.628 | 0.202 | 6.146 | 0.105 | 8.194 | **0.042** |
|  | T3 | **-3.06 (-5.39, -0.74)** | **0.010** |  |  |  |  |  |  |
|  | T4 | -2.08 (-4.86, 0.69) | 0.141 |  |  |  |  |  |  |
|  | T5 | 1.41 (-1.35, 4.16) | 0.318 |  |  |  |  |  |  |
| **PP-IC (n=132)** | T1 | 0.48 (-1.58, 2.54) | 0.649 | 0.579 | 0.447 | 3.377 | 0.337 | 12.188 | **0.007** |
|  | T3 | **-3.39(-5.95, -0.83)** | **0.009** |  |  |  |  |  |  |
|  | T4 | -2.17 (-5.19, 0.84) | 0.157 |  |  |  |  |  |  |
|  | T5 | 2.17 (-0.97, 5.30) | 0.175 |  |  |  |  |  |  |

**Figure S3. Longer-term intervention effect on PA based on raw GEE model**


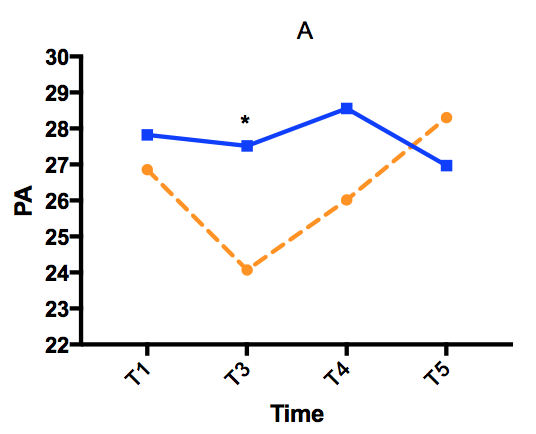

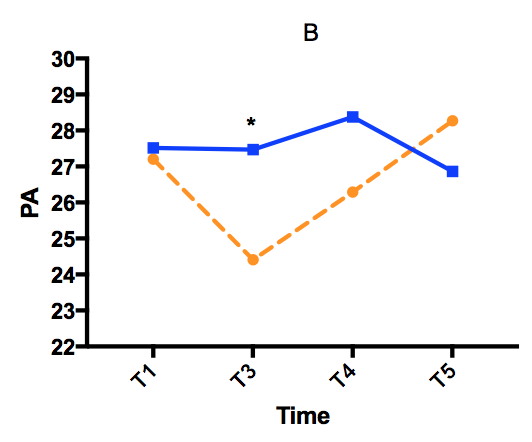

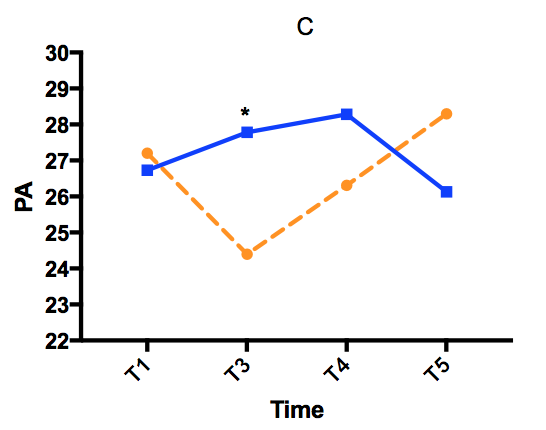

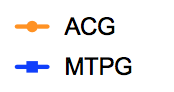


Note. A. based on ITT; B. based on PP; C. based on PP-IC; * means significant between-group mean difference.

1. **NA**

**Table S7. Longer-term effect intervention effect on log-transformed NA** based on raw GEE model.

|  |  | **MD _ACG-MTPG_** | ***p* value** | **Group effect** | | **Time effect** | | **Group × Time effect** | |
| --- | --- | --- | --- | --- | --- | --- | --- | --- | --- |
|  |  |  |  | **Wald *χ*^2^** | ***p* value** | **Wald *χ*^2^** | ***p* value** | **Wald *χ*^2^** | ***p* value** |
| **ITT (n=160)** | T1 | -0.03 (-0.07, 0.01) | 0.136 | 0.002 | 0.966 | 17.729 | **0.001** | 2.686 | 0.443 |
|  | T3 | 0.00 (-0.05, 0.05) | 0.945 |  |  |  |  |  |  |
|  | T4 | 0.02 (-0.04, 0.07) | 0.558 |  |  |  |  |  |  |
|  | T5 | 0.01 (-0.05, 0.06) | 0.823 |  |  |  |  |  |  |
| **PP (n=160)** | T1 | -0.03 (-0.07, 0.00) | 0.089 | 0.027 | 0.869 | 18.369 | **<0.001** | 3.964 | 0.265 |
|  | T3 | -0.01 (-0.06, 0.04) | 0.716 |  |  |  |  |  |  |
|  | T4 | 0.02 (-0.03, 0.08) | 0.407 |  |  |  |  |  |  |
|  | T5 | 0.01 (-0.05, 0.06) | 0.797 |  |  |  |  |  |  |
| **PP-IC (n=132)** | T1 | 0.01 (-0.04, 0.05) | 0.057 | 0.066 | 0.798 | 7.572 | 0.056 | 4.274 | 0.233 |
|  | T3 | 0.02(-0.03, 0.08) | 0.438 |  |  |  |  |  |  |
|  | T4 | -0.04 (-0.10, 0.02) | 0.220 |  |  |  |  |  |  |
|  | T5 | -0.01 (-0.08, 0.05) | 0.650 |  |  |  |  |  |  |


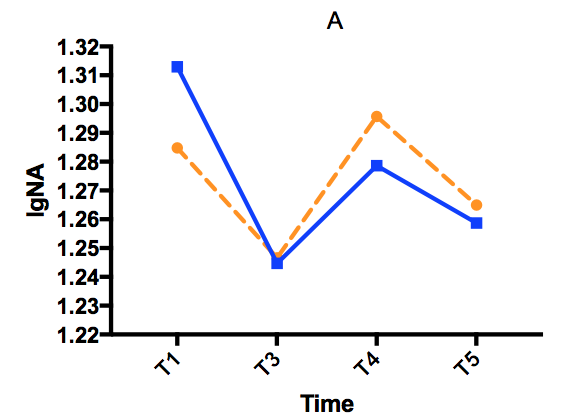

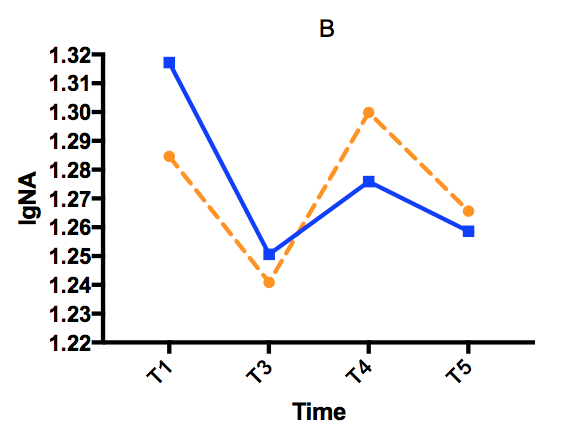

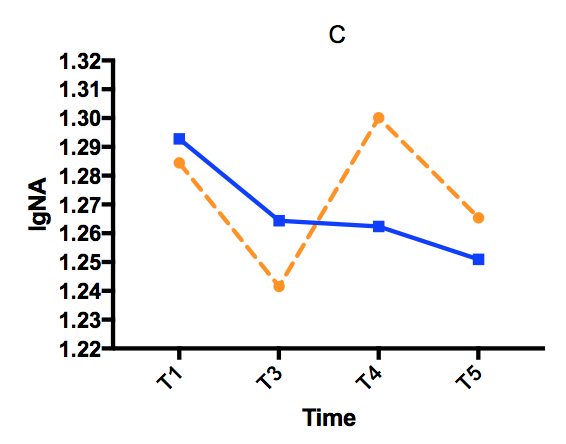

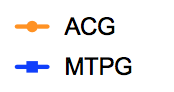


**Figure S4. Longer-term intervention effect on log-transformed NA based on raw GEE model**

Note. A. based on ITT; B. based on PP; C. based on PP-IC; * means significant between-group mean difference.

1. **PSQI**

**Table S8. Longer-term effect intervention effect on log-transformed PSQI** based on raw GEE model.

|  |  | **MD _ACG-MTPG_** | ***p* value** | **Group effect** | | **Time effect** | | **Group × Time effect** | |
| --- | --- | --- | --- | --- | --- | --- | --- | --- | --- |
|  |  |  |  | **Wald *χ*^2^** | ***p* value** | **Wald *χ*^2^** | ***p* value** | **Wald *χ*^2^** | ***p* value** |
| **ITT (n=160)** | T1 | -0.03 (-0.09, 0.03) | 0.360 | 0.031 | 0.861 | 18.098 | **<0.001** | 2.560 | 0.465 |
|  | T3 | 0.04 (-0.05, 0.12) | 0.410 |  |  |  |  |  |  |
|  | T4 | 0.01 (-0.08, 0.09) | 0.853 |  |  |  |  |  |  |
|  | T5 | -0.04 (-0.14, 0.06) | 0.463 |  |  |  |  |  |  |
| **PP (n=160)** | T1 | -0.04 (-0.10, 0.02) | 0.211 | 0.506 | 0.477 | 18.297 | **<0.001** | 2.810 | 0.422 |
|  | T3 | 0.02 (-0.07, 0.11) | 0.638 |  |  |  |  |  |  |
|  | T4 | -0.00 (-0.09, 0.08) | 0.949 |  |  |  |  |  |  |
|  | T5 | -0.06 (-0.16, 0.03) | 0.199 |  |  |  |  |  |  |
| **PP-IC (n=131)** | T1 | 0.01 (-0.05, 0.08) | 0.720 | 0.053 | 0.819 | 17.269 | **0.001** | 3.013 | 0.390 |
|  | T3 | -0.05(-0.14, 0.04) | 0.300 |  |  |  |  |  |  |
|  | T4 | -0.04 (-0.14, 0.06) | 0.449 |  |  |  |  |  |  |
|  | T5 | 0.04 (-0.08, 0.16) | 0.484 |  |  |  |  |  |  |


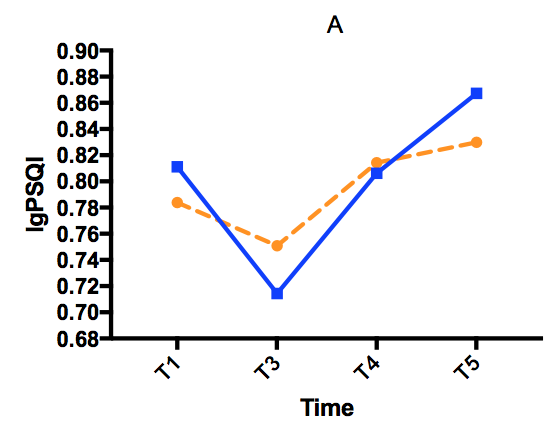

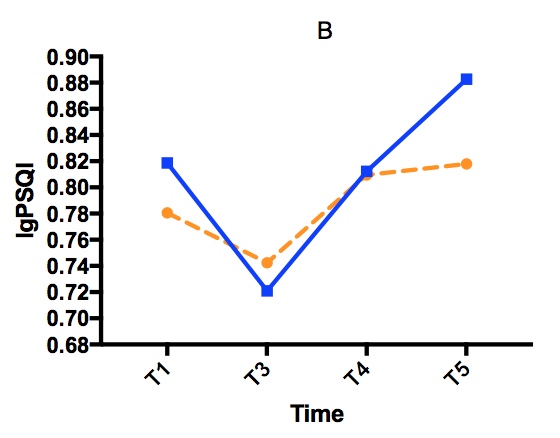

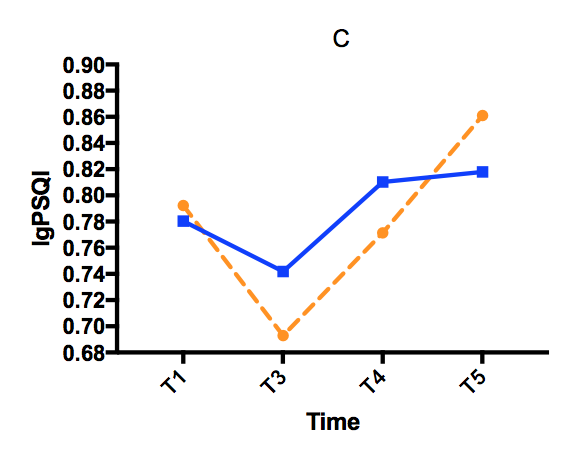

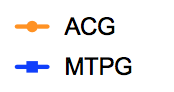


**Figure S5. Longer-term intervention effect on log-transformed PSQI based on raw GEE model**

Note. A. based on ITT; B. based on PP; C. based on PP-IC; * means significant between-group mean difference.

1. **FSS**

**Table S9. Longer-term effect intervention effect on FSS** based on raw GEE model.

|  |  | **MD _ACG-MTPG_** | ***p* value** | **Group effect** | | **Time effect** | | **Group × Time effect** | |
| --- | --- | --- | --- | --- | --- | --- | --- | --- | --- |
|  |  |  |  | **Wald *χ*^2^** | ***p* value** | **Wald *χ*^2^** | ***p* value** | **Wald *χ*^2^** | ***p* value** |
| **ITT (n=161)** | T1 | 1.44 (-1.50, 4.39) | 0.337 | 1.298 | 0.255 | 13.363 | **0.004** | 1.394 | 0.707 |
|  | T3 | 0.93 (-3.00, 4.87) | 0.644 |  |  |  |  |  |  |
|  | T4 | 3.09 (-0.72, 6.91) | 0.112 |  |  |  |  |  |  |
|  | T5 | 0.42 (-4.00, 4.84) | 0.852 |  |  |  |  |  |  |
| **PP (n=161)** | T1 | 2.10 (-0.88, 5.08) | 0.167 | 0.601 | 0.438 | 12.716 | **0.005** | 2.799 | 0.424 |
|  | T3 | 0.30 (-3.54, 4.15) | 0.877 |  |  |  |  |  |  |
|  | T4 | 2.72 (-1.09, 6.54) | 0.162 |  |  |  |  |  |  |
|  | T5 | -1.15 (-5.59, 3.29) | 0.612 |  |  |  |  |  |  |
| **PP-IC (n=133)** | T1 | 2.98 (-0.41, 6.37) | 0.085 | 0.983 | 0.322 | 6.941 | 0.074 | 2.945 | 0.400 |
|  | T3 | 0.80 (-3.46, 5.05) | 0.714 |  |  |  |  |  |  |
|  | T4 | 3.42 (-0.88, 7.73) | 0.119 |  |  |  |  |  |  |
|  | T5 | -1.00 (-6.44, 4.44) | 0.720 |  |  |  |  |  |  |


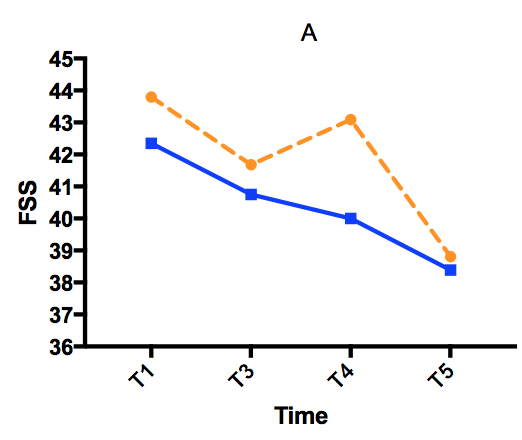

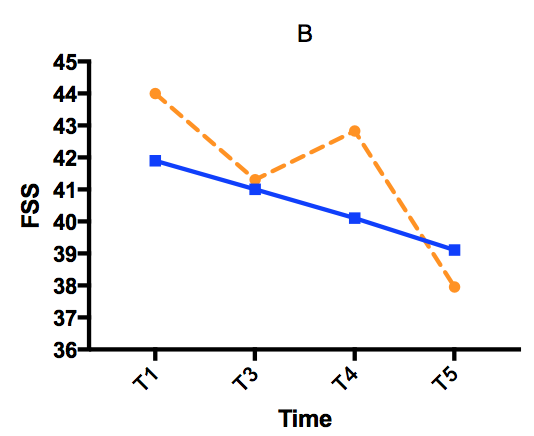

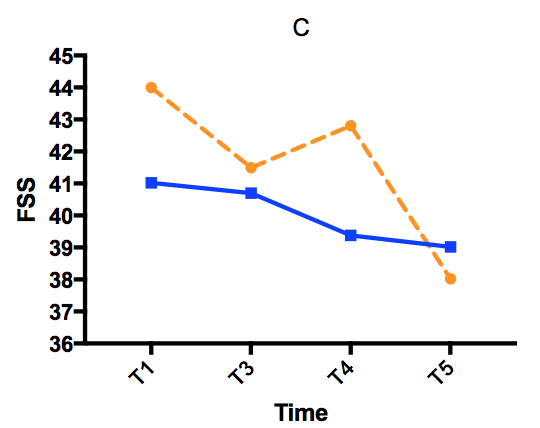

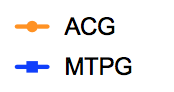


**Figure S6. Longer-term intervention effect on FSS based on raw GEE model**

Note. A. based on ITT; B. based on PP; C. based on PP-IC; * means significant between-group mean difference

1. **PM**

**Table S10. Longer-term effect intervention effect on log-transformed PM** based on raw GEE model.

|  |  | **MD _ACG-MTPG_** | ***p* value** | **Group effect** | | **Time effect** | | **Group × Time effect** | |
| --- | --- | --- | --- | --- | --- | --- | --- | --- | --- |
|  |  |  |  | **Wald *χ*^2^** | ***p* value** | **Wald *χ*^2^** | ***p* value** | **Wald *χ*^2^** | ***p* value** |
| **ITT (n=160)** | T1 | 0.01 (-0.03, 0.05) | 0.761 | 0.746 | 0.388 | 1.927 | 0.382 | 3.713 | 0.156 |
|  | T3 | -0.04 (-0.09, 0.02) | 0.180 |  |  |  |  |  |  |
|  | T5 | -0.03 (-0.10, 0.05) | 0.453 |  |  |  |  |  |  |
| **PP (n=160)** | T1 | 0.00 (-0.04, 0.04) | 0.885 | 1.187 | 0.276 | 2.418 | 0.299 | 3.114 | 0.211 |
|  | T3 | -0.03 (-0.08, 0.02) | 0.271 |  |  |  |  |  |  |
|  | T5 | -0.05 (-0.12, 0.03) | 0.210 |  |  |  |  |  |  |
| **PP-IC (n=131)** | T1 | 0.01 (-0.04, 0.06) | 0.709 | 0.891 | 0.345 | 3.634 | 0.162 | 4.183 | 0.123 |
|  | T3 | -0.04 (-0.10, 0.03) | 0.253 |  |  |  |  |  |  |
|  | T5 | -0.05 (-0.13, 0.03) | 0.244 |  |  |  |  |  |  |


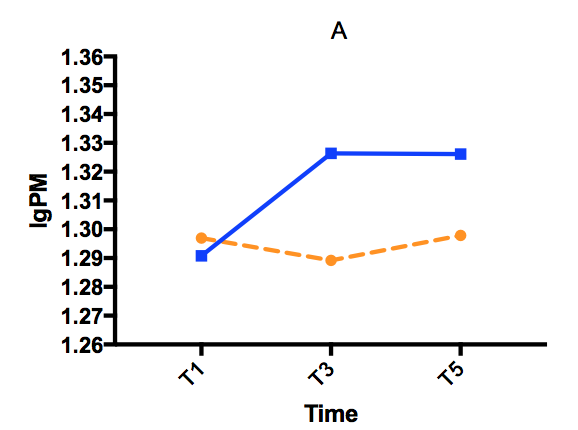

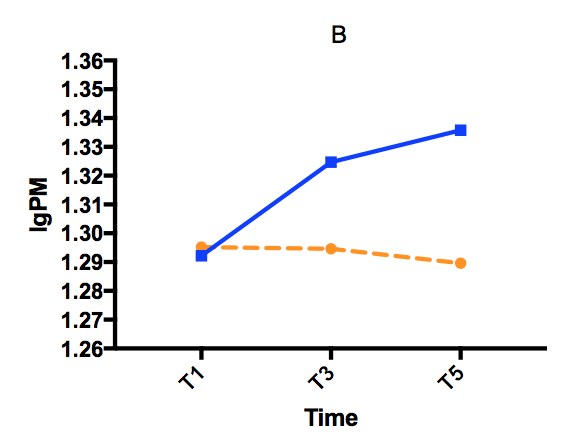

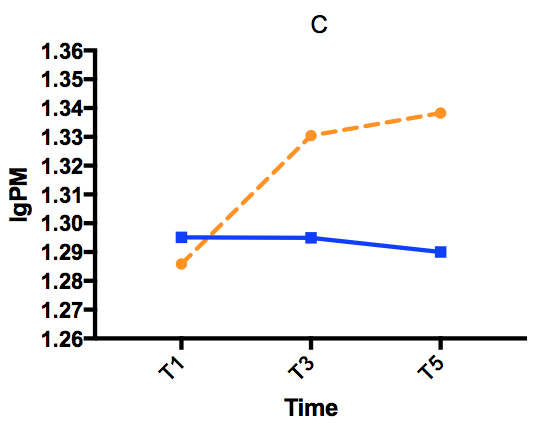

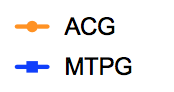


**Figure S7. Longer-term intervention effect on log-transformed PM based on raw GEE model**

Note. A. based on ITT; B. based on PP; C. based on PP-IC; * means significant between-group mean difference

1. **RM**

**Table S11. Longer-term effect intervention effect on RM** based on raw GEE model.

|  |  | **MD _ACG-MTPG_** | ***p* value** | **Group effect** | | **Time effect** | | **Group × Time effect** | |
| --- | --- | --- | --- | --- | --- | --- | --- | --- | --- |
|  |  |  |  | **Wald *χ*^2^** | ***p* value** | **Wald *χ*^2^** | ***p* value** | **Wald *χ*^2^** | ***p* value** |
| **ITT (n=160)** | T1 | 0.58 (-1.35, 2.51) | 0.554 | 0.134 | 0.714 | 12.515 | **0.002** | 2.589 | 0.274 |
|  | T3 | -0.95 (-3.49, 1.58) | 0.462 |  |  |  |  |  |  |
|  | T5 | -0.78 (-3.97, 2.42) | 0.633 |  |  |  |  |  |  |
| **PP (n=160)** | T1 | 0.72 (-1.23, 2.66) | 0.470 | 0.147 | 0.701 | 13.234 | **0.001** | 3.011 | 0.222 |
|  | T3 | -0.28 (-2.82, 2.27) | 0.832 |  |  |  |  |  |  |
|  | T5 | -1.63 (-4.79, 1.53) | 0.312 |  |  |  |  |  |  |
| **PP-IC (n=131)** | T1 | -1.03 (-3.33, 1.27) | 0.379 | 0.156 | 0.693 | 15.718 | **<0.001** | 4.684 | 0.096 |
|  | T3 | 0.72 (-2.14, 3.58) | 0.622 |  |  |  |  |  |  |
|  | T5 | 1.73 (-1.83, 5.29) | 0.340 |  |  |  |  |  |  |


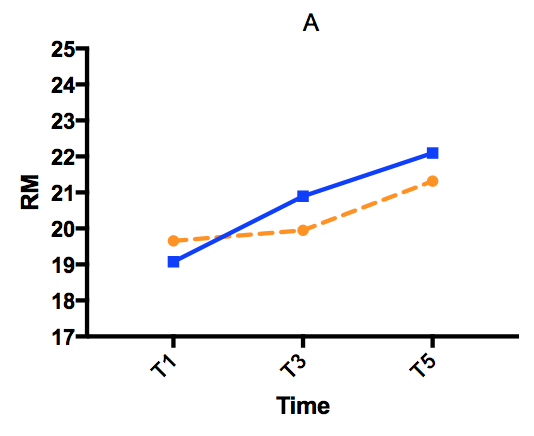

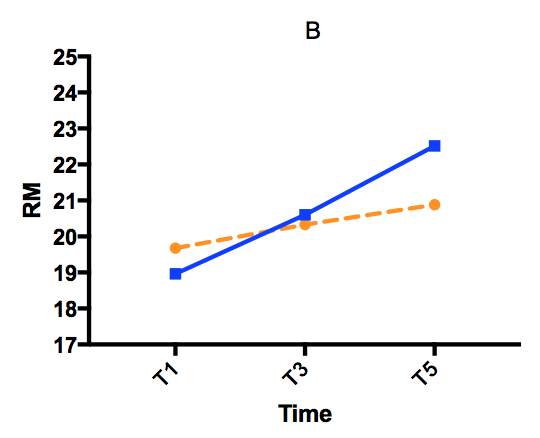

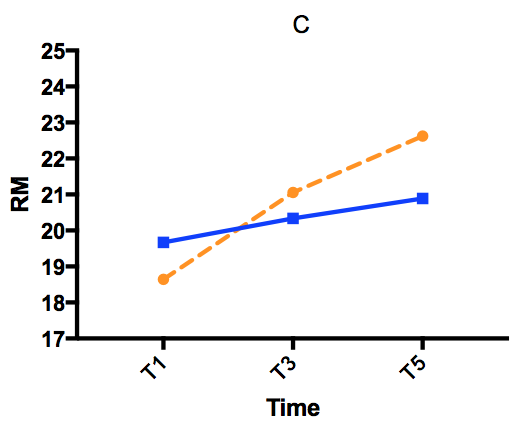

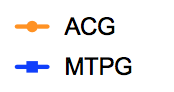


**Figure S8. Longer-term intervention effect on RM based on raw GEE model**

Note. A. based on ITT; B. based on PP; C. based on PP-IC; * means significant between-group mean difference

1. **WDEQ**

**Table S12. Longer-term effect intervention effect on WDEQ** based on raw GEE model.

|  |  | **MD _ACG-MTPG_** | ***p* value** | **Group effect** | | **Time effect** | | **Group × Time effect** | |
| --- | --- | --- | --- | --- | --- | --- | --- | --- | --- |
|  |  |  |  | **Wald *χ*^2^** | ***p* value** | **Wald *χ*^2^** | ***p* value** | **Wald *χ*^2^** | ***p* value** |
| **ITT (n=161)** | T1 | 1.59 (-3.81, 6.99) | 0.564 | 2.447 | 0.118 | 9.515 | **0.023** | 4.789 | 0.188 |
|  | T2 | 7.48 (0.57, 14.39) | **0.034** |  |  |  |  |  |  |
|  | T3 | 5.88 (-0.72, 12.48) | 0.081 |  |  |  |  |  |  |
|  | T4 | 1.65 (-5.71, 9.01) | 0.661 |  |  |  |  |  |  |
| **PP (n=161)** | T1 | 0.51 (-4.89, 5.92) | 0.852 | 0.679 | 0.410 | 9.955 | **0.019** | 3.149 | 0.369 |
|  | T2 | 4.58 (-2.45, 11.62) | 0.202 |  |  |  |  |  |  |
|  | T3 | 4.10 (-2.54, 10.73) | 0.226 |  |  |  |  |  |  |
|  | T4 | -0.27 (-7.70, 7.16) | 0.943 |  |  |  |  |  |  |
| **PP-IC (n=133)** | T1 | -1.08 (-7.38, 5.23) | 0.738 | 0.275 | 0.600 | 7.152 | 0.067 | 0.946 | 0.814 |
|  | T2 | -2.86 (-10.95, 5.22) | 0.488 |  |  |  |  |  |  |
|  | T3 | -3.08 (-10.84, 4.68) | 0.437 |  |  |  |  |  |  |
|  | T4 | 0.21 (-8.41, 8.83) | 0.962 |  |  |  |  |  |  |


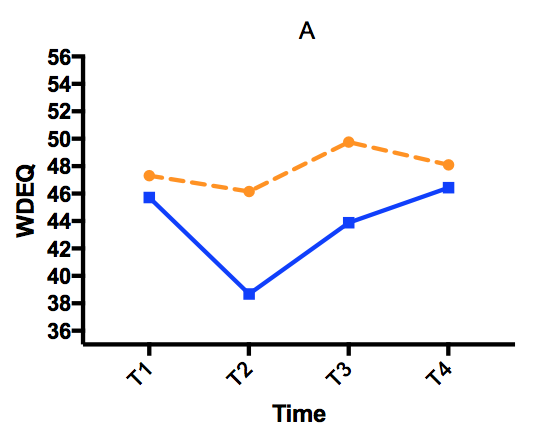

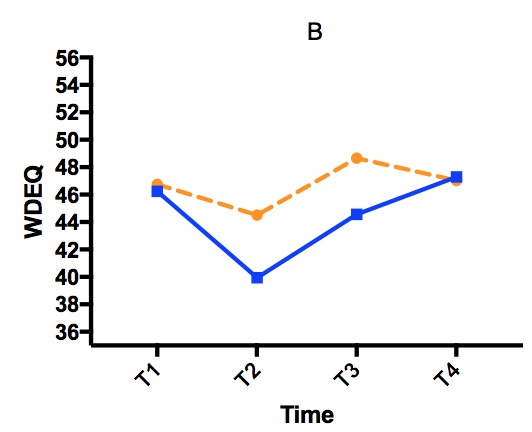

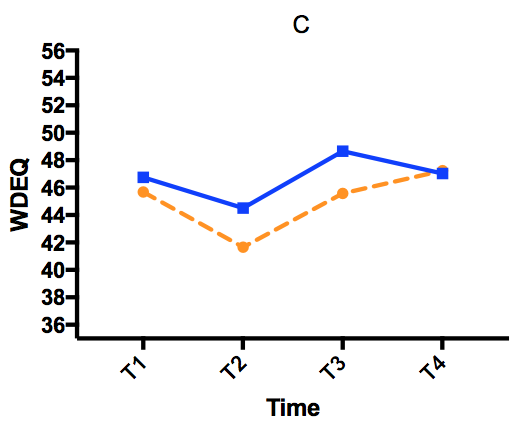

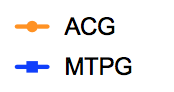


**Figure S9. Longer-term intervention effect on WDEQ based on raw GEE model**

Note. A. based on ITT; B. based on PP; C. based on PP-IC; * means significant between-group mean difference
