# Supplementary material for: Effectiveness of Smartphone-Based Mindfulness Training on Maternal Perinatal Depression: Randomized Controlled Trial
Source: J Med Internet Res. 2021 Jan 27;23(1):e23410. doi: 10.2196/23410 (PMC7875700; doi:10.2196/23410)
Supplement: Multimedia Appendix 6 [file jmir_v23i1e23410_app6.doc]

# **GEE model of subgroup analysis**

**Table S13. GEE model effects on group, time and parity.**

|  |  | **Group** | **Time** | **Parity** | **Group × Time** | **Group × Parity** | **Time ×Parity** | **Group × Time × Parity** |
| --- | --- | --- | --- | --- | --- | --- | --- | --- |
| **EPDS (n=168)** | **Wald *χ*^2^** | 0.975 | 15.305 | 0.094 | 11.304 | 0.097 | 1.511 | 5.555 |
|  | ***p* value** | 0.323 | **0.004** | 0.759 | **0.023** | 0.755 | 0.825 | 0.235 |
| **GAD-7 (n=166)** | **Wald *χ*^2^** | 0.614 | 14.102 | 0.359 | 10.264 | 0.878 | 1.445 | 7.766 |
|  | ***p* value** | 0.433 | **0.007** | 0.549 | **0.036** | 0.349 | 0.836 | 0.101 |
| **PSS (n=166)** | **Wald *χ*^2^** | 1.577 | 6.506 | 0.001 | 4.222 | 0.026 | 3.575 | 2.577 |
|  | ***p* value** | 0.209 | 0.164 | 0.972 | 0.377 | 0.873 | 0.467 | 0.631 |
| **PA (n=160)** | **Wald *χ*^2^** | 3.621 | 9.779 | 4.818 | 5.846 | 0.421 | 2.751 | 2.330 |
|  | ***p* value** | 0.057 | **0.021** | **0.028** | 0.119 | 0.517 | 0.432 | 0.507 |
| **lgNA (n=160)** | **Wald *χ*^2^** | 0.065 | 19.056 | 0.039 | 4.700 | 0.907 | 0.712 | 4.071 |
|  | ***p* value** | 0.798 | **<0.001** | 0.844 | 0.195 | 0.341 | 0.870 | 0.254 |
| **lgPSQI (n=160)** | **Wald *χ*^2^** | 0.001 | 17.065 | 0.026 | 2.714 | 0.173 | 4.101 | 0.387 |
|  | ***p* value** | 0.980 | **0.001** | 0.871 | 0.438 | 0.677 | 0.251 | 0.943 |
| **FSS (n=161)** | **Wald *χ*^2^** | 2.014 | 13.764 | 0.081 | 0.944 | 0.990 | 3.724 | 3.447 |
|  | ***p* value** | 0.156 | **0.003** | 0.776 | 0.815 | 0.320 | 0.293 | 0.328 |
| **lgPM (n=160)** | **Wald *χ*^2^** | 0.354 | 0.868 | 0.095 | 2.266 | 0.253 | 1.848 | 3.604 |
|  | ***p* value** | 0.552 | 0.648 | 0.758 | 0.322 | 0.615 | 0.397 | 0.165 |
| **RM (n=160)** | **Wald *χ*^2^** | 0.059 | 7.896 | 0.074 | 0.880 | 0.029 | 2.339 | 3.904 |
|  | ***p* value** | 0.809 | **0.019** | 0.786 | 0.644 | 0.866 | 0.311 | 0.142 |
| **WDEQ**  **(n=161)** | **Wald *χ*^2^** | 3.687 | 12.575 | 0.709 | 4.005 | 1.413 | 2.434 | 4.884 |
|  | ***p* value** | 0.055 | **0.006** | 0.400 | 0.261 | 0.235 | 0.487 | 0.181 |
