# Supplementary material for: Effectiveness of Smartphone-Based Mindfulness Training on Maternal Perinatal Depression: Randomized Controlled Trial
Source: J Med Internet Res. 2021 Jan 27;23(1):e23410. doi: 10.2196/23410 (PMC7875700; doi:10.2196/23410)
Supplement: Multimedia Appendix 7 [file jmir_v23i1e23410_app7.doc]

# **Subgroup analysis on secondary outcomes**

1. **GAD-7**

**Table S14. Longer-term effect intervention effect on GAD-7 by parity.**

|  |  | **MD _ACG-MTPG_** | ***p* value** | **Group effect** | | **Time effect** | | **Group × Time effect** | |
| --- | --- | --- | --- | --- | --- | --- | --- | --- | --- |
|  |  |  |  | **Wald *χ*^2^** | ***p* value** | **Wald *χ*^2^** | ***p* value** | **Wald *χ*^2^** | ***p* value** |
| **Primipara (n=109)** | T1 | -1.00 (-2.19, 0.18) | 0.098 | 0.027 | 0.870 | 19.691 | **0.001** | 18.565 | **0.001** |
|  | T2 | -0.87 (-2.18, 0.44) | 0.192 |  |  |  |  |  |  |
|  | T3 | 1.64 (-0.28, 3.55) | 0.093 |  |  |  |  |  |  |
|  | T4 | 1.13 (-0.54, 2.79) | 0.185 |  |  |  |  |  |  |
|  | T5 | -1.33 (-3.06, 0.40) | 0.132 |  |  |  |  |  |  |
| **Multipara (n=57)** | T1 | -0.73 (-2.64, 1.18) | 0.453 | 1.251 | 0.263 | 3.342 | 0.502 | 6.314 | 0.177 |
|  | T2 | 0.77 (-1.50, 3.04) | 0.506 |  |  |  |  |  |  |
|  | T3 | 0.39 (-1.68, 2.47) | 0.710 |  |  |  |  |  |  |
|  | T4 | 1.24 (-0.95, 3.44) | 0.267 |  |  |  |  |  |  |
|  | T5 | 1.98 (-0.15, 4.11) | 0.069 |  |  |  |  |  |  |


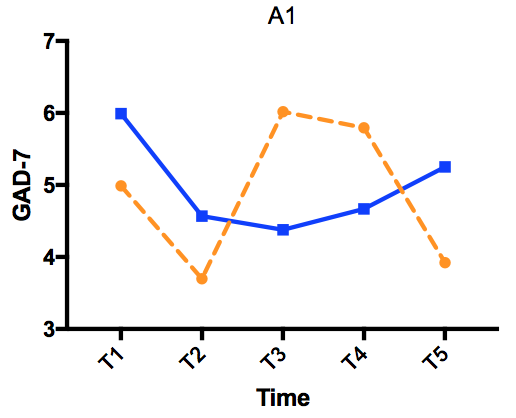

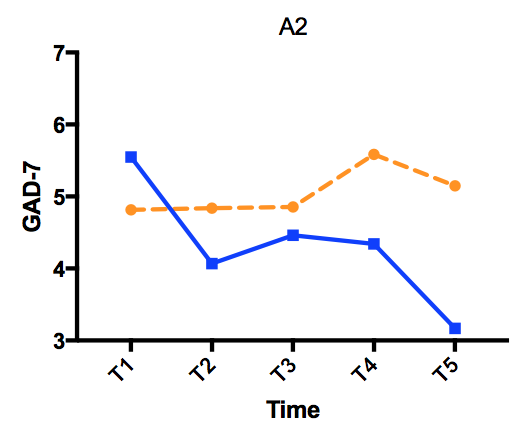

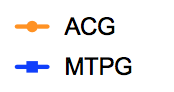


**Figure S10. Longer-term intervention effect on GAD-7 by parity**

Note. A1. Model in primipara; A2. Model in multipara; * means significant between-group mean difference.

1. **PSS**

**Table S15. Longer-term effect intervention effect on PSS by parity.**

|  |  | **MD _ACG-MTPG_** | ***p* value** | **Group effect** | | **Time effect** | | **Group × Time effect** | |
| --- | --- | --- | --- | --- | --- | --- | --- | --- | --- |
|  |  |  |  | **Wald *χ*^2^** | ***p* value** | **Wald *χ*^2^** | ***p* value** | **Wald *χ*^2^** | ***p* value** |
| **Primipara (n=109)** | T1 | 0.17 (-0.67, 1.00) | 0.697 | 1.001 | 0.317 | 20.891 | **<0.001** | 3.834 | 0.429 |
|  | T2 | 0.78 (-0.32, 1.88) | 0.164 |  |  |  |  |  |  |
|  | T3 | 0.88 (-0.54, 2.30) | 0.224 |  |  |  |  |  |  |
|  | T4 | 0.38 (-1.18, 1.93) | 0.633 |  |  |  |  |  |  |
|  | T5 | -0.26 (-1.20, 0.68) | 0.586 |  |  |  |  |  |  |
| **Multipara (n=57)** | T1 | -0.10 (-1.52, 1.33) | 0.893 | 1.147 | 0.887 | 0.725 | 0.394 | 2.804 | 0.591 |
|  | T2 | -0.28 (-1.95, 1.39) | 0.739 |  |  |  |  |  |  |
|  | T3 | 1.17 (-0.71, 3.06) | 0.222 |  |  |  |  |  |  |
|  | T4 | 1.32 (-0.48, 3.12) | 0.150 |  |  |  |  |  |  |
|  | T5 | 0.35 (-1.37, 2.07) | 0.692 |  |  |  |  |  |  |


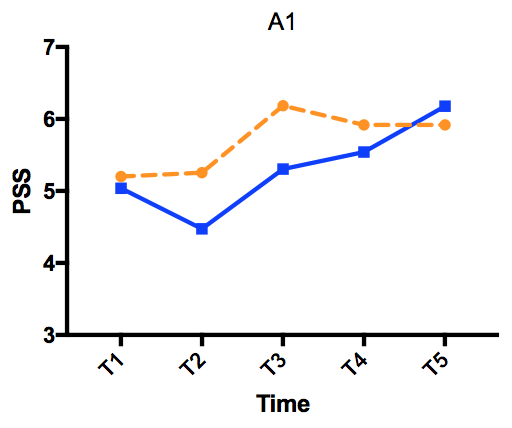

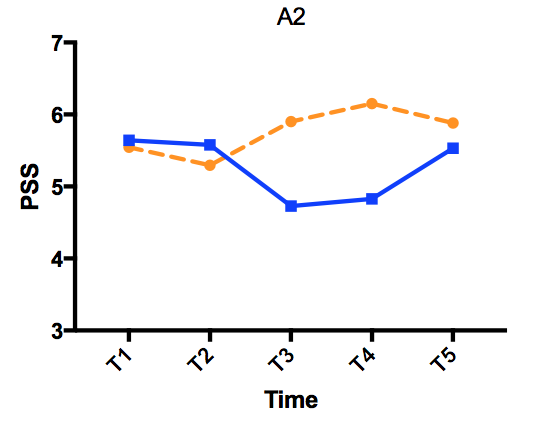

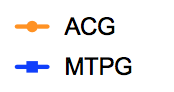


**Figure S11. Longer-term intervention effect on PSS by parity**

Note. A1. Model in primipara; A2. Model in multipara; * means significant between-group mean difference.

1. **PA**

**Table S16. Longer-term effect intervention effect on PA by parity.**

|  |  | **MD _ACG-MTPG_** | ***p* value** | **Group effect** | | **Time effect** | | **Group × Time effect** | |
| --- | --- | --- | --- | --- | --- | --- | --- | --- | --- |
|  |  |  |  | **Wald *χ*^2^** | ***p* value** | **Wald *χ*^2^** | ***p* value** | **Wald *χ*^2^** | ***p* value** |
| **Primipara (n=105)** | T1 | -0.07 (-2.08, 1.94) | 0.944 | 1.061 | 0.303 | 3.397 | 0.334 | 10.399 | **0.015** |
|  | T3 | **-3.63 (-6.55, -0.71)** | **0.015** |  |  |  |  |  |  |
|  | T4 | -2.94 (-6.54, 0.65) | 0.109 |  |  |  |  |  |  |
|  | T5 | 2.55 (-0.81, 5.90) | 0.136 |  |  |  |  |  |  |
| **Multipara (n=55)** | T1 | -2.71 (-5.66, 0.23) | 0.071 | 2.611 | 0.106 | 8.608 | **0.035** | 0.858 | 0.836 |
|  | T3 | -3.00 (-6.82, 0.81) | 0.122 |  |  |  |  |  |  |
|  | T4 | -1.66 (-5.88, 2.62) | 0.454 |  |  |  |  |  |  |
|  | T5 | -0.90 (-6.00, 4.20) | 0.729 |  |  |  |  |  |  |


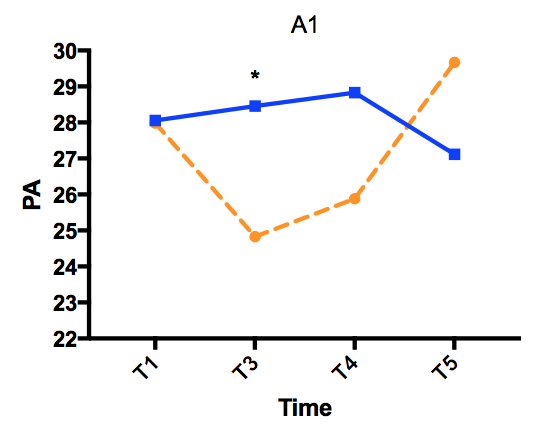

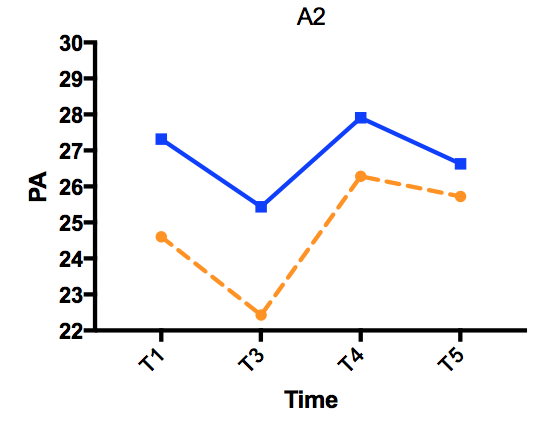

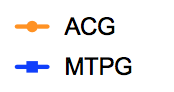


**Figure S12. Longer-term intervention effect on PA by parity**

Note. A1. Model in primipara; A2. Model in multipara; * means significant between-group mean difference.

1. **NA**

**Table S17. Longer-term effect intervention effect on log-transformed NA by parity.**

|  |  | **MD _ACG-MTPG_** | ***p* value** | **Group effect** | | **Time effect** | | **Group × Time effect** | |
| --- | --- | --- | --- | --- | --- | --- | --- | --- | --- |
|  |  |  |  | **Wald *χ*^2^** | ***p* value** | **Wald *χ*^2^** | ***p* value** | **Wald *χ*^2^** | ***p* value** |
| **Primipara (n=105)** | T1 | -0.02 (-0.06, 0.02) | 0.397 | 0.344 | 0.557 | 9.998 | **0.019** | 0.313 | 0.957 |
|  | T3 | -0.00 (-0.07, 0.07) | 0.946 |  |  |  |  |  |  |
|  | T4 | -0.01 (-0.08, 0.06) | 0.727 |  |  |  |  |  |  |
|  | T5 | -0.02 (-0.08, 0.04) | 0.547 |  |  |  |  |  |  |
| **Multipara (n=55)** | T1 | -0.05 (-0.12, 0.02) | 0.136 | 0.664 | 0.415 | 9.971 | **0.019** | 5.732 | 0.125 |
|  | T3 | 0.01 (-0.07, 0.09) | 0.811 |  |  |  |  |  |  |
|  | T4 | 0.07 (-0.03, 0.16) | 0.179 |  |  |  |  |  |  |
|  | T5 | 0.06 (-0.04, 0.15) | 0.226 |  |  |  |  |  |  |


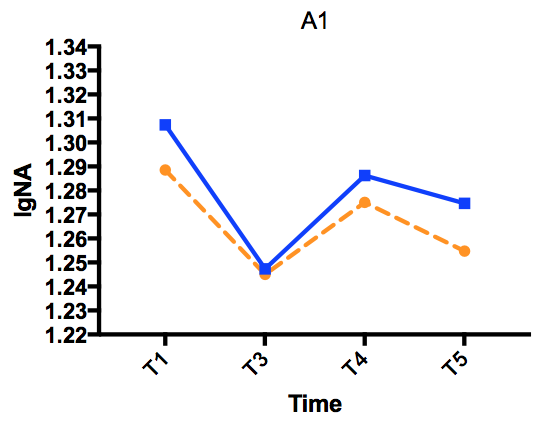

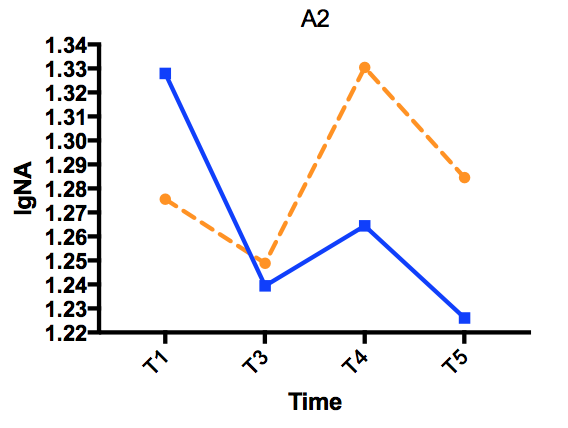

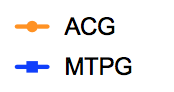


**Figure S13. Longer-term intervention effect on log-transformed NA by parity**

Note. A1. Model in primipara; A2. Model in multipara; * means significant between-group mean difference.

1. **PSQI**

**Table S18. Longer-term effect intervention effect on log-transformed PSQI by parity.**

|  |  | **MD _ACG-MTPG_** | ***p* value** | **Group effect** | | **Time effect** | | **Group × Time effect** | |
| --- | --- | --- | --- | --- | --- | --- | --- | --- | --- |
|  |  |  |  | **Wald *χ*^2^** | ***p* value** | **Wald *χ*^2^** | ***p* value** | **Wald *χ*^2^** | ***p* value** |
| **Primipara (n=105)** | T1 | -0.05 (-0.12, 0.02) | 0.132 | 0.219 | 0.640 | 17.410 | **0.001** | 1.728 | 0.631 |
|  | T3 | 0.02 (-0.08, 0.13) | 0.656 |  |  |  |  |  |  |
|  | T4 | 0.00 (-0.12, 0.12) | 0.967 |  |  |  |  |  |  |
|  | T5 | -0.04 (-0.14, 0.07) | 0.502 |  |  |  |  |  |  |
| **Multipara (n=55)** | T1 | 0.00 (-0.11, 0.11) | 0.965 | 0.015 | 0.902 | 7.871 | **0.049** | 1.540 | 0.673 |
|  | T3 | 0.05 (-0.09, 0.19) | 0.507 |  |  |  |  |  |  |
|  | T4 | 0.02 (-0.10, 0.14) | 0.735 |  |  |  |  |  |  |
|  | T5 | -0.04 (-0.25, 0.16) | 0.695 |  |  |  |  |  |  |


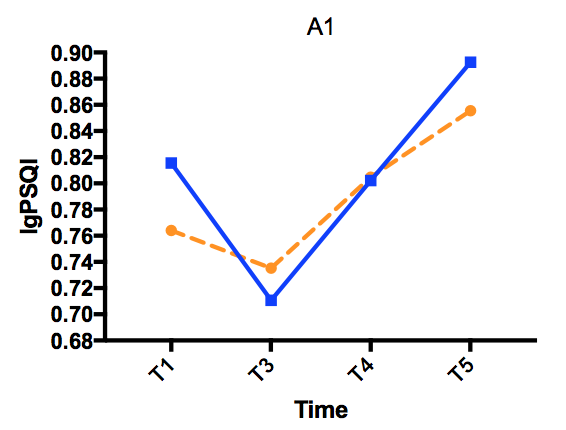

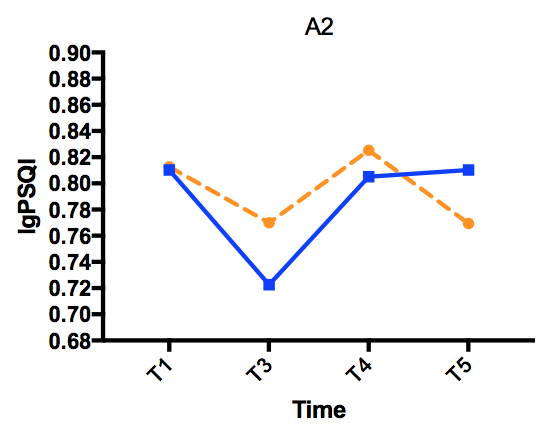

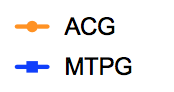


**Figure S14. Longer-term intervention effect on log-transformed PSQI by parity**

Note. A1. Model in primipara; A2. Model in multipara; * means significant between-group mean difference.

1. **FSS**

**Table S19. Longer-term effect intervention effect on FSS by parity.**

|  |  | **MD _ACG-MTPG_** | ***p* value** | **Group effect** | | **Time effect** | | **Group × Time effect** | |
| --- | --- | --- | --- | --- | --- | --- | --- | --- | --- |
|  |  |  |  | **Wald *χ*^2^** | ***p* value** | **Wald *χ*^2^** | ***p* value** | **Wald *χ*^2^** | ***p* value** |
| **Primipara (n=106)** | T1 | 0.53 (-2.86, 3.91) | 0.761 | 0.127 | 0.721 | 9.215 | **0.027** | 2.478 | 0.479 |
|  | T3 | 1.51 (-3.39, 6.40) | 0.547 |  |  |  |  |  |  |
|  | T4 | 2.36 (-2.45, 7.16) | 0.336 |  |  |  |  |  |  |
|  | T5 | -2.04 (-7.50, 3.41) | 0.462 |  |  |  |  |  |  |
| **Multipara (n=55)** | T1 | 3.22 (-2.54, 8.98) | 0.273 | 2.212 | 0.137 | 10.711 | **0.013** | 1.830 | 0.608 |
|  | T3 | -0.20 (-6.71, 6.30) | 0.951 |  |  |  |  |  |  |
|  | T4 | 4.08 (-2.19, 10.35) | 0.202 |  |  |  |  |  |  |
|  | T5 | 5.26 (-2.20, 12.73) | 0.167 |  |  |  |  |  |  |


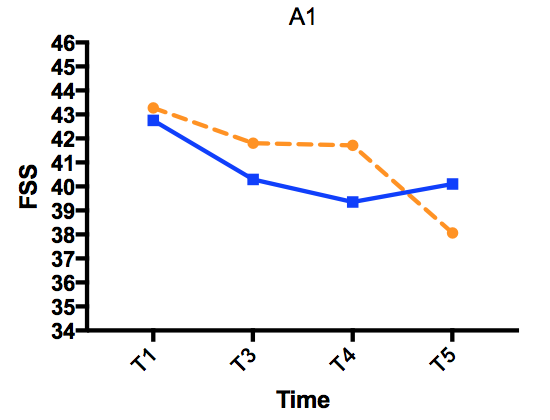

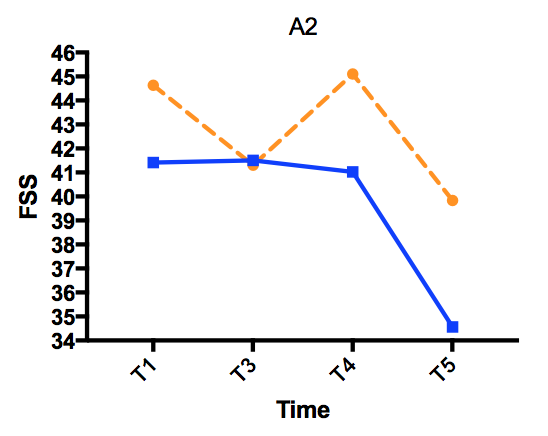

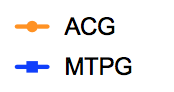


**Figure S15. Longer-term intervention effect on FSS by parity**

Note. A1. Model in primipara; A2. Model in multipara; * means significant between-group mean difference.

1. **PM**

**Table S20. Longer-term effect intervention effect on log-transformed PM by parity.**

|  |  | **MD _ACG-MTPG_** | ***p* value** | **Group effect** | | **Time effect** | | **Group × Time effect** | |
| --- | --- | --- | --- | --- | --- | --- | --- | --- | --- |
|  |  |  |  | **Wald *χ*^2^** | ***p* value** | **Wald *χ*^2^** | ***p* value** | **Wald *χ*^2^** | ***p* value** |
| **Primipara (n=106)** | T1 | 0.02 (-0.03, 0.07) | 0.426 | 0.915 | 0.339 | 4.427 | 0.109 | 6.798 | 0.033 |
|  | T3 | -0.04 (-0.10, 0.03) | 0.299 |  |  |  |  |  |  |
|  | T5 | -0.07 (-0.15, 0.02) | 0.152 |  |  |  |  |  |  |
| **Multipara (n=54)** | T1 | -0.02 (-0.09, 0.06) | 0.637 | 0.009 | 0.925 | 0.415 | 0.813 | 1.683 | 0.431 |
|  | T3 | -0.04 (-0.14, 0.06) | 0.422 |  |  |  |  |  |  |
|  | T5 | 0.05 (-0.09, 0.18) | 0.499 |  |  |  |  |  |  |


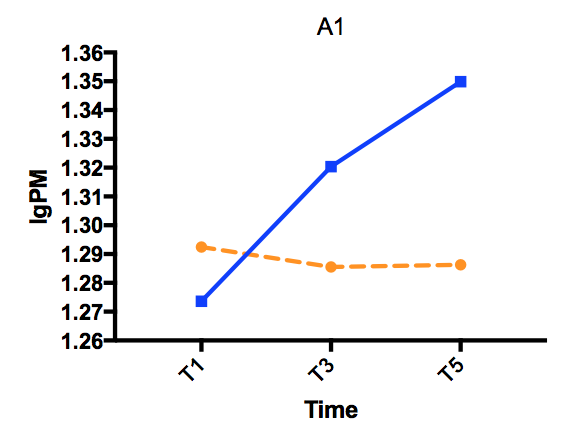

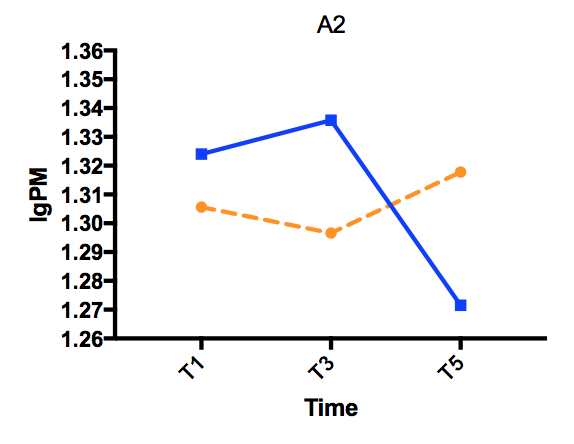

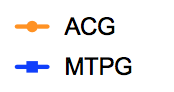


**Figure S16. Longer-term intervention effect on log-transformed PM by parity**

Note. A1. Model in primipara; A2. Model in multipara; * means significant between-group mean difference.

1. **RM**

**Table S21. Longer-term effect intervention effect on RM by parity.**

|  |  | **MD _ACG-MTPG_** | ***p* value** | **Group effect** | | **Time effect** | | **Group × Time effect** | |
| --- | --- | --- | --- | --- | --- | --- | --- | --- | --- |
|  |  |  |  | **Wald *χ*^2^** | ***p* value** | **Wald *χ*^2^** | ***p* value** | **Wald *χ*^2^** | ***p* value** |
| **Primipara (n=106)** | T1 | 1.34 (-0.74, 3.42) | 0.207 | 0.148 | 0.701 | 16.313 | **<0.001** | 6.117 | 0.047 |
|  | T3 | -0.99 (-4.07, 2.08) | 0.527 |  |  |  |  |  |  |
|  | T5 | -1.77 (-5.50, 1.96) | 0.353 |  |  |  |  |  |  |
| **Multipara (n=54)** | T1 | -0.97 (-4.93, 2.99) | 0.632 | 0.001 | 0.973 | 0.603 | 0.744 | 0.806 | 0.668 |
|  | T3 | -0.69 (-5.12, 3.74) | 0.759 |  |  |  |  |  |  |
|  | T5 | 1.46 (-4.38, 7.31) | 0.624 |  |  |  |  |  |  |


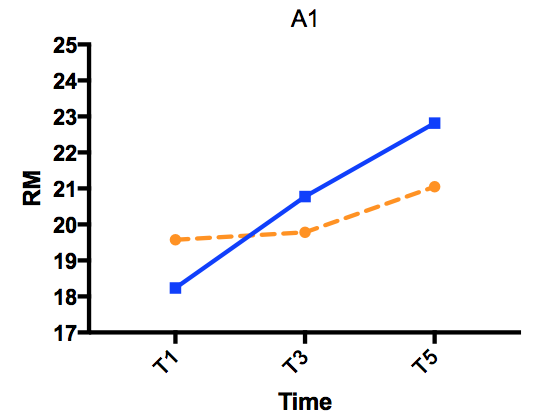

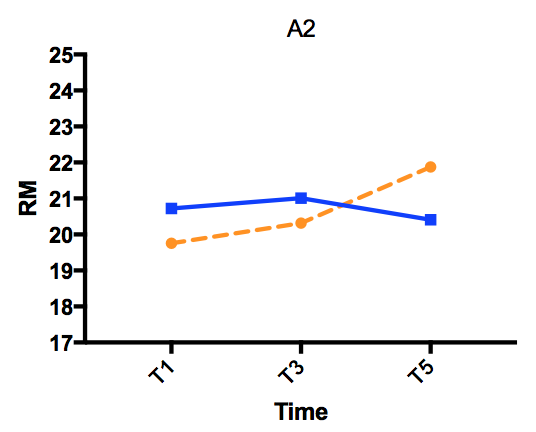

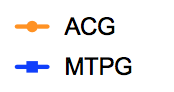


**Figure S17. Longer-term intervention effect on RM by parity**

Note. A1. Model in primipara; A2. Model in multipara; * means significant between-group mean difference.

1. **WDEQ**

**Table S22. Longer-term effect intervention effect on WDEQ by parity.**

|  |  | **MD _ACG-MTPG_** | ***p* value** | **Group effect** | | **Time effect** | | **Group × Time effect** | |
| --- | --- | --- | --- | --- | --- | --- | --- | --- | --- |
|  |  |  |  | **Wald *χ*^2^** | ***p* value** | **Wald *χ*^2^** | ***p* value** | **Wald *χ*^2^** | ***p* value** |
| **Primipara (n=106)** | T1 | -1.86 (-8.20, 4.48) | 0.566 | 0.377 | 0.539 | 3.826 | 0.281 | 5.560 | 0.135 |
|  | T2 | 7.28 (-1.43, 15.99) | 0.102 |  |  |  |  |  |  |
|  | T3 | 1.68 (-6.49, 9.84) | 0.687 |  |  |  |  |  |  |
|  | T4 | 0.98 (-8.01, 9.97) | 0.831 |  |  |  |  |  |  |
| **Multipara (n=55)** | T1 | 8.63 (-1.27, 18.53) | 0.087 | 3.741 | 0.053 | 9.791 | **0.020** | 3.833 | 0.280 |
|  | T2 | 7.43 (-3.66, 18.51) | 0.189 |  |  |  |  |  |  |
|  | T3 | **14.62 (3.81, 25.44)** | **0.008** |  |  |  |  |  |  |
|  | T4 | 3.61 (-8.99, 16.22) | 0.574 |  |  |  |  |  |  |


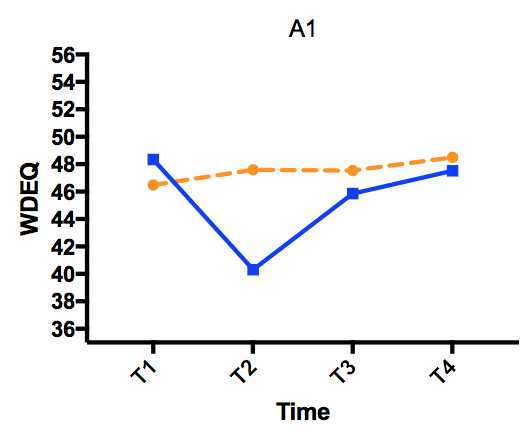

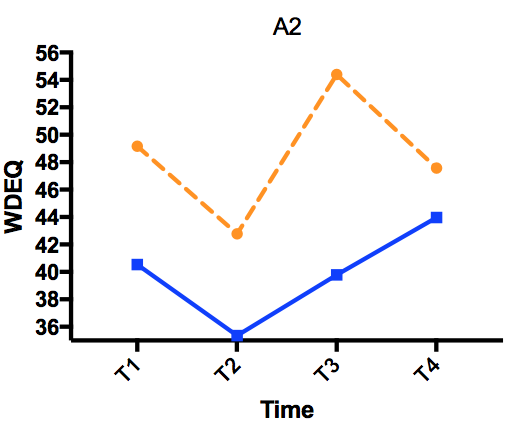

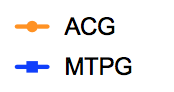


**Figure S18. Longer-term intervention effect on WDEQ by parity**

Note. A1. Model in primipara; A2. Model in multipara; * means significant between-group mean difference.
