# Supplementary material for: Effectiveness of Smartphone-Based Mindfulness Training on Maternal Perinatal Depression: Randomized Controlled Trial
Source: J Med Internet Res. 2021 Jan 27;23(1):e23410. doi: 10.2196/23410 (PMC7875700; doi:10.2196/23410)
Supplement: Multimedia Appendix 8 [file jmir_v23i1e23410_app8.doc]

# **Sensitivity analysis: Adjusted GEE model**

1. **EPDS**

**Table S23. Longer-term effect intervention effect on EPDS based on adjusted** GEE model.

|  |  | **MD _ACG-MTPG_** | ***p* value** | **Group effect** | | **Time effect** | | **Group × Time effect** | |
| --- | --- | --- | --- | --- | --- | --- | --- | --- | --- |
|  |  |  |  | **Wald *χ*^2^** | ***p* value** | **Wald *χ*^2^** | ***p* value** | **Wald *χ*^2^** | ***p* value** |
| **ITT (n=164)** | T1 | 0.55 (-0.74, 1.85) | 0.403 | 1.178 | 0.278 | 14.095 | **0.007** | 15.484 | **0.004** |
|  | T2 | -0.02 (-1.81, 1.84) | 0.985 |  |  |  |  |  |  |
|  | T3 | **2.64 (0.72, 4.56)** | **0.007** |  |  |  |  |  |  |
|  | T4 | 1.63 (-0.40, 3.66) | 0.115 |  |  |  |  |  |  |
|  | T5 | -1.24 (-3.16, 0.68) | 0.207 |  |  |  |  |  |  |
| **PP (n=164)** | T1 | 0.03 (-1.26, 1.31) | 0.966 | 0.279 | 0.597 | 12.399 | **0.015** | 15.530 | **0.004** |
|  | T2 | -0.59 (-2.41, 1.23) | 0.526 |  |  |  |  |  |  |
|  | T3 | **2.27 (0.39, 4.14)** | **0.018** |  |  |  |  |  |  |
|  | T4 | 1.29 (-0.72, 3.30) | 0.208 |  |  |  |  |  |  |
|  | T5 | -1.26 (-3.17, 0.65) | 0.195 |  |  |  |  |  |  |
| **PP-IC (n=135)** | T1 | 0.30 (-1.19, 1.79) | 0.693 | 0.989 | 0.320 | 13.635 | **0.009** | 14.045 | **0.007** |
|  | T2 | 0.24 (-1.50, 1.97) | 0.790 |  |  |  |  |  |  |
|  | T3 | **2.68 (0.77, 4.59)** | **0.006** |  |  |  |  |  |  |
|  | T4 | 1.44 (-0.88, 3.75) | 0.225 |  |  |  |  |  |  |
|  | T5 | -1.03 (-3.27, 1.22) | 0.369 |  |  |  |  |  |  |

Note. intended pregnancy was adjusted.


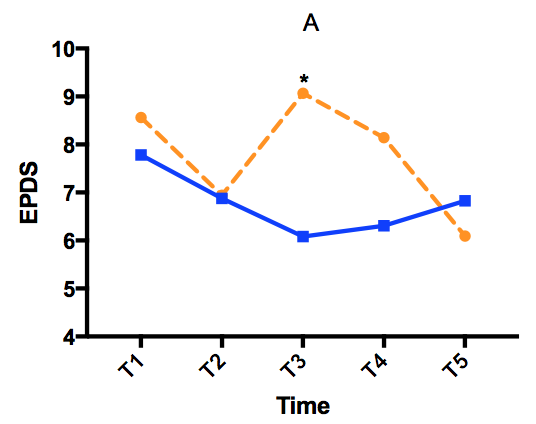

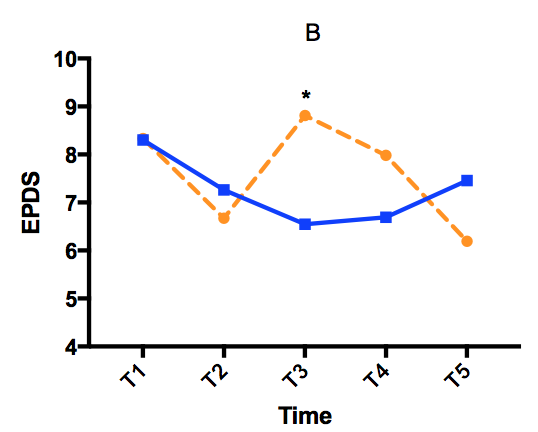

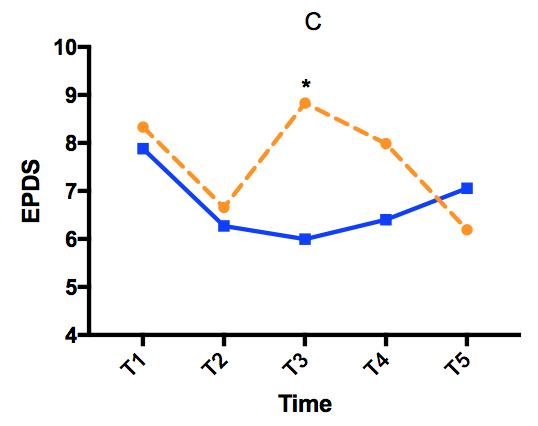

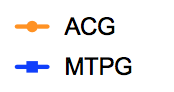


**Figure S19. Longer-term intervention effect on EPDS based on adjusted GEE model**

Note. A. based on ITT; B. based on PP; C. based on PP-IC; * means significant between-group mean difference.

1. **GAD-7**

**Table S24. Longer-term effect intervention effect on GAD-7** based on adjusted GEE model.

|  |  | **MD _ACG-MTPG_** | ***p* value** | **Group effect** | | **Time effect** | | **Group × Time effect** | |
| --- | --- | --- | --- | --- | --- | --- | --- | --- | --- |
|  |  |  |  | **Wald *χ*^2^** | ***p* value** | **Wald *χ*^2^** | ***p* value** | **Wald *χ*^2^** | ***p* value** |
| **ITT (n=162)** | T1 | -0.87 (-1.93, 0.19) | 0.107 | 0.040 | 0.842 | 17.373 | **0.002** | 11.908 | **0.018** |
|  | T2 | -0.47 (-1.74, 0.79) | 0.462 |  |  |  |  |  |  |
|  | T3 | 0.96 (-0.53, 2.44) | 0.206 |  |  |  |  |  |  |
|  | T4 | 1.31 (-0.10, 2.72) | 0.069 |  |  |  |  |  |  |
|  | T5 | -0.46 (-1.91, 0.99) | 0.535 |  |  |  |  |  |  |
| **PP (n=162)** | T1 | **-1.20 (-2.27, -0.12)** | **0.029** | 0.240 | 0.624 | 18.116 | **0.001** | 12.308 | **0.015** |
|  | T2 | -0.87 (-2.15, 0.40) | 0.180 |  |  |  |  |  |  |
|  | T3 | 0.64 (-0.79, 2.06) | 0.381 |  |  |  |  |  |  |
|  | T4 | 1.02 (-0.38, 2.42) | 0.153 |  |  |  |  |  |  |
|  | T5 | -0.70 (-2.11, 0.70) | 0.327 |  |  |  |  |  |  |
| **PP-IC (n=133)** | T1 | -0.71 (-1.92, 0.50) | 0.247 | 0.010 | 0.921 | 19.598 | **0.001** | 7.123 | 0.130 |
|  | T2 | -0.38 (-1.50, 0.75) | 0.512 |  |  |  |  |  |  |
|  | T3 | 0.58 (-0.95, 2.10) | 0.46 |  |  |  |  |  |  |
|  | T4 | 1.10 (-0.36, 2.57) | 0.140 |  |  |  |  |  |  |
|  | T5 | -0.35 (-1.88, 1.04) | 0.572 |  |  |  |  |  |  |

Note. intended pregnancy was adjusted.


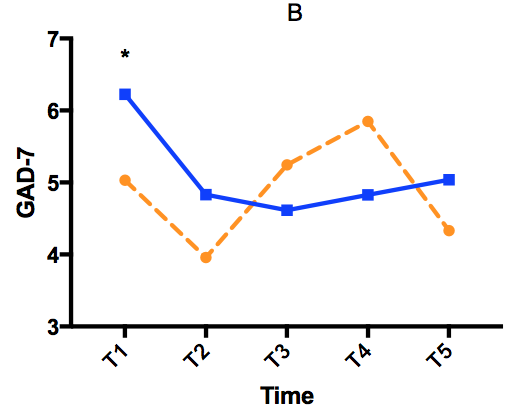

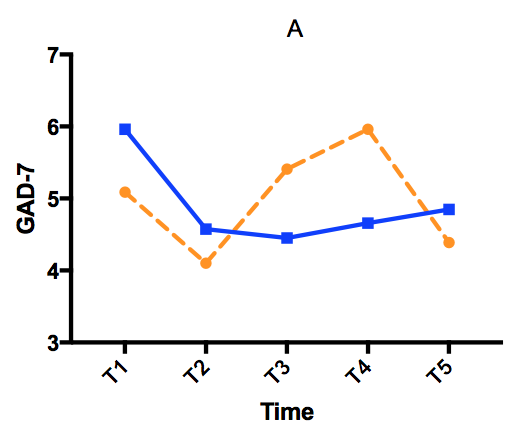

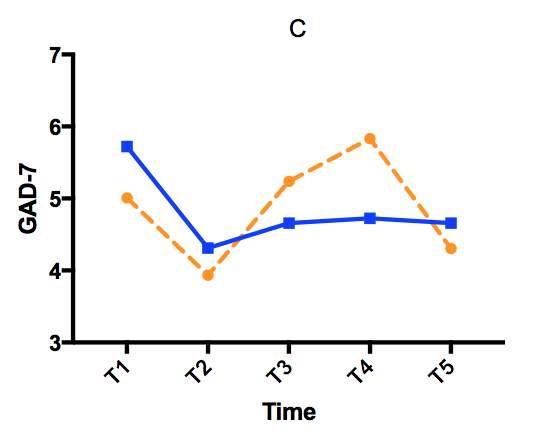

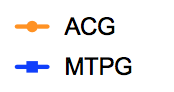


**Figure S20. Longer-term intervention effect on GAD-7 based on adjusted GEE model**

Note. A. based on ITT; B. based on PP; C. based on PP-IC; * means significant between-group mean difference.

1. **PSS**

**Table S25. Longer-term effect intervention effect on PS**S based on adjusted GEE model.

|  |  | **MD _ACG-MTPG_** | ***p* value** | **Group effect** | | **Time effect** | | **Group × Time effect** | |
| --- | --- | --- | --- | --- | --- | --- | --- | --- | --- |
|  |  |  |  | **Wald *χ*^2^** | ***p* value** | **Wald *χ*^2^** | ***p* value** | **Wald *χ*^2^** | ***p* value** |
| **ITT (n=162)** | T1 | 0.15 (-0.61, 0.91) | 0.697 | 1.097 | 0.295 | 11.155 | **0.025** | 2.752 | 0.600 |
|  | T2 | 0.26 (-0.70, 1.23) | 0.591 |  |  |  |  |  |  |
|  | T3 | 0.69 (-0.44, 1.82) | 0.231 |  |  |  |  |  |  |
|  | T4 | 0.79 (-0.48, 2.06) | 0.221 |  |  |  |  |  |  |
|  | T5 | -0.10 (-0.98, 0.78) | 0.829 |  |  |  |  |  |  |
| **PP (n=162)** | T1 | 0.23 (-0.55, 1.01) | 0.562 | 0.561 | 0.454 | 11.396 | **0.022** | 1.651 | 0.800 |
|  | T2 | -0.01 (-0.99, 0.97) | 0.977 |  |  |  |  |  |  |
|  | T3 | 0.48 (-0.63, 1.58) | 0.398 |  |  |  |  |  |  |
|  | T4 | 0.64 (-0.62, 1.90) | 0.317 |  |  |  |  |  |  |
|  | T5 | -0.05 (-0.92, 0.83) | 0.920 |  |  |  |  |  |  |
| **PP-IC (n=133)** | T1 | 0.45 (-0.35, 1.25) | 0.272 | 0.536 | 0.464 | 11.724 | **0.020** | 2.099 | 0.718 |
|  | T2 | -0.02 (-1.01, 0.98) | 0.973 |  |  |  |  |  |  |
|  | T3 | 0.34 (-0.85, 1.53) | 0.573 |  |  |  |  |  |  |
|  | T4 | 0.72 (-0.65, 2.08) | 0.303 |  |  |  |  |  |  |
|  | T5 | -0.07 (-1.12, 0.97) | 0.891 |  |  |  |  |  |  |

Note. intended pregnancy was adjusted.


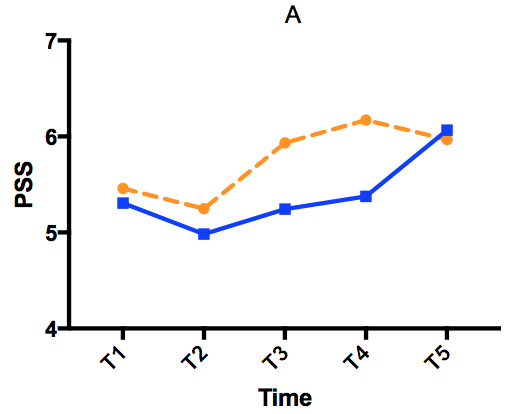

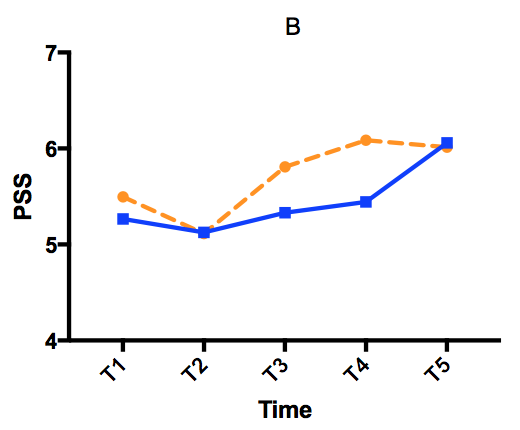

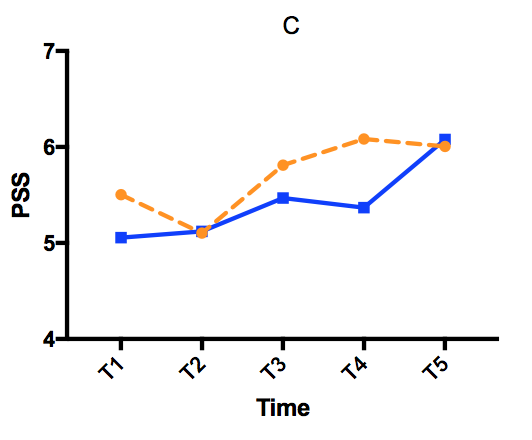

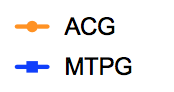


**Figure S21. Longer-term intervention effect on PSS based on adjusted GEE model**

Note. A. based on ITT; B. based on PP; C. based on PP-IC; * means significant between-group mean difference.

1. **PA**

**Table S26. Longer-term effect intervention effect on PA** based on adjusted GEE model.

|  |  | **MD _ACG-MTPG_** | ***p* value** | **Group effect** | | **Time effect** | | **Group × Time effect** | |
| --- | --- | --- | --- | --- | --- | --- | --- | --- | --- |
|  |  |  |  | **Wald *χ*^2^** | ***p* value** | **Wald *χ*^2^** | ***p* value** | **Wald *χ*^2^** | ***p* value** |
| **ITT (n=156)** | T1 | -0.80 (-2.56, 0.97) | 0.377 | 2.121 | 0.145 | 8.446 | **0.038** | 8.415 | **0.038** |
|  | T3 | **-3.04 (-5.47, -0.62)** | **0.014** |  |  |  |  |  |  |
|  | T4 | -2.69 (-5.62, 0.24) | 0.072 |  |  |  |  |  |  |
|  | T5 | 1.61 (-1.30, 4.52) | 0.278 |  |  |  |  |  |  |
| **PP (n=156)** | T1 | -0.08 (-1.86, 1.70) | 0.932 | 0.867 | 0.352 | 7.407 | 0.060 | 7.947 | **0.047** |
|  | T3 | **-2.60 (-4.98, -0.21)** | **0.033** |  |  |  |  |  |  |
|  | T4 | -2.18 (-5.11, 0.75) | 0.145 |  |  |  |  |  |  |
|  | T5 | 1.70 (-1.14, 4.54) | 0.241 |  |  |  |  |  |  |
| **PP-IC (n=129)** | T1 | 0.64 (-1.50, 2.77) | 0.559 | 0.299 | 0.584 | 5.240 | 0.155 | 11.445 | **0.010** |
|  | T3 | **-2.84 (-5.41, -0.26)** | **0.031** |  |  |  |  |  |  |
|  | T4 | -2.37 (-5.55, 0.81) | 0.144 |  |  |  |  |  |  |
|  | T5 | 2.35 (-0.88, 5.58) | 0.154 |  |  |  |  |  |  |

Note. intended pregnancy was adjusted.


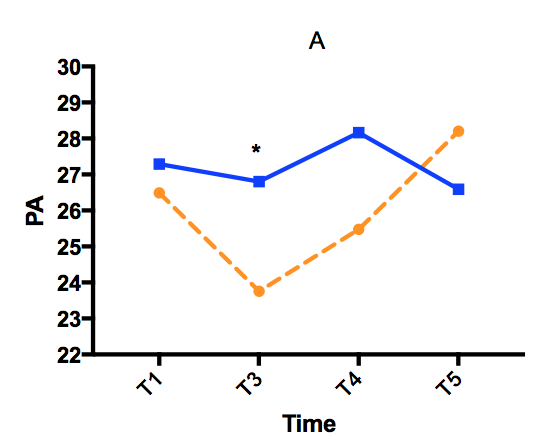

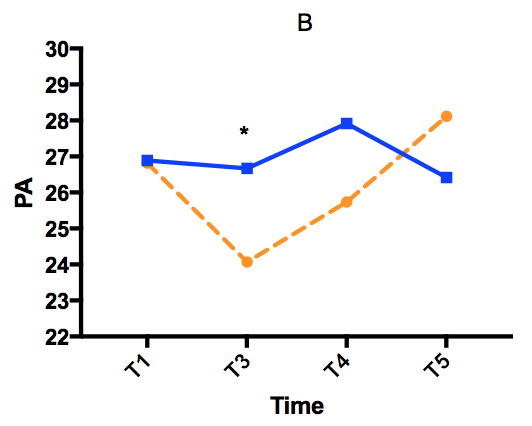

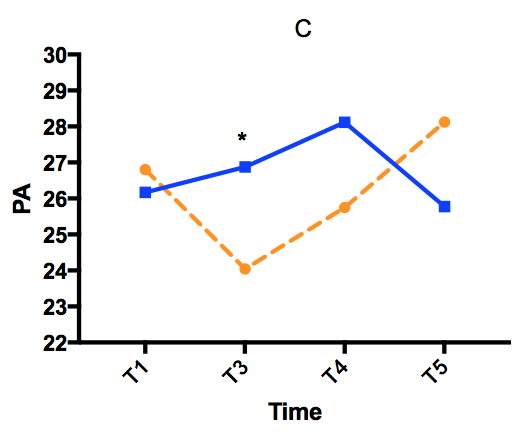

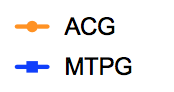


**Figure S22. Longer-term intervention effect on PA based on adjusted GEE model**

Note. A. based on ITT; B. based on PP; C. based on PP-IC; * means significant between-group mean difference.

1. **NA**

**Table S27. Longer-term effect intervention effect on log-transformed NA** based on adjusted GEE model.

|  |  | **MD _ACG-MTPG_** | ***p* value** | **Group effect** | | **Time effect** | | **Group × Time effect** | |
| --- | --- | --- | --- | --- | --- | --- | --- | --- | --- |
|  |  |  |  | **Wald *χ*^2^** | ***p* value** | **Wald *χ*^2^** | ***p* value** | **Wald *χ*^2^** | ***p* value** |
| **ITT (n=156)** | T1 | -0.03 (-0.07, 0.01) | 0.145 | 0.000 | 0.998 | 18.002 | **<0.001** | 2.719 | 0.437 |
|  | T3 | 0.00 (-0.05, 0.06) | 0.930 |  |  |  |  |  |  |
|  | T4 | 0.02 (-0.04, 0.08) | 0.539 |  |  |  |  |  |  |
|  | T5 | 0.01 (-0.05, 0.06) | 0.821 |  |  |  |  |  |  |
| **PP (n=156)** | T1 | -0.03 (-0.07, 0.01) | 0.104 | 0.008 | 0.930 | 18.564 | **<0.001** | 4.009 | 0.260 |
|  | T3 | -0.01 (-0.06, 0.05) | 0.761 |  |  |  |  |  |  |
|  | T4 | 0.03 (-0.03, 0.09) | 0.388 |  |  |  |  |  |  |
|  | T5 | 0.01 (-0.05, 0.06) | 0.793 |  |  |  |  |  |  |
| **PP-IC (n=129)** | T1 | 0.01 (-0.04, 0.05) | 0.686 | 0.045 | 0.833 | 7.632 | 0.054 | 4.693 | 0.196 |
|  | T3 | 0.03 (-0.04, 0.09) | 0.415 |  |  |  |  |  |  |
|  | T4 | -0.04 (-0.10, 0.03) | 0.230 |  |  |  |  |  |  |
|  | T5 | -0.01 (-0.08, 0.05) | 0.678 |  |  |  |  |  |  |

Note. intended pregnancy was adjusted.


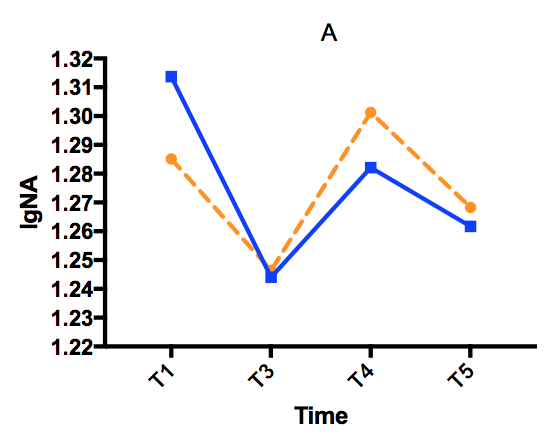

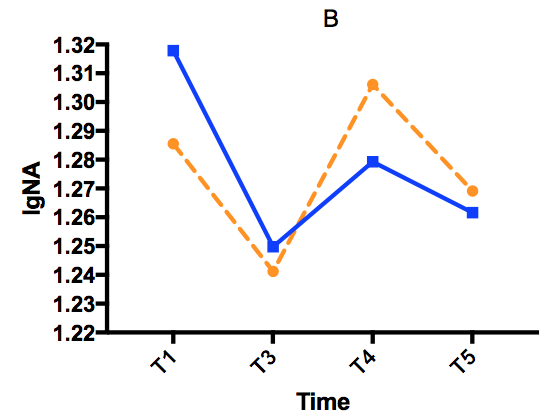

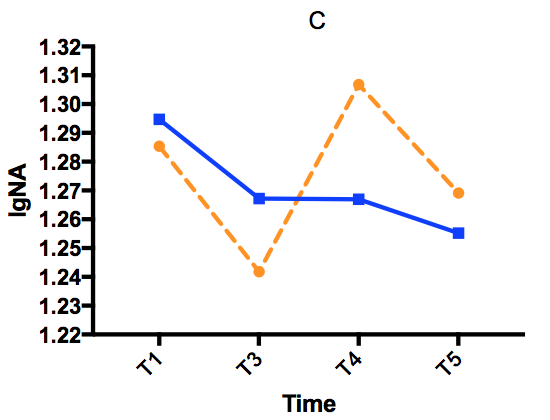

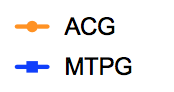


**Figure S23. Longer-term intervention effect on log-transformed NA based on adjusted GEE model**

Note. A. based on ITT; B. based on PP; C. based on PP-IC; * means significant between-group mean difference.

1. **PSQI**

**Table S28. Longer-term effect intervention effect on PSQI** based on adjusted GEE model.

|  |  | **MD _ACG-MTPG_** | ***p* value** | **Group effect** | | **Time effect** | | **Group × Time effect** | |
| --- | --- | --- | --- | --- | --- | --- | --- | --- | --- |
|  |  |  |  | **Wald *χ*^2^** | ***p* value** | **Wald *χ*^2^** | ***p* value** | **Wald *χ*^2^** | ***p* value** |
| **ITT (n=156)** | T1 | -0.03 (-0.09, 0.03) | 0.297 | 0.229 | 0.632 | 16.630 | **0.001** | 2.256 | 0.521 |
|  | T3 | 0.03 (-0.06, 0.11) | 0.547 |  |  |  |  |  |  |
|  | T4 | -0.00 (-0.09, 0.09) | 0.947 |  |  |  |  |  |  |
|  | T5 | -0.05 (-0.15, 0.05) | 0.353 |  |  |  |  |  |  |
| **PP (n=156)** | T1 | -0.04 (-0.10, 0.02) | 0.155 | 1.106 | 0.293 | 16.821 | **0.001** | 2.557 | 0.465 |
|  | T3 | 0.01 (-0.08, 0.10) | 0.839 |  |  |  |  |  |  |
|  | T4 | -0.01 (-0.10, 0.07) | 0.743 |  |  |  |  |  |  |
|  | T5 | -0.08 (-0.18, 0.02) | 0.137 |  |  |  |  |  |  |
| **PP-IC (n=128)** | T1 | 0.02 (-0.04, 0.09) | 0.462 | 0.077 | 0.782 | 14.941 | **0.002** | 2.276 | 0.517 |
|  | T3 | -0.02(-0.11, 0.07) | 0.635 |  |  |  |  |  |  |
|  | T4 | -0.02 (-0.13, 0.08) | 0.686 |  |  |  |  |  |  |
|  | T5 | 0.06 (-0.07, 0.18) | 0.363 |  |  |  |  |  |  |

Note. intended pregnancy was adjusted.


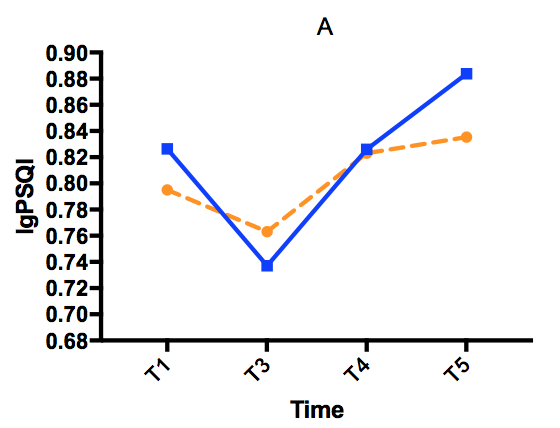

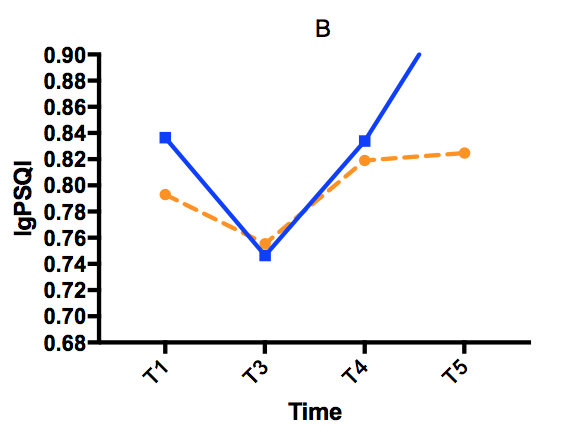

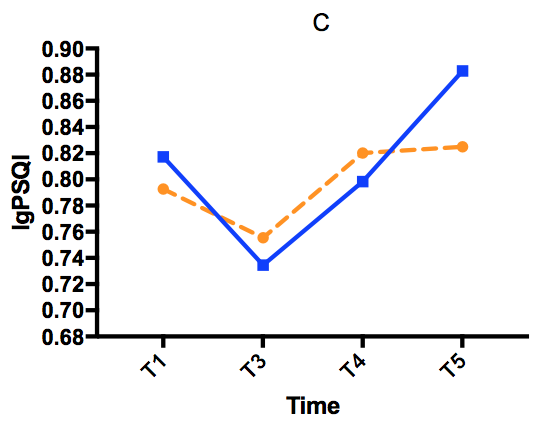

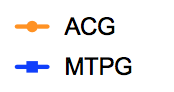


**Figure S24. Longer-term intervention effect on log-transformed PSQI based on adjusted GEE model**

Note. A. based on ITT; B. based on PP; C. based on PP-IC; * means significant between-group mean difference.

1. **FSS**

**Table S29. Longer-term effect intervention effect on FS**S based on adjusted GEE model.

|  |  | **MD _ACG-MTPG_** | ***p* value** | **Group effect** | | **Time effect** | | **Group × Time effect** | |
| --- | --- | --- | --- | --- | --- | --- | --- | --- | --- |
|  |  |  |  | **Wald *χ*^2^** | ***p* value** | **Wald *χ*^2^** | ***p* value** | **Wald *χ*^2^** | ***p* value** |
| **ITT (n=157)** | T1 | 0.53 (-2.40, 3.45) | 0.724 | 0.550 | 0.458 | 15.296 | **0.002** | 1.945 | 0.584 |
|  | T3 | 0.82 (-3.18, 4.82) | 0.688 |  |  |  |  |  |  |
|  | T4 | 2.89 (-1.18, 6.97) | 0.164 |  |  |  |  |  |  |
|  | T5 | -0.12 (-4.73, 4.49) | 0.960 |  |  |  |  |  |  |
| **PP (n=157)** | T1 | 1.10 (-1.81, 4.01) | 0.458 | 0.133 | 0.716 | 14.948 | **0.002** | 2.956 | 0.398 |
|  | T3 | 0.20 (-3.69, 4.09) | 0.919 |  |  |  |  |  |  |
|  | T4 | 2.45 (-1.61, 6.50) | 0.236 |  |  |  |  |  |  |
|  | T5 | -1.75 (-6.38, 2.87) | 0.458 |  |  |  |  |  |  |
| **PP-IC (n=130)** | T1 | 2.51 (-0.97, 5.99) | 0.158 | 0.488 | 0.485 | 7.271 | 0.064 | 3.132 | 0.372 |
|  | T3 | 0.48(-3.83, 4.79) | 0.827 |  |  |  |  |  |  |
|  | T4 | 3.16 (-1.38, 7.69) | 0.172 |  |  |  |  |  |  |
|  | T5 | -1.52 (-7.22, 4.17) | 0.601 |  |  |  |  |  |  |

Note. intended pregnancy was adjusted.


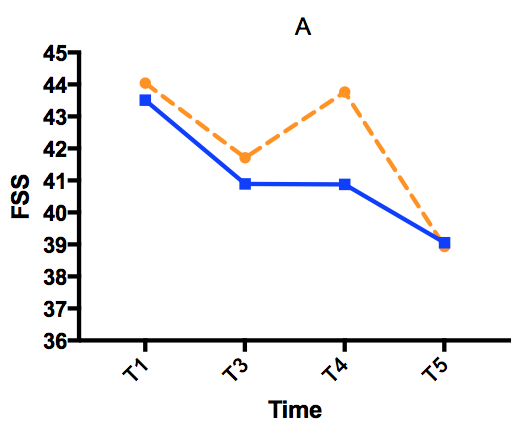

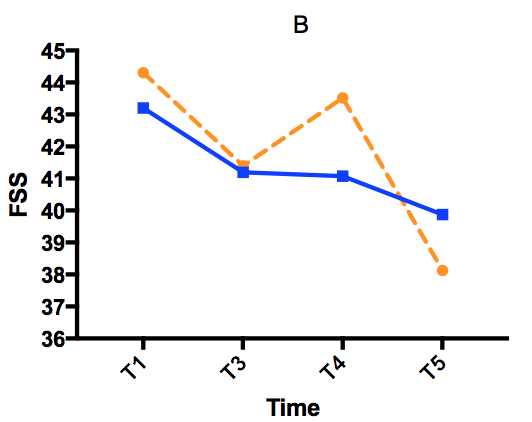

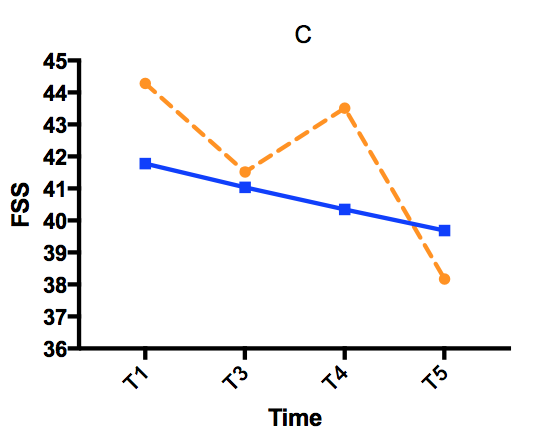

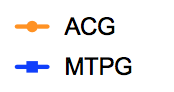


**Figure S25. Longer-term intervention effect on FSS based on adjusted GEE model**

Note. A. based on ITT; B. based on PP; C. based on PP-IC; * means significant between-group mean difference

1. **PM**

**Table S30. Longer-term effect intervention effect on log-transformed PM based on adjusted** GEE model.

|  |  | **MD _ACG-MTPG_** | ***p* value** | **Group effect** | | **Time effect** | | **Group × Time effect** | |
| --- | --- | --- | --- | --- | --- | --- | --- | --- | --- |
|  |  |  |  | **Wald *χ*^2^** | ***p* value** | **Wald *χ*^2^** | ***p* value** | **Wald *χ*^2^** | ***p* value** |
| **ITT (n=156)** | T1 | 0.01 (-0.03, 0.05) | 0.683 | 0.845 | 0.358 | 2.158 | 0.340 | 5.021 | 0.081 |
|  | T3 | -0.04 (-0.10, 0.01) | 0.107 |  |  |  |  |  |  |
|  | T5 | -0.03 (-0.10, 0.05) | 0.482 |  |  |  |  |  |  |
| **PP (n=156)** | T1 | 0.01 (-0.04, 0.05) | 0.802 | 1.266 | 0.261 | 2.672 | 0.263 | 4.012 | 0.134 |
|  | T3 | -0.04 (-0.09, 0.02) | 0.185 |  |  |  |  |  |  |
|  | T5 | -0.05 (-0.12, 0.03) | 0.225 |  |  |  |  |  |  |
| **PP-IC (n=128)** | T1 | 0.01 (-0.04, 0.06) | 0.646 | 1.081 | 0.298 | 4.032 | 0.133 | 5.322 | 0.070 |
|  | T3 | -0.04 (-0.10, 0.02) | 0.166 |  |  |  |  |  |  |
|  | T5 | -0.05 (-0.13, 0.03) | 0.231 |  |  |  |  |  |  |

Note. intended pregnancy was adjusted.


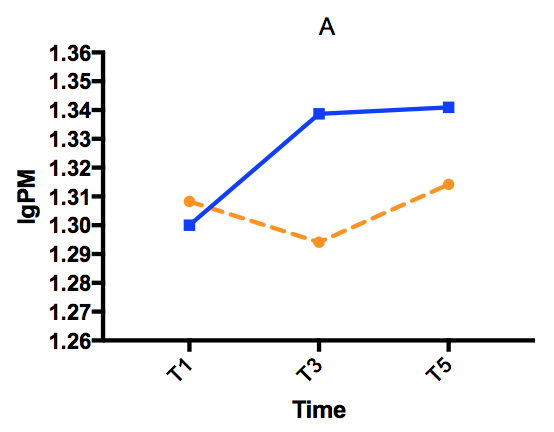

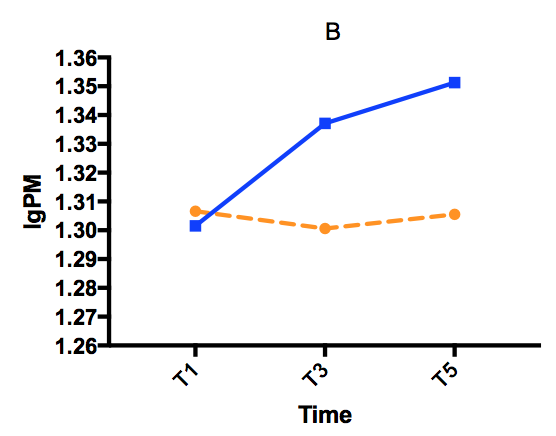

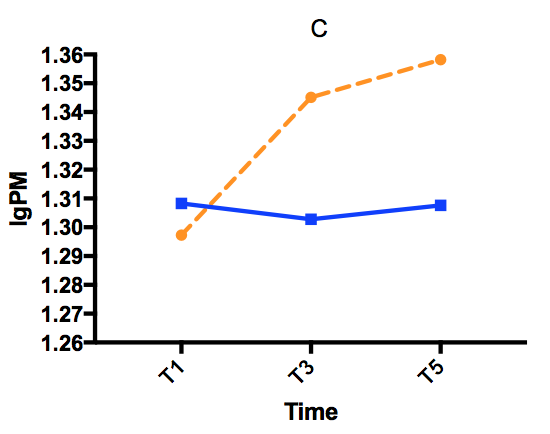

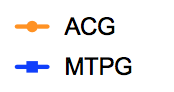


**Figure S26. Longer-term intervention effect on log-transformed PM based on adjusted GEE model**

Note. A. based on ITT; B. based on PP; C. based on PP-IC; * means significant between-group mean difference

1. **RM**

**Table S31. Longer-term effect intervention effect on RM based on adjusted** GEE model.

|  |  | **MD _ACG-MTPG_** | ***p* value** | **Group effect** | | **Time effect** | | **Group × Time effect** | |
| --- | --- | --- | --- | --- | --- | --- | --- | --- | --- |
|  |  |  |  | **Wald *χ*^2^** | ***p* value** | **Wald *χ*^2^** | ***p* value** | **Wald *χ*^2^** | ***p* value** |
| **ITT (n=156)** | T1 | 0.85 (-1.05, 2.75) | 0.381 | 0.171 | 0.679 | 13.363 | **0.001** | 4.705 | 0.095 |
|  | T3 | -1.29 (-3.87, 1.29) | 0.327 |  |  |  |  |  |  |
|  | T5 | -0.87 (-4.11, 2.37) | 0.600 |  |  |  |  |  |  |
| **PP (n=156)** | T1 | 1.01 (-0.90, 2.93) | 0.300 | 0.159 | 0.690 | 14.485 | **0.001** | 4.844 | 0.089 |
|  | T3 | -0.54 (-3.14, 2.06) | 0.684 |  |  |  |  |  |  |
|  | T5 | -1.74 (-4.98, 1.50) | 0.292 |  |  |  |  |  |  |
| **PP-IC (n=128)** | T1 | -1.31 (-3.53, 0.91) | 0.247 | 0.202 | 0.653 | 17.746 | <0.001 | 7.339 | **0.025** |
|  | T3 | 0.96 (-1.90, 3.82) | 0.510 |  |  |  |  |  |  |
|  | T5 | 1.98 (-1.67, 5.64) | 0.288 |  |  |  |  |  |  |

Note. intended pregnancy was adjusted.


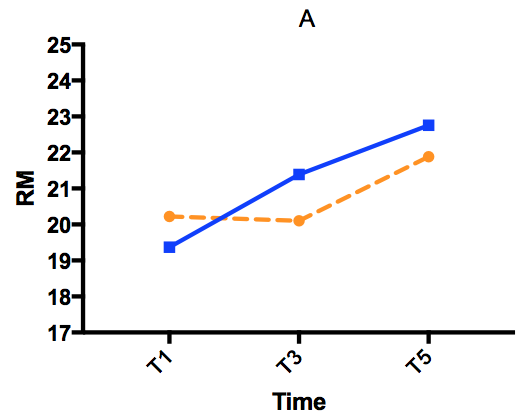

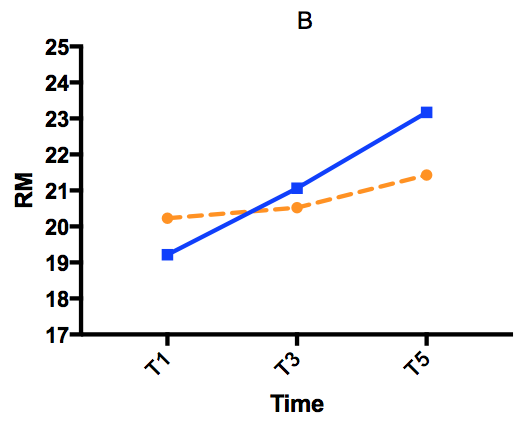

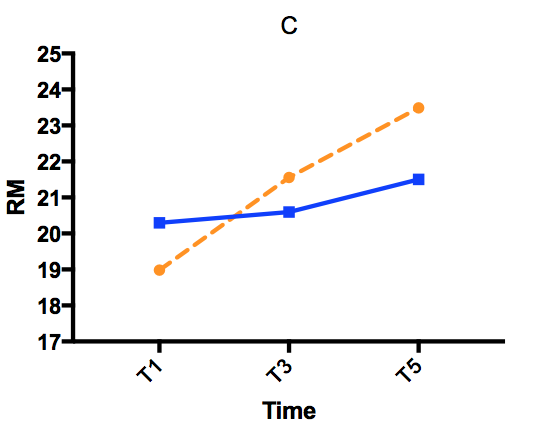

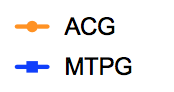


**Figure S27. Longer-term intervention effect on RM based on adjusted GEE model**

Note. A. based on ITT; B. based on PP; C. based on PP-IC; * means significant between-group mean difference

1. **WDEQ**

**Table S32. Longer-term effect intervention effect on WDEQ based on adjusted** GEE model.

|  |  | **MD _ACG-MTPG_** | ***p* value** | **Group effect** | | **Time effect** | | **Group × Time effect** | |
| --- | --- | --- | --- | --- | --- | --- | --- | --- | --- |
|  |  |  |  | **Wald *χ*^2^** | ***p* value** | **Wald *χ*^2^** | ***p* value** | **Wald *χ*^2^** | ***p* value** |
| **ITT (n=157)** | T1 | 0.55 (-4.97, 6.07) | 0.846 | 1.799 | 0.180 | 11.088 | **0.011** | 4.580 | 0.205 |
|  | T2 | 6.58 (-0.30, 13.46) | 0.061 |  |  |  |  |  |  |
|  | T3 | 5.58 (-1.36, 12.52) | 0.115 |  |  |  |  |  |  |
|  | T4 | 2.27 (-5.54, 10.08) | 0.568 |  |  |  |  |  |  |
| **PP (n=157)** | T1 | -0.66 (-6.27, 4.94) | 0.817 | 0.330 | 0.565 | 11.518 | **0.009** | 2.866 | 0.413 |
|  | T2 | 3.50 (-3.57, 10.56) | 0.332 |  |  |  |  |  |  |
|  | T3 | 3.65 (-3.37, 10.68) | 0.308 |  |  |  |  |  |  |
|  | T4 | 0.13 (-7.78, 8.04) | 0.974 |  |  |  |  |  |  |
| **PP-IC (n=130)** | T1 | -0.12 (-6.68, 6.43) | 0.970 | 0.175 | 0.676 | 9.005 | **0.029** | 0.831 | 0.842 |
|  | T2 | -2.39 (-10.58, 5.81) | 0.568 |  |  |  |  |  |  |
|  | T3 | -2.86 (-10.94, 5.22) | 0.488 |  |  |  |  |  |  |
|  | T4 | -0.35 (-9.47, 8.77) | 0.940 |  |  |  |  |  |  |

Note. intended pregnancy was adjusted.


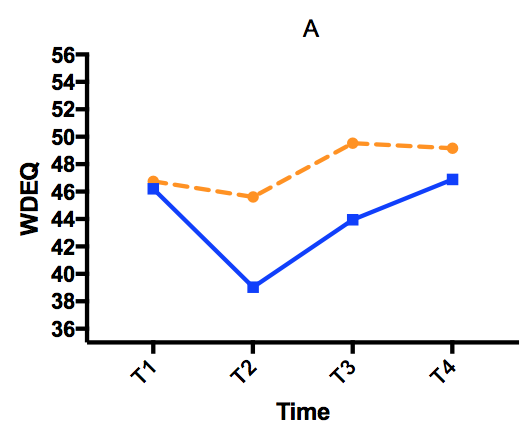

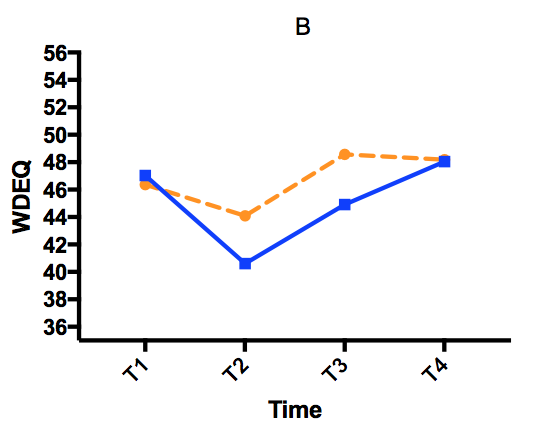

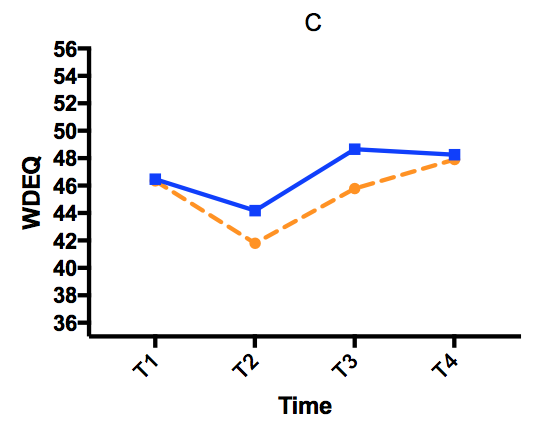

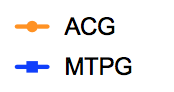


**Figure S28. Longer-term intervention effect on WDEQ based on adjusted GEE model**

Note. A. based on ITT; B. based on PP; C. based on PP-IC; * means significant between-group mean difference
