# Supplementary material for: Effectiveness of Smartphone-Based Mindfulness Training on Maternal Perinatal Depression: Randomized Controlled Trial
Source: J Med Internet Res. 2021 Jan 27;23(1):e23410. doi: 10.2196/23410 (PMC7875700; doi:10.2196/23410)
Supplement: Multimedia Appendix 9 [file jmir_v23i1e23410_app9.doc]

# **Sensitivity analysis: participants completed different number of follow-up**

1. **EPDS**

**Table S33. Longer-term effect intervention effect on EPDS in** participants completed different times of follow-ups.

|  |  | **MD _ACG-MTPG_** | ***p* value** | **Group effect** | | **Time effect** | | **Group × Time effect** | |
| --- | --- | --- | --- | --- | --- | --- | --- | --- | --- |
|  |  |  |  | **Wald *χ*^2^** | ***p* value** | **Wald *χ*^2^** | ***p* value** | **Wald *χ*^2^** | ***p* value** |
| **Complete at least 2 follow-ups (n=113)** | T1 | 1.21 (-0.41, 2.82) | 0.144 | 2.833 | 0.092 | 15.542 | **0.004** | 20.625 | **<0.001** |
|  | T2 | 0.50 (-1.43, 2.44) | 0.611 |  |  |  |  |  |  |
|  | T3 | **3.55 (1.54, 5.56)** | **0.001** |  |  |  |  |  |  |
|  | T4 | 1.79 (-0.30, 3.89) | 0.094 |  |  |  |  |  |  |
|  | T5 | -0.84 (-2.79, 1.11) | 0.400 |  |  |  |  |  |  |
| **Complete at least 3 follow-ups (n=94)** | T1 | 1.09 (-0.70, 2.87) | 0.233 | 3.265 | 0.071 | 12.983 | **0.011** | 18.773 | **0.001** |
|  | T2 | 0.91 (-1.25, 3.06) | 0.409 |  |  |  |  |  |  |
|  | T3 | **3.90 (1.63, 6.17)** | **0.001** |  |  |  |  |  |  |
|  | T4 | 2.11 (-0.12, 4.34) | 0.064 |  |  |  |  |  |  |
|  | T5 | -0.50 (-2.58, 1.58) | 0.638 |  |  |  |  |  |  |
| **Complete all 4 follow-ups (n=56)** | T1 | 0.72 (-1.37, 2.81) | 0.500 | 2.552 | 0.110 | 11.050 | **0.026** | 8.105 | 0.088 |
|  | T2 | 1.22 (-1.39, 3.83) | 0.360 |  |  |  |  |  |  |
|  | T3 | **3.03 (0.24, 5.82)** | **0.033** |  |  |  |  |  |  |
|  | T4 | **3.26 (0.49, 6.03)** | **0.021** |  |  |  |  |  |  |
|  | T5 | -0.24 (-2.80, 2.32) | 0.854 |  |  |  |  |  |  |


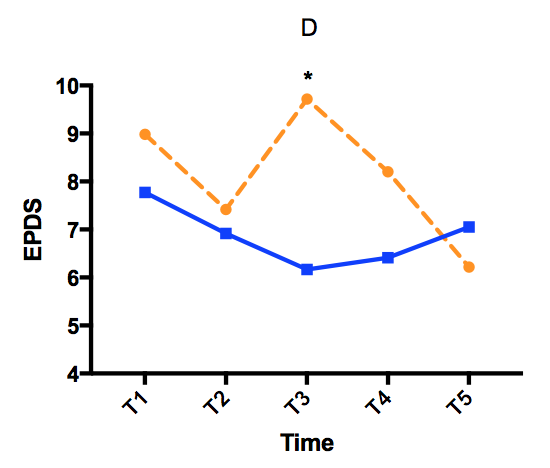

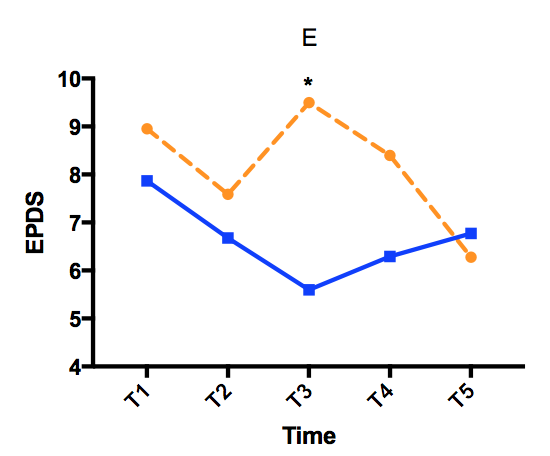

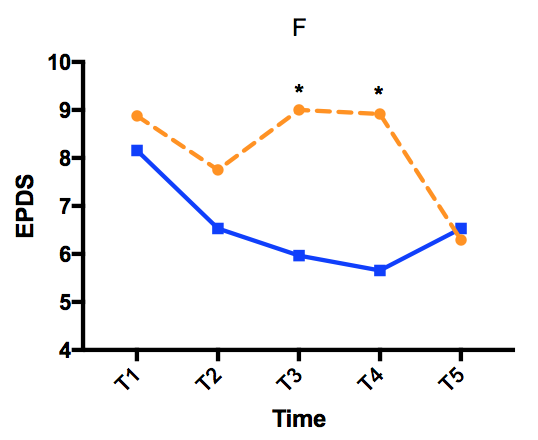

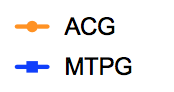


**Figure S29. Longer-term intervention effect on EPDS in participants completed different times of follow-ups**

Note. D. complete at least 2 follow-ups; E. complete at least 3 follow-ups; F. complete all 4 follow-ups; * means significant between-group mean difference.

1. **GAD-7**

**Table S34. Longer-term effect intervention effect on GAD-7** in participants completed different times of follow-ups.

|  |  | **MD _ACG-MTPG_** | ***p* value** | **Group effect** | | **Time effect** | | **Group × Time effect** | |
| --- | --- | --- | --- | --- | --- | --- | --- | --- | --- |
|  |  |  |  | **Wald *χ*^2^** | ***p* value** | **Wald *χ*^2^** | ***p* value** | **Wald *χ*^2^** | ***p* value** |
| **Complete at least 2 follow-ups (n=113)** | T1 | -1.06 (-2.26, 0.13) | 0.080 | 0.445 | 0.505 | 14.993 | **0.005** | 15.116 | **0.004** |
|  | T2 | -0.15 (-1.41, 1.12) | 0.822 |  |  |  |  |  |  |
|  | T3 | **1.62 (0.01, 3.22)** | **0.049** |  |  |  |  |  |  |
|  | T4 | 1.25 (-0.17, 2.68) | 0.085 |  |  |  |  |  |  |
|  | T5 | -0.06 (-1.53, 1.42) | 0.941 |  |  |  |  |  |  |
| **Complete at least 3 follow-ups (n=94)** | T1 | -0.88 (-2.23, 0.46) | 0.198 | 2.264 | 0.132 | 12.720 | **0.013** | 17.930 | **0.001** |
|  | T2 | 0.32 (-1.09, 1.72) | 0.660 |  |  |  |  |  |  |
|  | T3 | **2.47 (0.66, 4.28)** | **0.007** |  |  |  |  |  |  |
|  | T4 | **1.71 (0.22, 3.19)** | **0.024** |  |  |  |  |  |  |
|  | T5 | 0.45 (-1.10, 2.01) | 0.568 |  |  |  |  |  |  |
| **Complete all 4 follow-ups (n=56)** | T1 | -1.11 (-2.69, 0.47) | 0.169 | 0.560 | 0.454 | 9.322 | 0.054 | 11.940 | **0.018** |
|  | T2 | 0.32 (-1.54, 2.18) | 0.734 |  |  |  |  |  |  |
|  | T3 | 1.41 (-0.78, 3.59) | 0.206 |  |  |  |  |  |  |
|  | T4 | 1.72 (-0.06, 3.50) | 0.058 |  |  |  |  |  |  |
|  | T5 | 0.31 (-1.77, 2.40) | 0.769 |  |  |  |  |  |  |


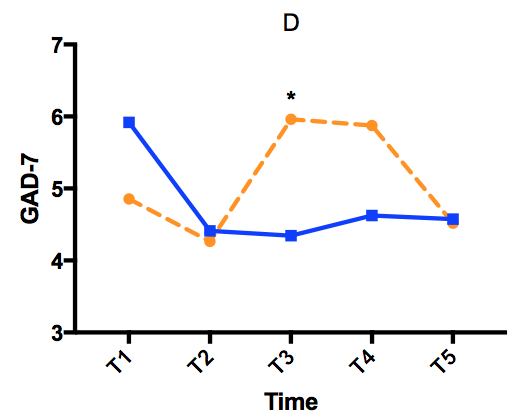

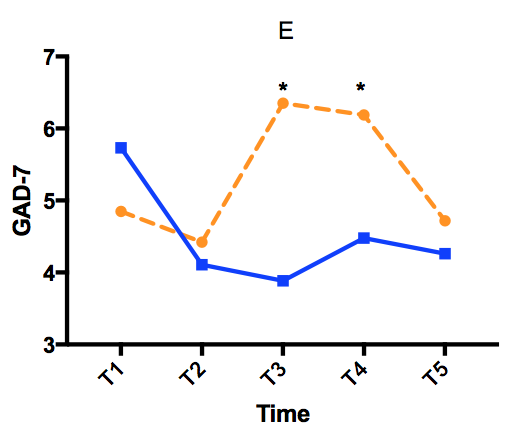

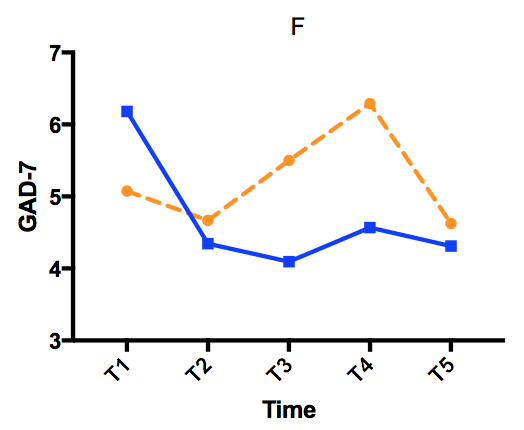

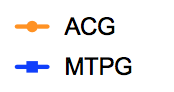


**Figure S30. Longer-term intervention effect on GAD-7 in participants completed different times of follow-ups**

Note. D. complete at least 2 follow-ups; E. complete at least 3 follow-ups; F. complete all 4 follow-ups; * means significant between-group mean difference.

1. **PSS**

**Table S35. Longer-term effect intervention effect on PS**S in participants completed different times of follow-ups.

|  |  | **MD _ACG-MTPG_** | ***p* value** | **Group effect** | | **Time effect** | | **Group × Time effect** | |
| --- | --- | --- | --- | --- | --- | --- | --- | --- | --- |
|  |  |  |  | **Wald *χ*^2^** | ***p* value** | **Wald *χ*^2^** | ***p* value** | **Wald *χ*^2^** | ***p* value** |
| **Complete at least 2 follow-ups (n=113)** | T1 | 0.13 (-0.77, 1.03) | 0.779 | 2.159 | 0.142 | 10.232 | **0.037** | 5.803 | 0.214 |
|  | T2 | 0.41 (-0.64, 1.46) | 0.441 |  |  |  |  |  |  |
|  | T3 | **1.31 (0.14, 2.45)** | **0.029** |  |  |  |  |  |  |
|  | T4 | 0.75 (-0.46, 1.97) | 0.225 |  |  |  |  |  |  |
|  | T5 | -0.05 (-0.93, 0.83) | 0.916 |  |  |  |  |  |  |
| **Complete at least 3 follow-ups (n=94)** | T1 | 0.27 (-0.73, 1.27) | 0.599 | 2.160 | 0.142 | 8.483 | 0.075 | 4.222 | 0.377 |
|  | T2 | 0.61 (-0.55, 1.76) | 0.304 |  |  |  |  |  |  |
|  | T3 | 1.05 (-0.17, 2.27) | 0.093 |  |  |  |  |  |  |
|  | T4 | 0.95 (-0.32, 2.22) | 0.142 |  |  |  |  |  |  |
|  | T5 | -0.02 (-0.95, 0.92) | 0.974 |  |  |  |  |  |  |
| **Complete all 4 follow-ups (n=56)** | T1 | 0.85 (-0.42, 2.12) | 0.188 | 4.632 | **0.031** | 10.803 | **0.029** | 2.538 | 0.638 |
|  | T2 | 1.01 (-0.37, 2.38) | 0.151 |  |  |  |  |  |  |
|  | T3 | **1.43 (0.04, 2.81)** | **0.043** |  |  |  |  |  |  |
|  | T4 | 1.38 (-0.20, 2.95) | 0.088 |  |  |  |  |  |  |
|  | T5 | 0.46 (-0.70, 1.61) | 0.437 |  |  |  |  |  |  |


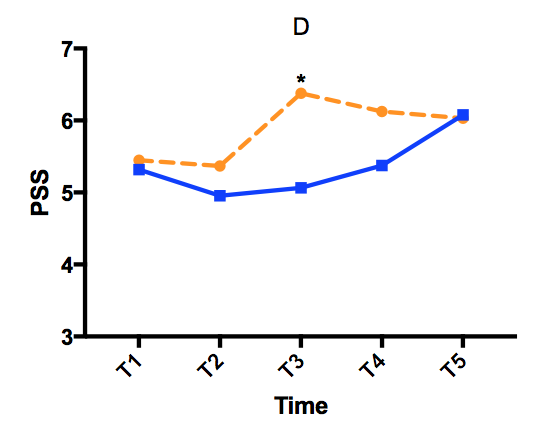

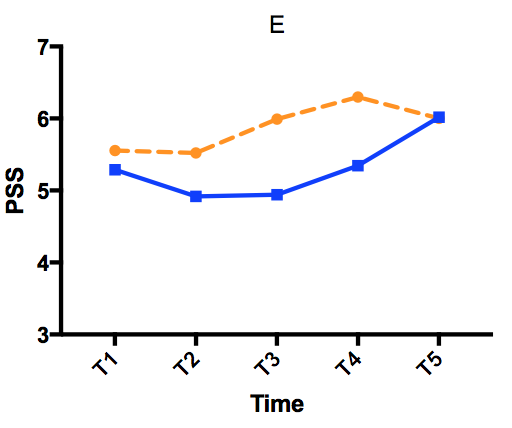

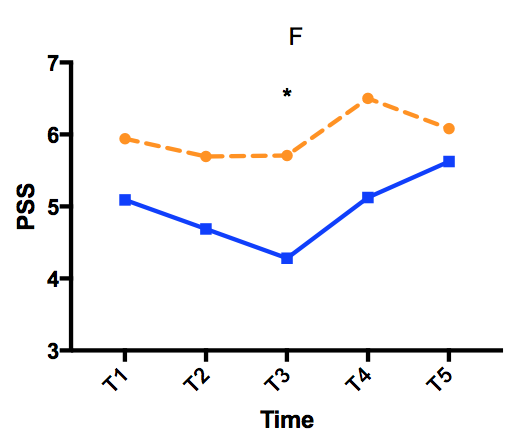

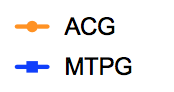


**Figure S31. Longer-term intervention effect on PSS in participants completed different times of follow-ups**

Note. D. complete at least 2 follow-ups; E. complete at least 3 follow-ups; F. complete all 4 follow-ups; * means significant between-group mean difference.

1. **PA**

**Table S36. Longer-term effect intervention effect on PA** in participants completed different times of follow-ups.

|  |  | **MD _ACG-MTPG_** | ***p* value** | **Group effect** | | **Time effect** | | **Group × Time effect** | |
| --- | --- | --- | --- | --- | --- | --- | --- | --- | --- |
|  |  |  |  | **Wald *χ*^2^** | ***p* value** | **Wald *χ*^2^** | ***p* value** | **Wald *χ*^2^** | ***p* value** |
| **Complete at least 2 follow-ups (n=113)** | T1 | 0.29 (-1.83, 2.41) | 0.788 | 1.148 | 0.284 | 6.958 | 0.073 | 10.208 | **0.017** |
|  | T3 | **-3.56 (-6.10, -1.03)** | **0.006** |  |  |  |  |  |  |
|  | T4 | -2.20 (-5.08, 0.68) | 0.134 |  |  |  |  |  |  |
|  | T5 | 1.69 (-1.31, 4.68) | 0.269 |  |  |  |  |  |  |
| **Complete at least 3 follow-ups (n=94)** | T1 | 0.14 (-2.17, 2.45) | 0.905 | 2.188 | 0.139 | 6.852 | 0.077 | 10.508 | 0.015 |
|  | T3 | **-4.21 (-7.00, -1.42)** | **0.003** |  |  |  |  |  |  |
|  | T4 | -2.83 (-5.80, 0.14) | 0.062 |  |  |  |  |  |  |
|  | T5 | 1.38 (-1.69, 4.45) | 0.380 |  |  |  |  |  |  |
| **Complete all 4 follow-ups (n=56)** | T1 | -0.24 (-3.37, 2.88) | 0.878 | 2.526 | 0.112 | 3.550 | 0.314 | 9.884 | **0.020** |
|  | T3 | **-5.73 (-9.05, -2.41)** | **0.001** |  |  |  |  |  |  |
|  | T4 | -2.53 (-5.91, 0.85) | 0.142 |  |  |  |  |  |  |
|  | T5 | 1.42 (-2.55, 5.38) | 0.484 |  |  |  |  |  |  |


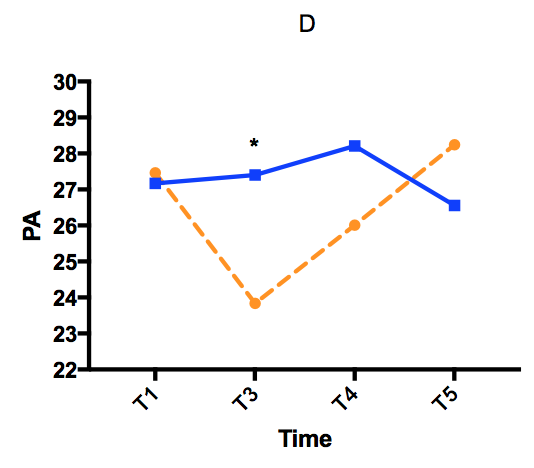

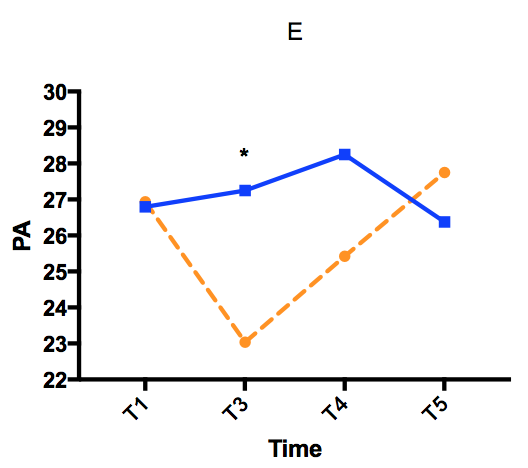

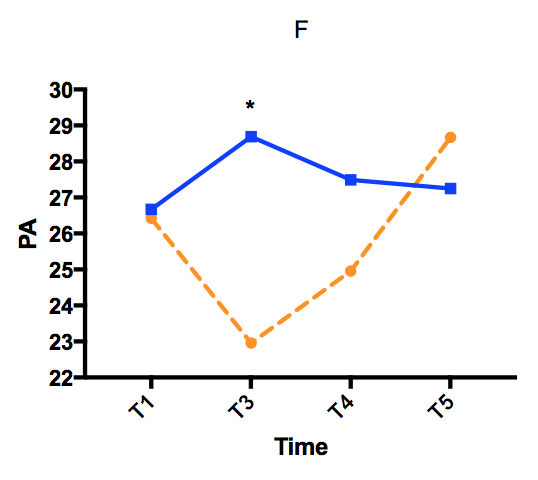

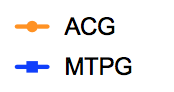


**Figure S32. Longer-term intervention effect on PA in participants completed different times of follow-ups**

Note. D. complete at least 2 follow-ups; E. complete at least 3 follow-ups; F. complete all 4 follow-ups; * means significant between-group mean difference.

1. **NA**

**Table S37. Longer-term effect intervention effect on log-transformed NA** in participants completed different times of follow-ups.

|  |  | **MD _ACG-MTPG_** | ***p* value** | **Group effect** | | **Time effect** | | **Group × Time effect** | |
| --- | --- | --- | --- | --- | --- | --- | --- | --- | --- |
|  |  |  |  | **Wald *χ*^2^** | ***p* value** | **Wald *χ*^2^** | ***p* value** | **Wald *χ*^2^** | ***p* value** |
| **Complete at least 2 follow-ups (n=113)** | T1 | -0.03 (-0.08, 0.01) | 0.163 | 0.000 | 0.989 | 13.351 | **0.004** | 3.065 | 0.382 |
|  | T3 | 0.01 (-0.04, 0.07) | 0.604 |  |  |  |  |  |  |
|  | T4 | 0.01 (-0.05, 0.07) | 0.768 |  |  |  |  |  |  |
|  | T5 | 0.01 (-0.05, 0.06) | 0.808 |  |  |  |  |  |  |
| **Complete at least 3 follow-ups (n=94)** | T1 | -0.01 (-0.06, 0.03) | 0.546 | 0.632 | 0.427 | 11.694 | **0.009** | 2.610 | 0.427 |
|  | T3 | 0.03 (-0.03, 0.10) | 0.284 |  |  |  |  |  |  |
|  | T4 | 0.03 (-0.04, 0.09) | 0.419 |  |  |  |  |  |  |
|  | T5 | 0.02 (-0.04, 0.08) | 0.464 |  |  |  |  |  |  |
| **Complete all 4 follow-ups (n=56)** | T1 | -0.05 (-0.11, 0.01) | 0.094 | 0.077 | 0.782 | 10.690 | 0.014 | 5.684 | 0.128 |
|  | T3 | 0.02 (-0.07, 0.10) | 0.665 |  |  |  |  |  |  |
|  | T4 | 0.05 (-0.03, 0.13) | 0.223 |  |  |  |  |  |  |
|  | T5 | 0.01 (-0.07, 0.09) | 0.779 |  |  |  |  |  |  |


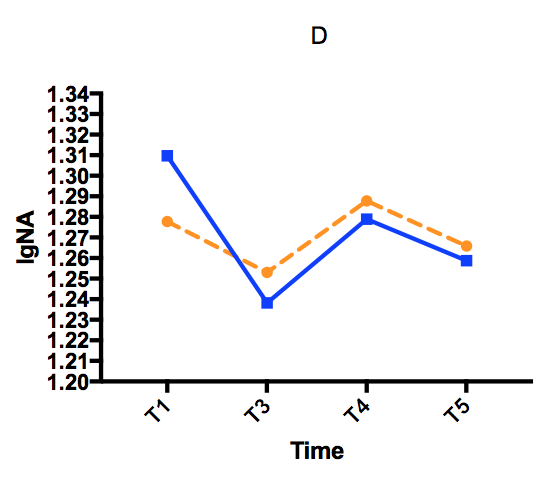

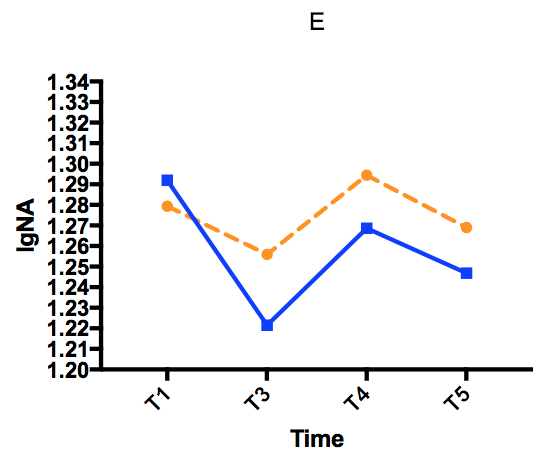

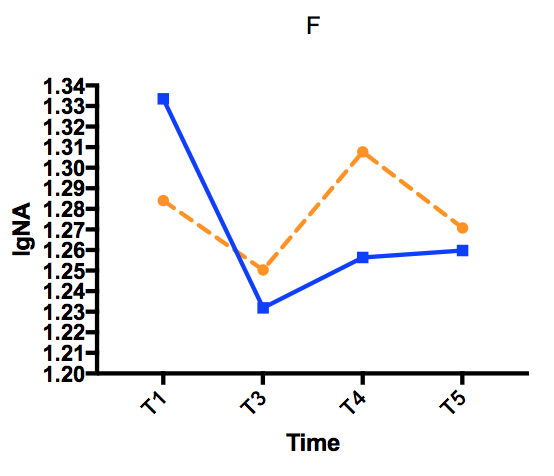

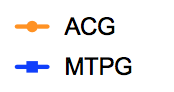


**Figure S33. Longer-term intervention effect on log-transformed NA in participants completed different times of follow-ups**

Note. D. complete at least 2 follow-ups; E. complete at least 3 follow-ups; F. complete all 4 follow-ups; * means significant between-group mean difference.

1. **PSQI**

**Table S38. Longer-term effect intervention effect on log-transformed PSQI** in participants completed different times of follow-ups.

|  |  | **MD _ACG-MTPG_** | ***p* value** | **Group effect** | | **Time effect** | | **Group × Time effect** | |
| --- | --- | --- | --- | --- | --- | --- | --- | --- | --- |
|  |  |  |  | **Wald *χ*^2^** | ***p* value** | **Wald *χ*^2^** | ***p* value** | **Wald *χ*^2^** | ***p* value** |
| **Complete at least 2 follow-ups (n=113)** | T1 | -0.02 (-0.09, 0.05) | 0.565 | 0.002 | 0.966 | 19.467 | **<0.001** | 2.280 | 0.516 |
|  | T3 | 0.04 (-0.055, 0.14) | 0.341 |  |  |  |  |  |  |
|  | T4 | 0.01 (-0.08, 0.10) | 0.827 |  |  |  |  |  |  |
|  | T5 | -0.03 (-0.13, 0.08) | 0.618 |  |  |  |  |  |  |
| **Complete at least 3 follow-ups (n=94)** | T1 | -0.01 (-0.09, 0.06) | 0.733 | 0.323 | 0.570 | 22.986 | **<0.001** | 3.839 | 0.279 |
|  | T3 | 0.08 (-0.02, 0.18) | 0.116 |  |  |  |  |  |  |
|  | T4 | 0.03 (-0.06, 0.12) | 0.559 |  |  |  |  |  |  |
|  | T5 | -0.01 (-0.12, 0.10) | 0.818 |  |  |  |  |  |  |
| **Complete all 4 follow-ups (n=56)** | T1 | 0.02 (-0.09, 0.12) | 0.762 | 0.857 | 0.355 | 21.701 | **<0.001** | 2.458 | 0.483 |
|  | T3 | 0.08 (-0.04, 0.20) | 0.183 |  |  |  |  |  |  |
|  | T4 | 0.01 (-0.10, 0.12) | 0.858 |  |  |  |  |  |  |
|  | T5 | 0.04 (-0.08, 0.17) | 0.487 |  |  |  |  |  |  |


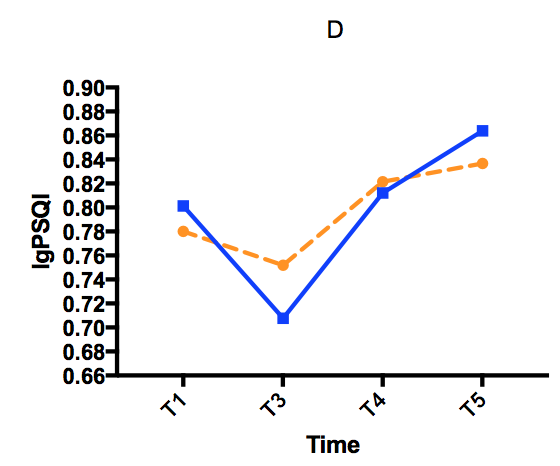

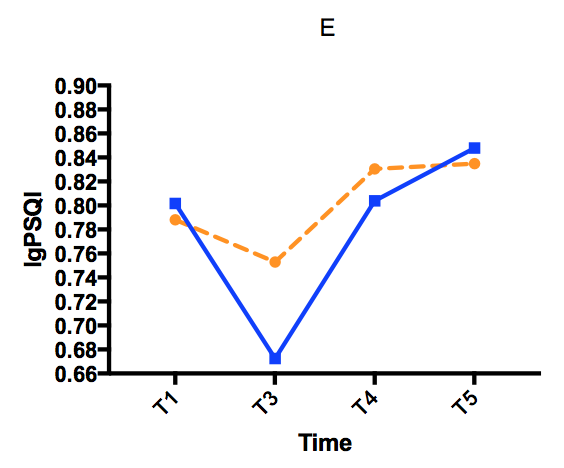

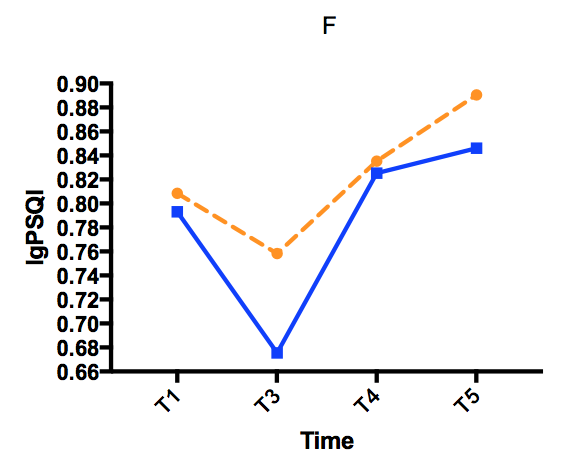

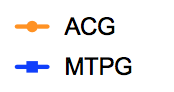


**Figure S34. Longer-term intervention effect on log-transformed PSQI in participants completed different times of follow-ups**

Note. D. complete at least 2 follow-ups; E. complete at least 3 follow-ups; F. complete all 4 follow-ups; * means significant between-group mean difference.

1. **FSS**

**Table S39. Longer-term effect intervention effect on FSS** in participants completed different times of follow-ups.

|  |  | **MD _ACG-MTPG_** | ***p* value** | **Group effect** | | **Time effect** | | **Group × Time effect** | |
| --- | --- | --- | --- | --- | --- | --- | --- | --- | --- |
|  |  |  |  | **Wald *χ*^2^** | ***p* value** | **Wald *χ*^2^** | ***p* value** | **Wald *χ*^2^** | ***p* value** |
| **Complete at least 2 follow-ups (n=113)** | T1 | 0.92 (-2.70, 4.54) | 0.617 | 0.735 | 0.391 | 12.381 | **0.006** | 1.051 | 0.789 |
|  | T3 | 1.51 (-2.63, 5.65) | 0.474 |  |  |  |  |  |  |
|  | T4 | 2.30 (-1.57, 6.17) | 0.243 |  |  |  |  |  |  |
|  | T5 | 0.04 (-4.53, 4.61) | 0.987 |  |  |  |  |  |  |
| **Complete at least 3 follow-ups (n=94)** | T1 | -0.54 (-4.35, 3.28) | 0.783 | 0.325 | 0.568 | 12.184 | 0.007 | 2.397 | 0.494 |
|  | T3 | 2.32 (-2.18, 6.81) | 0.312 |  |  |  |  |  |  |
|  | T4 | 1.88 (-2.15, 5.91) | 0.361 |  |  |  |  |  |  |
|  | T5 | -0.20 (-5.03, 4.64) | 0.936 |  |  |  |  |  |  |
| **Complete all 4 follow-ups (n=56)** | T1 | -0.24 (-4.96, 4.49) | 0.922 | 0.050 | 0.824 | 10.226 | **0.017** | 0.483 | 0.923 |
|  | T3 | -1.76 (-7.38, 3.86) | 0.539 |  |  |  |  |  |  |
|  | T4 | 0.19 (-4.86, 5.24) | 0.942 |  |  |  |  |  |  |
|  | T5 | 0.02 (-6.16, 6.20) | 0.995 |  |  |  |  |  |  |


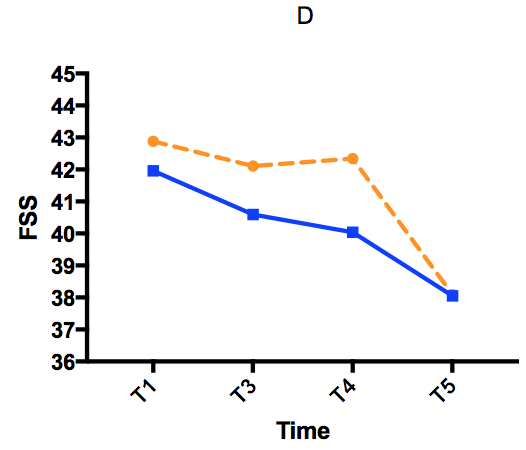

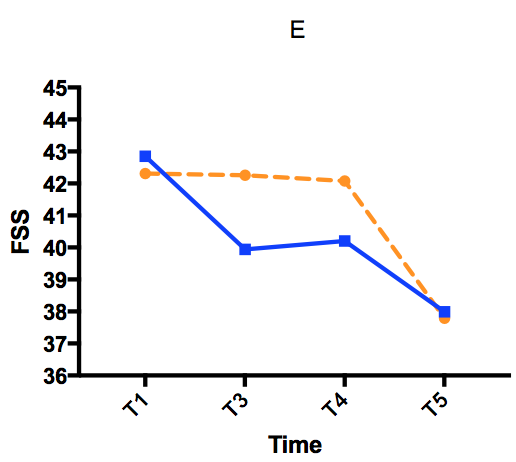

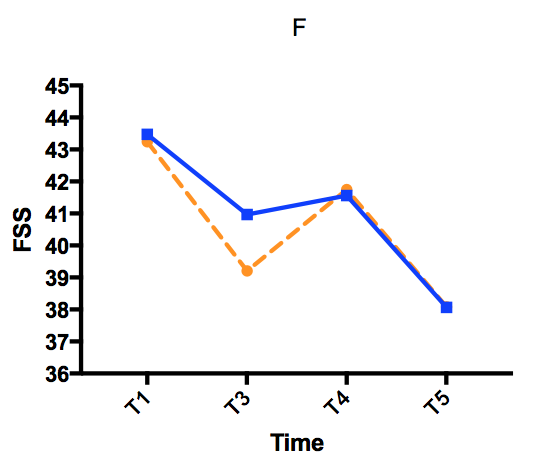

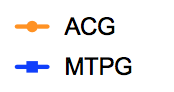


**Figure S35. Longer-term intervention effect on FSS in participants completed different times of follow-ups**

Note. D. complete at least 2 follow-ups; E. complete at least 3 follow-ups; F. complete all 4 follow-ups; * means significant between-group mean difference.

1. **PM**

**Table S40. Longer-term effect intervention effect on log-transformed PM** in participants completed different times of follow-ups.

|  |  | **MD _ACG-MTPG_** | ***p* value** | **Group effect** | | **Time effect** | | **Group × Time effect** | |
| --- | --- | --- | --- | --- | --- | --- | --- | --- | --- |
|  |  |  |  | **Wald *χ*^2^** | ***p* value** | **Wald *χ*^2^** | ***p* value** | **Wald *χ*^2^** | ***p* value** |
| **Complete at least 2 follow-ups (n=112)** | T1 | -0.01 (-0.06, 0.04) | 0.679 | 1.635 | 0.201 | 1.981 | 0.371 | 2.206 | 0.332 |
|  | T3 | -0.05 (-0.11, 0.01) | 0.128 |  |  |  |  |  |  |
|  | T5 | -0.04 (-0.12, 0.04) | 0.280 |  |  |  |  |  |  |
| **Complete at least 3 follow-ups (n=94)** | T1 | -0.02 (-0.07, 0.04) | 0.507 | 2.141 | 0.143 | 0.967 | 0.617 | 1.896 | 0.388 |
|  | T3 | -0.05 (-0.12, 0.01) | 0.112 |  |  |  |  |  |  |
|  | T5 | -0.05 (-0.14, 0.03) | 0.203 |  |  |  |  |  |  |
| **Complete all 4 follow-ups (n=56)** | T1 | -0.03 (-0.10, 0.03) | 0.326 | 4.028 | **0.045** | 0.069 | 0.966 | 2.899 | 0.235 |
|  | T3 | **-0.10 (-0.18, -0.01)** | **0.029** |  |  |  |  |  |  |
|  | T5 | -0.08 (-0.19, 0.02) | 0.122 |  |  |  |  |  |  |


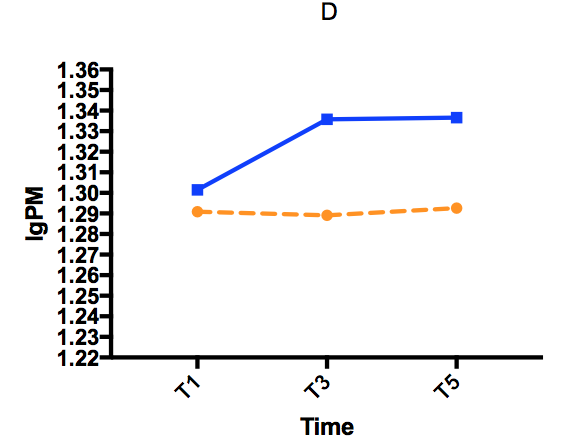

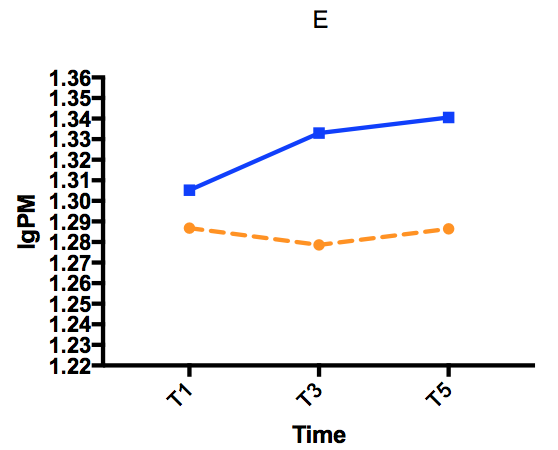

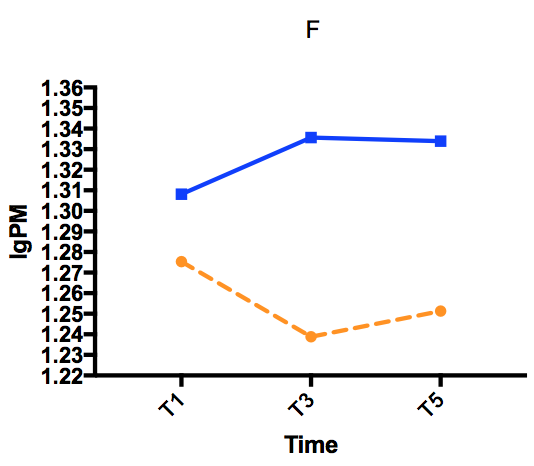

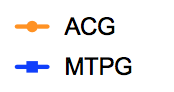


**Figure S36. Longer-term intervention effect on log-transformed PM in participants completed different times of follow-ups**

Note. D. complete at least 2 follow-ups; E. complete at least 3 follow-ups; F. complete all 4 follow-ups; * means significant between-group mean difference.

1. **RM**

**Table S41. Longer-term effect intervention effect on RM** in participants completed different times of follow-ups.

|  |  | **MD _ACG-MTPG_** | ***p* value** | **Group effect** | | **Time effect** | | **Group × Time effect** | |
| --- | --- | --- | --- | --- | --- | --- | --- | --- | --- |
|  |  |  |  | **Wald *χ*^2^** | ***p* value** | **Wald *χ*^2^** | ***p* value** | **Wald *χ*^2^** | ***p* value** |
| **Complete at least 2 follow-ups (n=112)** | T1 | 0.12 (-2.19, 2.42) | 0.922 | 0.365 | 0.546 | 13.334 | **0.001** | 1.585 | 0.453 |
|  | T3 | -1.06 (-3.82, 1.71) | 0.453 |  |  |  |  |  |  |
|  | T5 | -1.21 (-4.66, 2.23) | 0.490 |  |  |  |  |  |  |
| **Complete at least 3 follow-ups (n=94)** | T1 | -0.26 (-2.79, 2.28) | 0.843 | 0.651 | 0.420 | 12.048 | **0.002** | 1.234 | 0.540 |
|  | T3 | -1.19 (-4.26, 1.88) | 0.448 |  |  |  |  |  |  |
|  | T5 | -1.74 (-5.34, 1.85) | 0.342 |  |  |  |  |  |  |
| **Complete all 4 follow-ups (n=56)** | T1 | -1.73 (-4.65, 1.19) | 0.246 | 2.815 | 0.093 | 4.584 | 0.101 | 0.771 | 0.680 |
|  | T3 | -2.77 (-6.61, 1.07) | 0.157 |  |  |  |  |  |  |
|  | T5 | -3.15 (-7.38, 1.09) | 0.146 |  |  |  |  |  |  |


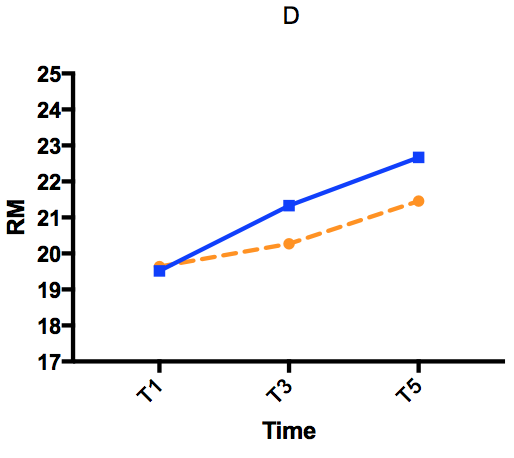

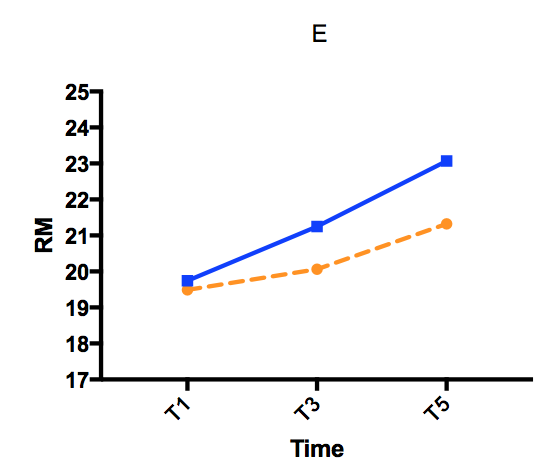

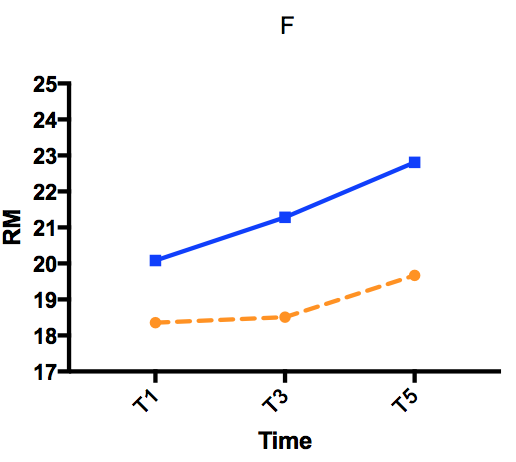

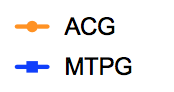


**Figure S37. Longer-term intervention effect on RM in participants completed different times of follow-ups**

Note. D. complete at least 2 follow-ups; E. complete at least 3 follow-ups; F. complete all 4 follow-ups; * means significant between-group mean difference.

1. **WDEQ**

**Table S42. Longer-term effect intervention effect on WDEQ** in participants completed different times of follow-ups.

|  |  | **MD _ACG-MTPG_** | ***p* value** | **Group effect** | | **Time effect** | | **Group × Time effect** | |
| --- | --- | --- | --- | --- | --- | --- | --- | --- | --- |
|  |  |  |  | **Wald *χ*^2^** | ***p* value** | **Wald *χ*^2^** | ***p* value** | **Wald *χ*^2^** | ***p* value** |
| **Complete at least 2 follow-ups (n=113)** | T1 | 2.31 (-4.21, 8.84) | 0.488 | 1.766 | 0.184 | 7.433 | 0.059 | 3.378 | 0.337 |
|  | T2 | 6.57 (-1.05, 14.19) | 0.091 |  |  |  |  |  |  |
|  | T3 | 6.02 (-1.10, 13.14) | 0.097 |  |  |  |  |  |  |
|  | T4 | 0.92 (-6.84, 8.68) | 0.816 |  |  |  |  |  |  |
| **Complete at least 3 follow-ups (n=94)** | T1 | 4.20 (-3.01, 11.41) | 0.254 | 2.615 | 0.106 | 6.812 | 0.078 | 2.213 | 0.529 |
|  | T2 | 6.28 (-2.22, 14.78) | 0.147 |  |  |  |  |  |  |
|  | T3 | **8.08 (0.08, 16.09)** | **0.048** |  |  |  |  |  |  |
|  | T4 | 2.97 (-5.33, 11.27) | 0.483 |  |  |  |  |  |  |
| **Complete all 4 follow-ups (n=56)** | T1 | 4.65 (-4.04, 13.35) | 0.294 | 2.832 | 0.092 | 12.835 | **0.005** | 1.001 | 0.801 |
|  | T2 | 8.85 (-1.13, 18.84) | 0.082 |  |  |  |  |  |  |
|  | T3 | 7.59 (-1.95, 17.14) | 0.119 |  |  |  |  |  |  |
|  | T4 | 6.60 (-3.37, 16.56) | 0.194 |  |  |  |  |  |  |


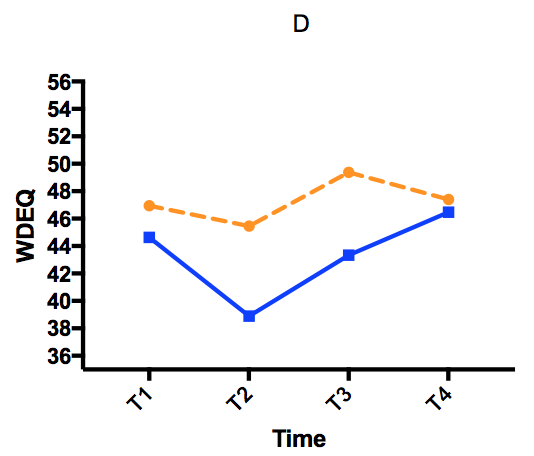

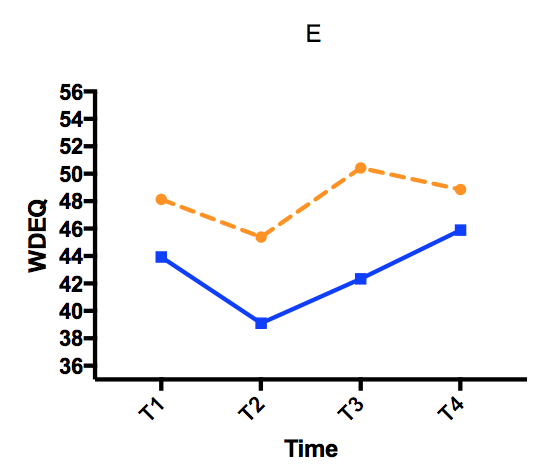

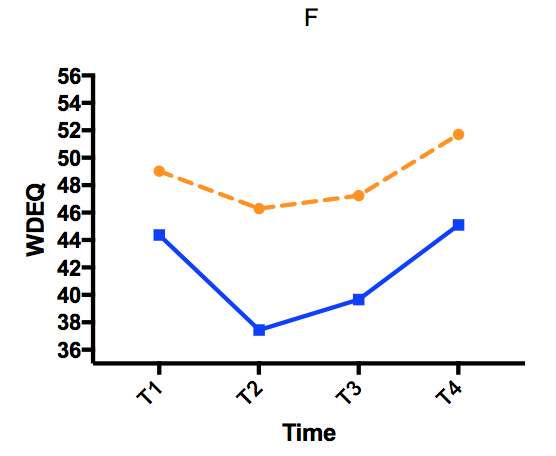

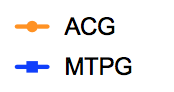


**Figure S38. Longer-term intervention effect on WDEQ in participants completed different times of follow-ups**

Note. D. complete at least 2 follow-ups; E. complete at least 3 follow-ups; F. complete all 4 follow-ups; * means significant between-group mean difference.
